# Characterization of Pseudorabies Virus Associated with Severe Respiratory and Neuronal Signs in Old Pigs

**DOI:** 10.1155/2023/8855739

**Published:** 2023-02-28

**Authors:** Hongjian Chen, Jie Fan, Xiuxiu Sun, Rui Xie, Wenbo Song, Yanxia Zhao, Ting Yang, Yan Cao, Shengwei Yu, Chunyan Wei, Lin Hua, Xiangru Wang, Huanchun Chen, Zhong Peng, Guofu Cheng, Bin Wu

**Affiliations:** ^1^State Key Laboratory of Agricultural Microbiology, The Cooperative Innovation Center for Sustainable Pig Production, College of Veterinary Medicine, Huazhong Agricultural University, Wuhan, China; ^2^Department of Basic Veterinary Medicine, College of Veterinary Medicine, Huazhong Agricultural University, Wuhan, China; ^3^Hubei Hongshan Laboratory, Wuhan, China

## Abstract

Pseudorabies virus (PRV) represents a leading threat to the global pig industry. Generally, pigs exhibit a pronounced age resistance against PRV, and the virus generally does not cause severe clinical signs and even death in old pigs. However, we characterized two PRV strains (HeN21 and HuB20) associated with severe respiratory and neuronal signs in old pigs. Among these two strains, HeN21 was isolated from the tonsil of a 24-week-old pig that died from severe neuronal and respiratory signs in a PRV-outbreak farm where a commercial PRV attenuated vaccine developed based on a PRV variant was used; while, HuB20 was isolated from the lung and lymph node of a 20-week-old with symptoms in another farm where Bartha-K61 vaccine was used. *In vitro* evaluations in different cell models demonstrated that HeN21 and HuB20 led to similar cytotoxic effects to those caused by PRV variants on PK-15, Vero, and SK-N-SH cells after 30 hours of inoculation. However, HeN21 possessed a higher titer than the other PRV variants from the first to the fifth passage on PK-15 cells and induced plaques with larger size. *In vivo* assessments in mouse and fattening pig models showed that inoculations of HeN21 and HuB20 caused higher morbidity and mortality and severe pathological damages in tested animals. In particular, challenge of HeN21 led to severe respiratory and neuronal signs in 90-day-old pigs. These two strains displayed higher virus loads on the main organs of challenged mice and pigs. Phylogenetic analysis revealed that HeN21 and HuB20 belonged to genotype II. In addition, recombinant events were identified in the genomes of HeN21 and HuB20, and several events were located within genes associated with PRV virulence. Our data herein may suggest the emergence of novel PRV strains in China.

## 1. Introduction

Pseudorabies virus (PRV) is a double-strand DNA virus belonging to the family Herpesviridae and subfamily Alphaherpesvirinae[1]. The virus possesses a 143 kb genome which encodes 70–100 proteins, including those glycoproteins necessary for viral multiplication (gB, gD, gH, gL, and gK) as well as those that determine the viral virulence (gC, gE, gL, gG, gM, and gN) [2]. Among them, the coding gene of gC was proposed as the marker for genotyping of PRV, and based on this gene, PRV strains were commonly typed as two genotypes (I and II) [3]. Generally, PRV is an important veterinary pathogen which mainly threats global pig industry, leading to reproductive failure, respiratory disorders, and death [4]. However, a couple of human cases with suspected PRV infection have been reported in China since 2017 and a virus strain has been finally isolated from the cerebrospinal fluid (CSF) samples of a patient [5], suggesting the virus might possess public health risks.

The first report of a PRV-outbreak in China occurred in the 1950s, and the widespread use of an attenuated vaccine derived from PRV Bartha-K61 and the other classical Chinese virus strains such as Ea controlled the outbreaks of pseudorabies well in the country between 1990 and 2011 [6, 7]. However, since the late 2011, novel PRV variants (e.g., JS-2012, HB1201, and HeN1) have emerged and circulated in many Chinese pig farms, even in those that had vaccinated pigs with the Bartha-K61 vaccine in the late 2011 [8–11]. Many laboratory studies have demonstrated that PRV variants are more virulent than the classical strains (e.g., Ea, Fa, and SC) toward older pigs (35–127 days) [12]. It is worthy of note that pigs generally exhibit a pronounced age resistance against PRV, with younger pigs more susceptible to fatal infections characterized by neuronal signs, while older pigs (>1 years) frequently present with respiratory distress or subclinical infections [13]. Severe signs of the central nervous system (CNS) involvement as well as severe respiratory disorders and death due to PRV infection are rarely seen in pigs older than 6 weeks [13]. In this study, we reported the isolation and pathogenicity of a PRV (the strain HeN21) from a 24-week-old pig that died from severe neuronal and respiratory signs in a PRV-outbreak farm in China. *In vitro* and *in vivo* tests showed that this isolate demonstrated higher virulence than tested PRV variants and it could lead to severe neuronal and respiratory signs in 90-day-old pigs. In addition, we also isolated a recombinant PRV strain from fattening pigs in a farm in China. *In vivo* and *in vitro* tests demonstrated that this strain was also virulent to older pigs. The isolation of these two strains may indicate the emergence of a PRV strain with higher virulence in China.

## 2. Materials and Methods

### 2.1. PRV Strains, Cells, and Genome Sequences

The PRV strain HeN21 was isolated from the tonsil of a 24-week-old pig that died from severe neuronal and respiratory signs in a PRV-outbreak farm in China in 2021; HuB20 and HBJZ-44-2021 were isolated from the lung and lymph node of two pigs with symptoms in two pig farms in Central China in 2020 and 2021, respectively; JSZL-2018 was isolated from the brain of a baby pig in Eastern China in 2018. Our previously collected PRV strains, including two variants (HuB1/CHN2017 and SMX-2012) and two classical strains (Ea and Bartha-K61) [14], were also included as control viruses in this study. A total of 28 genome sequences downloaded from NCBI were used as reference genomes for bioinformatical analysis (Table S1). Cells used in this study included PK-15 cells (ATCC CCL-33), Vero cells (ATCC CCL-81), and SK-N-SH (ATCC HTB-11). Among them, PK-15 and Vero cells were maintained in Dulbecco's modified eagle medium (DMEM; Gibco, US) supplemented with 2% fetal calf serum (Gibco, US), while SK-N-SH cells were cultured using minimum essential medium (MEM; Procell, China) supplemented with 2% fetal calf serum.

Virus isolation was performed as described previously [6]. In brief, tissues were minced, and tissue samples were minced, immersed with DMEM, and homogenized using a QIAGEN TissueLyser II (QIAGEN Germany). Sample homogenates were then frozen at −80°C and thawed three times. After that, homogenates were centrifuged at 12,000 rpm for 5 min, followed by filtration of the supernatants using a 0.22 mm membrane. The filtrates were then inoculated into PK-15 cells and were incubated at 37°C. Cells with the obvious cytopathic effect (CPE) were used for plaque purification assays. Plaques with suitable size were selected and inoculated into 500 *μ*L DMEM, frozen, and thawed three times and then diluted 2-fold in DMEM for the second round of the plaque purification assay. Finally, plaque fluid was inoculated into PK-15 cells and cultured in a flask.

### 2.2. Cytotoxicity

To evaluate the cytotoxicity, PRV strains were passaged stably in PK-15 cells, and the 50% tissue culture infective dose (TCID_50_) of each of the PRV strains was determined. Thereafter, different PRV strains (MOI = 0.1) were inoculated into monolayers of different cells (PK-15, Vero, and SK-N-SH) on a 6-well plate (Corning, Corning, US), incubated at 37°C under 5% CO_2_. After adsorption for 2 h, the media were discarded and fresh maintenance media were added (defined as 0-hour postinoculation (hpi)). Samples were collected at different time points (2, 4, 6, 12, 18, 24, 30, 36 hpi) for virus titering to determine the one-step curve. At 30 hpi, cells incubated with each of the virus strains were stained using crystal violet, and ten plaques were randomly selected for size determination using the Image J software.

### 2.3. Whole Genome Sequencing and Bioinformatical Analysis

Whole genome sequencing was performed as previously described [14]. Viral DNA was extracted using a commercial DNA/RNA extraction kit (Vazyme, Nanjing, China) and was checked for quality and quantity by agarose gel electrophoresis and using a Qubit® 2.0 Fluorometer (Thermo Scientific, US). Sequencing libraries were prepared using the Nextera™ DNA Flex Library Prep Kit (Illumina, CA) following the manual and were sequenced on the Illumina HiSeq2500 platform. The paired-end sequencing gave a 150 bp reading length from each terminal. This strategy yielded a total of 1,982 Mb raw data for HeN21 (coverage: 13,708.91×) and 1,190 Mb raw data for HuB20 (coverage: 7,933.33×). Thereafter, data with low quality were filtered. Through this approach, a total of 1,774 Mb clean data were obtained for HeN21 (Q30: 90.71%) and a total of 988 Mb clean data were obtained for HuB20 (Q30: 90.74%). The data were then assembled using SPAdes (version 3.15.4) [15], and a 144,608 bp genome sequence was finally generated for HeN21 (a *G* + *C* content of 73.68%) and 143,032 bp for HuB20 (a *G* + *C* content of 72.06%).

Genome sequence was annotated using RAST Server [16]. Sequence alignments were performed by using the EasyFig package (version 2.2.3_win) [17] and/or the MAFFT package (version 7.471) [18]. Nucleotide similarity at the genome level was calculated and visualized by using the SimPlot software (version 3.5.1), and the data were regenerated by using GraphPad Prism 8. Average nucleotide identity (ANI) between two nucleotide sequences was calculated using an ANI calculator [19]. GeneDoc (version 2.7) was used to visualize the sequence alignments of genes or proteins. Phylogenetic analysis was performed using the MEGA software (version 11) [20], based on the nucleotide sequence alignments of gC, gB, gE, and whole genome sequences, respectively. A bootstrap value of 1000 was also applied, and the tree was visualized by using the iTOL tool [21]. Recombinant events were detected by using Recombination Detection Program (RDP) package Beta 4.100 [22]. A recombination event was considered to be reliable if it was detected by at least three out of the seven selected algorithms (RDP, GENECONV, BootScan, Maxchi, Chimaera, SiScan, and 3Seq) with a significance of *P*  <  0.01 [23].

### 2.4. Animal Tests and Ethic Statements

To evaluate the virulence of the virus, 5-6-week-old SPF BALB/*c* mice (*n* = 50) were divided into five groups (G1–G5) and each group contained 10 mice. Each mouse in different groups was challenged with PRV HeN21 (G1, 10^4^ TCID_50_ per mouse), HuB20 (G2, 10^4^ TCID_50_ per mouse), JSZL-2018 (G3, 10^4^ TCID_50_ per mouse), HBJZ-44-2021 (G4, 10^4^ TCID_50_ per mouse), and DMEM (G5, 50 *μ*l per mouse), respectively. After challenge, death condition was recorded to generate the survival curves. In parallel, 48 mice were divided into eight groups (G6–G13) and each group contained six mice. Each mouse in G6–G13 was challenged with PRV HeN21, HuB20, JSZL-2018, HBJZ-44-2021, HuB1/CHN2017, SMX-2012, Ea, and Bartha-K61 at a dose of 10^4^ TCID_50_, respectively. After challenge, the dying mice were euthanized and dissected. The murine hearts, livers, spleens, lungs, kidneys, and brains were collected to detect viral loads using real-time quantitative PCR (qPCR) with the primer “gH-F: ACGTTCGGCTTCCTCTCC,” “gH-R: GGTAGTCGTCGCTCTCGTG,” and “gH-P: -FAM-TCGCGCATCGTCTGGTGCAT-BHQ1.” DNA copies were calculated according to the formula *C*_*t*_ = −3.543 × l g DNA copies + 44.22 (*R*^2^ = 0.9993). The murine lungs and brains were also arranged for histological examination using hematoxylin-eosin staining. A score reflecting the pathological damages was given based on the result of histological examination, as described previously [24].

The virulence of PRV strains was also assessed using fattening pigs. In this experiment, 90-day-old pigs (*n* = 12) were divided into four groups (GP1–GP4) and each group contained three pigs. Before challenge, swine blood, as well as blood, nose and mouth swabs, and anal swabs were collected for the detection of PRV antibodies and viral DNA to ensure all pigs were negative for PRV. Thereafter, each pig in different groups was inoculated with PRV HeN21 (GP1, 2 × 10^7^ TCID_50_ per pig), HuB20 (GP2, 2 × 10^7^ TCID_50_ per pig), SMX-2012 (GP3, 2 × 10^7^ TCID_50_ per pig), and DMEM (GP4, 2 ml per pig) through the intranasal routine. After inoculation, swine rectal temperatures as well as death conditions were recorded every day for 15 days. Nose and mouth swabs and anal swabs were collected every day postinoculation, as well as blood was collected every two days postinoculation to detect the virus load using qPCR with the abovementioned primer. Different types of tissues (the heart, liver, spleen, lung, cerebrum, kidney, cerebrum, cerebellum, tonsil, submandibular lymphatic node, hilar lymph node, and inguinal lymph node) of pigs from different groups were collected for viral DNA detection and histological examination using hematoxylin-eosin staining. Based on the result of histological examination, a score reflecting the pathological damages was given according to a previous publication [24].

Animal tests were performed at the Laboratory Animal Center of Huazhong Agricultural University (Wuhan, China) and were approved by the University Ethics Committee. The approval ID numbers were HZAUMO-2022-0151 for the mouse experiment and HZAUSW-2022-0018 for the pig experiment. During the experiment, animals were carried out under the guidelines established by the China Regulations for the Administration of Affairs Concerning Experimental Animals (1988) and Regulations for the Administration of Affairs Concerning Experimental Animals in Hubei province (2005).

### 2.5. Statistical Analysis

Statistical analysis was conducted using the “Multiple *t*-tests” strategy in GraphPad Prism 6.0. Data represent mean ± SD. The significance level was set at *P* < 0.05 (^*∗*^) and *P* < 0.01 (^*∗∗*^).

## 3. Results

### 3.1. Case Description and Isolation of PRV HeN21

**Figure 1:**
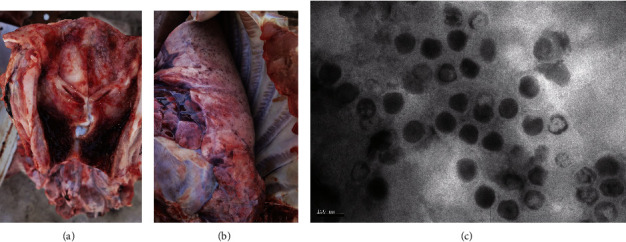
Isolation and characterization of pseudorabies virus HeN21. (a) Damage of the tonsil of the pig that pseudorabies virus (PRV) HeN21 was isolated. (b) Damage of the lung of the pig that PRV HeN21 was isolated. (c) Morphological characteristics of PRV HeN21 under an electron microscope (bar = 100 nm).

In December 2021, PRV infections occurred in a fattening pig farm in Central China. Pigs reared in this farm were generally 24-week-old (six months), and the average body weight of each pig was around 100 kg. Before death, many pigs with infection showed signs of severe respiratory distress with neuronal signs. However, all pigs in this farm had been immunized with a commercial PRV-attenuated live vaccine developed based on a variant strain three times following the recommended dose at the first day after birth, 35-day-old, and 80-day-old, respectively. After disease outbreak, an urgent immunization was given using the same commercial PRV-attenuated live vaccine with increased doses. This urgent vaccination helped to decrease the death, but many pigs still exhibited cough and fever. Dissection of pigs showed ulcerated tonsils and hemorrhagic lungs (Figures 1(a), 1(b)). Finally, a PRV strain, designated HeN21, was isolated from the tonsil (Figure 1(c)).

### 3.2. Biological Characteristics of PRV Isolates

**Figure 2:**
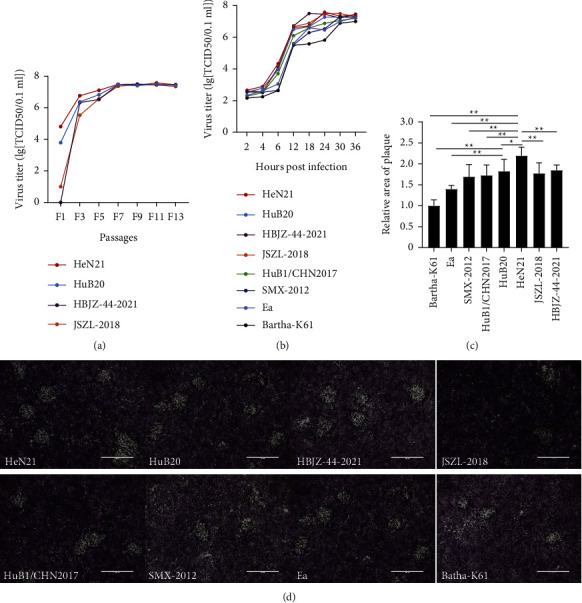
Biological characteristics of different pseudorabies virus strains. (a) Virus titers of different pseudorabies virus (PRV) strains at different passages on PK-15 cells. (b) One-step growth curves of different pseudorabies virus (PRV) strains at different passages on PK-15 cells. (c) Plaques caused by different PRV strains on PK-15 cells observed under an electron microscope (bar = 1000 *μ*m). (d) Sizes of plaques caused by different PRV strains on PK-15 cells quantified using the ImageJ software.

Cytotoxicity tests on different cell models showed that HeN21, as well as HuB20, HBJZ-44-2021, and JSZL-2018, led to similar cytotoxic effects to those caused by PRV variants (HuB1/CHN2017 and SMX-2012) on PK-15, Vero, and SK-N-SH cells after 30 hours of inoculation; the effects included cells became round, falling off, and syncytia occurred (Figure S1). Determination of virus titers revealed that the titer of HeN21 was higher than the other PRV strains (HuB20, HBJZ-44-2021, and JSZL-2018) from the first passage (F1) to the fifth passage (F5) on PK-15 cells, but these four strains finally had similar and stable titers in the range 10^8.40–8.60^. TCID_50_/ml after the seventh passage on the cells (Figure 2(a) and Table S2). One-step growth curve tests demonstrated that the PRV HeN21, HuB20, HBJZ-44-2021, and JSZL-2018 displayed a similar growth condition to PRV variants (HuB1/CHN2017 and SMX-2012) and classical strains (Ea and Bartha-K61) (Figure 2(b)). However, HeN21 led to plaques with larger size than those formed by the other virus strains on PK-15 cells (Figures 2(c) and 2(d)).

### 3.3. Virulence of PRV Strains on Mice and Old Pigs

**Figure 3:**
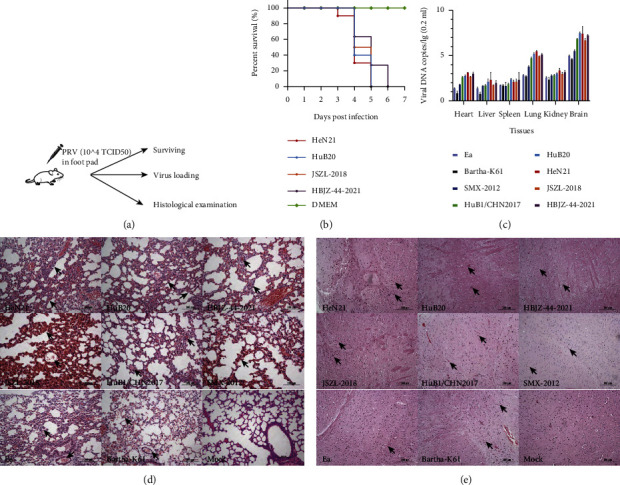
Evaluation of viral virulence using mouse models. (a) Study design of mouse experiment. (b) The survival curve of experimental mice challenged with different pseudorabies virus (PRV) strains. (c) Detection of viral DNA in different murine tissues by real-time quantitative PCR. (d) Histological examinations of the murine lungs from different challenged mice (bar = 100 *μ*m). (e) Histological examinations of murine brains from different challenged mice (bar = 200 *μ*m).

To evaluate the virulence of PRV, mice were inoculated with different PRV strains or DMEM in foot pad (Figure 3(a)). Considering the passages of different PRV strains, we only compared the survival curves caused by HeN21, as well as HuB20, HBJZ-44-2021, and JSZL-2018. The approach demonstrated that inoculation of these four PRV strains led to all mice death within 6 days postinoculation, but inoculation of HeN21 led to earlier death in mice (Figure 3(b)). Detection of virus load on different organs showed that PRV DNA was detectable in the murine heart, liver, spleen, lung, kidney, and brain after inoculation, but the brain and lung possessed the highest copies of detection (Figure 3(c)). In addition, the detection of DNAs of HeN21 and HuB20 in these organs was higher than those of the other PRV strains (Figure 3(c) and Table S3). Histological examinations showed that inoculations of PRV strains caused severe damages on the lungs and brains; the lungs were hemorrhagic and congestive, the alveolar walls were thickened, the alveolar epithelial cells were necrotic, and there were exudations in the alveolar cavities (Figure 3(d)). Damages on brains included hemorrhage and congestion, neuronal degeneration, inflammatory cell infiltration, and small vessel hyperplasia (Figure 3(e)). Pathological injury scoring revealed that HN21 resulted in the highest injury scores on the lungs and brains, followed by the strain HuB20 (Table S4).

**Figure 4:**
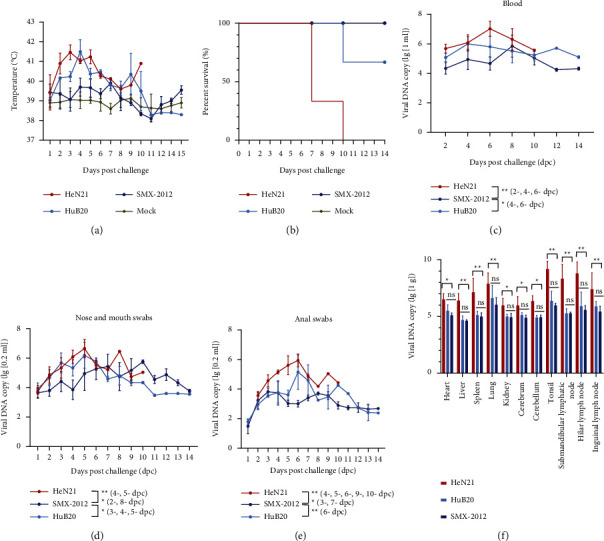
Evaluation of viral virulence using old pig models. (a) Rectal temperature of pigs inoculated with different pseudorabies virus (PRV) strains or PBS at different days post inoculation. (b) The survival curve of experimental pigs inoculated with different PRV strains or PBS. (c) Detection of viral DNA in blood collected from pigs inoculated with different PRV strains at different days after inoculation by real-time quantitative PCR. (d) Detection of viral DNA in the nose and mouth swabs collected from pigs inoculated with different PRV strains at different days post inoculation by real-time quantitative PCR. (e) Detection of viral DNA in anal swabs collected from pigs inoculated with different PRV strains at different days after inoculation by real-time quantitative PCR. (f) Detection of viral DNA in different pig tissues by real-time quantitative PCR.

We next tested the pathogenicity of HeN21, HuB20, and SMX-2012 in fattening pigs. After challenge, all pigs inoculated with PRV displayed an increased rectal temperature, but pigs challenged with HeN21 and HuB20 showed higher rectal temperatures than those inoculated with SMX-2012 within 10 dpc (Figure 4(a)). In the HeN21 challenging group, pigs exhibited severe respiratory symptoms (swollen face, abdominal breathing, and purulent mucus nasal discharge) and difficulty in feeding at 4 days postchallenge (dpc), and three pigs died at 7 dpc (2 pigs) and 10 dpc (1 pig), respectively (Figure 4(b)). The inoculation of HuB20 led to one pig died in 10 dpc, and the survival pigs as well as those challenged with SMX-2012 only demonstrated mild respiratory symptoms. A highest level of viral DNA was detected from the blood, nose and mouth swabs, and anal swabs of pigs challenged with HeN21 compared to those from pigs challenged the other PRV strains (Figures 4(c)–4(e)). In HeN21 challenged pigs, a highest level of viral DNA was detected in tonsils, which was higher than 10^9^ copies/g (Figure 4(f)).

**Figure 5:**
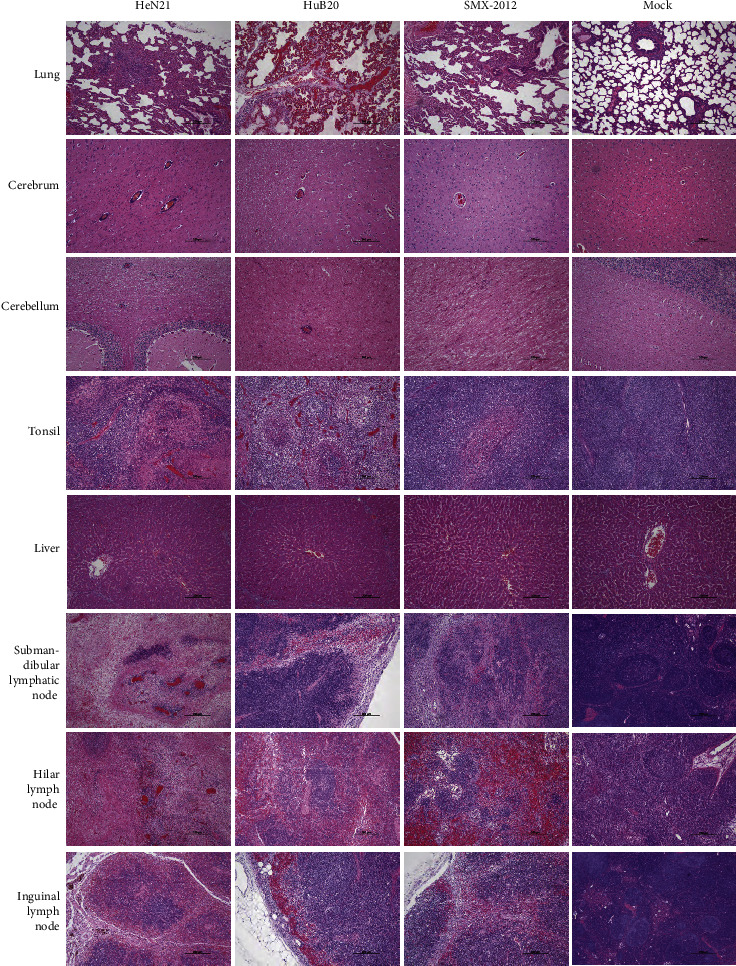
Histological examinations of different tissues of pigs inoculated with different pseudorabies virus (PRV) strains (bar = 200 *μ*m). Histological damages in the lungs included an infiltration of massive inflammatory cells in alveolar spaces as well as pulmonary interstitial fibrosis and thickening. In the brains, many blood vessels as well as degenerated and necrotic glial nodules and nerve cells were observed particularly those of pigs challenged with HeN21. Lymphocyte necrosis with a large number of red blood cells filled in lymph nodes; the structures of lymph nodes disappeared. Degeneration and necrosis of epithelial cells appeared in tonsils. Notably, challenging of HeN21 and HuB20 resulted in necrosis of liver cells and congestion in the central vein in the livers.

Dissection of PRV challenged pigs demonstrated the congestive and hemorrhagic brains, lungs, livers, and lymph glands compared to those of the PBS-treated pigs (Figure S2). Notably, infarcts were observed on the livers of pigs challenged with HeN21, while necrotic and pyogenic tonsils were observed in pigs challenged with HeN21 and HuB20, respectively (Figure S2). Histological damages in the lungs included an infiltration of massive inflammatory cells in alveolar spaces as well as pulmonary interstitial fibrosis and thickening (Figure 5). In the brains, many blood vessels as well as degenerated and necrotic glial nodules and nerve cells were observed, particularly those of pigs challenged with HeN21 (Figure 5). Lymphocyte necrosis with a large number of red blood cells filled in lymph nodes; the structures of lymph nodes disappeared (Figure 5). Degeneration and necrosis of epithelial cells appeared in tonsils (Figure 5). Notably, challenging of HeN21 and HuB20 resulted in necrosis of liver cells and congestion in the central vein in the livers (Figure 5). According to histopathological injury scoring, challenging of HeN21 caused most severe damages on different organs of pigs, followed by the challenging of HuB20 and SMX-2012, respectively (Table S5).

### 3.4. Phylogenetic Analysis and Recombination Analysis of PRV Strains

**Figure 6:**
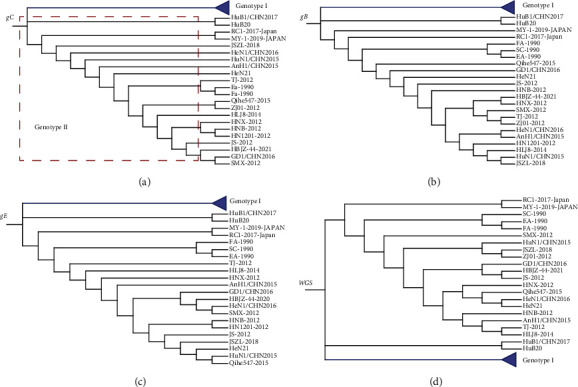
Phylogenetic analysis of different pseudorabies virus (PRV) strains. (a) A phylogenetic tree generated based on the nucleotide sequences of the *gC* gene. (b) A phylogenetic tree generated based on the nucleotide sequences of the *gB* gene. (c) A phylogenetic tree generated based on the nucleotide sequences of the *gE* gene. (d) A phylogenetic tree generated based on the whole genome sequences.

Phylogenetic analysis based on the gC gene showed that HeN21 and HuB20 belonged to genotype II, showing a close relationship with the other PRV Chinese isolates (Figure 6(a)). Phylogenetic analysis based on the gB gene, gE gene, and whole genome sequence also demonstrated a similar result, respectively (Figures 6(b)–6(d)). Phylogenetic analysis based on the whole genome sequence indicated that HeN21 was a variant strain with closest relationship with HeN1/CHN2016 (GenBank accession no. MK642577), a variant strain isolated in Henan province [14]. However, phylogenetic analysis based on gC, gB, gE, or the whole genome sequence showed that HuB20 and an another strain HuB1/CHN2017 (GenBank accession no. MK682670) were located on a separate evolutionary branch within the genotype II strains (Figures 6(a)–6(d)).

**Figure 7:**
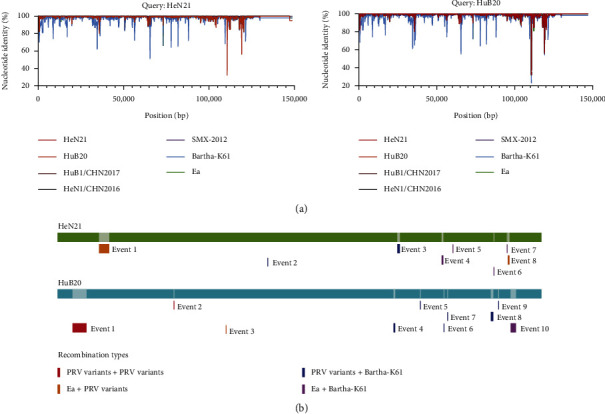
Comparative genomic analysis and recombination analysis of different pseudorabies virus (PRV) strains. (a) Nucleotide similarities of PRV HeN21 or HuB20 and the other PRV strains isolated from pigs. (b) Genomic recombinant analyses of the complete genome of PRV HeN21 and HuB20; the genome is shown with green (HeN21) and blue (HuB20). Recombination events predicted by RDP4 are showed as red (PRV variants + PRV variants), blue (PRV variants + Bartha-K61), orange (PRV variants + Ea), and purple (Ea + Bartha-K61),.

The whole genome sequence comparison demonstrated that the genome sequences of HeN21 and HuB20 were highly homologous to those of PRV variants isolated from China (Figure 7(a)). The ANI between the genome sequences of HeN21 and a previously reported Chinese PRV variant HeN1/CHN2016 was 99.74%; while, this value between the genome sequences of HuB20 and HuB1/CHN2017 was 99.87%. However, both of the whole genome sequences of HeN21 and HuB20 showed less homology to that of PRV Bartha-K61 (Figure 7(a)). In addition, several parts of the genomes of HeN21 also showed less homologies to those of HuB20 (Figure 7(a)). The amino acids of the main glycoproteins (gB, gC, gD, gE, gG, gH, gL, gM, gN, and gK) of HeN21 were also highly similar to those of the other Chinese PRV variants, but several amino acid mutations were identified in those proteins of HuB20 compared to those of the other PRV variants (Figures S3).

Recombinant analysis detected different types of recombinant events in the genome sequences of HeN21 and HuB20, including recombination between different PRV variants (PRV variants + PRV variants), recombination between PRV variants and vaccine strains (PRV variants + Bartha-K61), recombination between PRV variants and classical strains (PRV variants + Ea), and recombination between PRV classical strains and vaccine strains (Ea + Bartha-K61) (Figure 7(b)). Detected recombinant events in HeN21 were located within the nonglycoprotein-encoding regions, including UL47 (event 1), UL46 (event 1), EP0 (event 3), IE180 (event 3), US1 (event 4), and US3 (event 5) or the noncoding regions. However, detected recombinant events in HuB20 were located within UL53 (gK; event 1), UL52 (event 1), UL51 (event 1), UL50 (event 1), UL40 (event 3), IE180 (event 5), and US2 (event 8); while, other events were located within an inverted repeat region (events 4, 6, and 7).

## 4. Discussion

In this study, we characterized several PRV strains isolated from fattening pigs, and two of them (HeN21 and HuB20) demonstrated increased virulence compared to PRV variants isolated from pigs in China as evidenced by evaluations in both cell and animal models. While PRV may lead to severe clinical signs and even death in piglets less than 3-week-old, PRV strains, even the 2011-emerged variants are not likely to cause severe symptoms, particularly the respiratory and neuronal signs and even death in old pigs (≥90-day-old) as pigs generally exhibit a pronounced age resistance against PRV [13]. This documentation could be demonstrated by 90-day-old pigs challenged with SMX-2012; a PRV variant isolate from field, which could lead to severe respiratory signs and death in baby pigs [25], only showed mild respiratory symptoms in this study. However, inoculation of HeN21 and HuB20, particularly the first one, resulted in severe respiratory and neuronal signs and even death in experimental pigs. Notably, pigs in the farm where HeN21 was isolated had been immunized with attenuated live vaccine developed from PRV variants according to our questionnaire, and it seems the vaccination did not provide full protection as death still occurred. This phenomenon is quite similar to the identification of the PRV variants at the early stage of the 2011-outbreak [8, 11]. It is also notable that HeN21 and HuB20 were not just the few strains associated with severe symptoms and even death in old pigs in China. Recently, two publications from China have documented a PRV variant XJ5; inoculation of this strain at a dose of 2 × 10^6^ TCID_50_/pig intranasally could also induce respiratory symptoms as well as CNS symptoms and death in 11-12-week-old pigs [26, 27]. These characterization of abovementioned PRV variants may suggest the emergence of PRV with increased virulence in China.

Since the first report of a PRV-outbreak in China in the 1950s, the prevalence of PRV in Chinese pig industry may be divided into the following two experiences: (1) from 1950s to 2011, PRV classic strains such as Ea were mainly prevalent in the pig industry and the widespread use of an inactivated vaccine derived from PRV Bartha-K61 imported from Hungary helped to control the outbreaks of the disease well and [6, 7] (2) from the late 2011 to now, PRV variants (e.g., JS-2012, HB1201, and HeN1) emerged and became the main prevalent type and vaccination of the Bartha-K61 vaccine may not provide full protection against these strains [6, 8, 10, 11]. It is still not sure whether the continuous vaccination stimulates the emergence of PRV variants with increased virulence, but using other herpes virus as models, it has already confirmed that imperfect vaccination can promote the emergence of more virulent pathogens [28]. Therefore, there might be a possibility that the emergence of PRV variants with increased virulence compared to the classical strains in the late 2011 is associated with imperfect vaccination of vaccines between 1950s and 2011. Another notable point is that commercial vaccines developed from PRV variants became available after 2012 and displayed a good effect on combating PRV variants in many pig farms. Now ten years passed by, it could not rule out a possibility that a widespread application particularly the imperfect use of vaccines derived from the variants stimulates the emergence of novel epidemic strains. However, further studies from both clinical monitoring and laboratory research studies are necessary to confirm these possibilities.

While there is still no standard typing method available for PRV, a widely used method is classifying PRV strains into two genotypes (I and II) based on the sequence of gC [3]. A series of previous studies have documented that PRV strains circulating in China belong to genotype II, but those from Europe and North America including the vaccine strain Bartha-K61 are genotype I strains [3, 6, 14, 29, 30]. Consistently, our genotyping based on gC revealed that HeN21 and HuB20, together with other Chinese strains and even two strains isolated recently from Japan, belonged to genotype II. This finding was also confirmed by phylogenetic analyses using the sequences of gB, gE, and the whole genome sequences. It is worth of note that the host of HuB20 came from a farm where Bartha-K61 vaccines were used. Together with the records of several published articles [10, 31, 32], these data may explain the reduced efficacy of Bartha-K61 on combating PRV variants, i.e., the vaccine strain is phylogenetically distinct from the epidemic strains in China. Another interesting finding is that HuB20 and another strain HuB1/CHN2017 is located on a separate clade with the genotype II branch. Our previous study suggests HuB1/CHN2017 might be a recombinant strain [14]. Therefore, HuB20 might also be a recombinant one. To further verify this, we checked the recombinant events in HuB20, and the test indeed detected several recombinant events in the strain. Notably, the characterization of the susceptible PRV recombinant strain has been reported recently [33], and HuB20 displayed a phylogenetic characteristic similar to the susceptible PRV recombinant strain reported in this study. These findings suggest that HuB20 might be also a recombinant strain. Considering HuB20 could cause severe symptoms and even death in old pigs, it is of significance to monitor the prevalence of this type of PRV strains in the field.

While several mutations were determined in genes encoding main glycoproteins in HuB20, it is still hard to say whether these mutations are associated with the virulence. This is because there was no difference on the sequence of these glycoproteins between HeN21 and other PRV variants, but HeN21 still showed increased virulence compared to these variants. Similar results could be also seen in one of our recent works [34], in which we also found there is no difference on the sequence of main glycoproteins of the human-origin PRV strain hSD-1/2019 (GenBank accession no. MT468550) and pig-origin PRV variants, but human infections caused by pig-origin PRV variants are rare. Further studies may be necessary to explore the associations between amino acid mutations in glycoproteins and viral virulence. Genome recombination has been reported in PRV and other alpha-herpesviruses [1, 14, 33]. Here, we also detected several recombinant events in the genomes of both HeN21 and HuB20. These events involved in recombination between different types of PRV strains, and vaccine strains were found to be participating in many events. Since recombination has been well established to contribute significantly to the evolution of alpha-herpesvirus [1, 35], the recombination between different PRV strains, including those between the epidemic strains and the vaccine strains, might accelerate the emergence of novel strains. Notably, most of the recombinant events detected in HeN21 and HuB20 were located within genes involved in viral egress (UL53, UL51, and UL47), DNA replication (UL52), nucleotide synthesis (UL50 and UL40), gene expression regulation (US1, IE180, and EP0), and membrane-associated proteins (US2) [2]. It is also worthy of note that several of these genes have been reported to be associated with the virulence of PRV. For example, a previous study found that the UL50-negative PRV strain was attenuated when young pigs were inoculated intranasally [36]. Therefore, recombination occurred in several of these regions might have impacts on the virulence of PRV, but further studies are necessary to explore this.

## 5. Conclusions

To conclude, we report the characterization of two PRV variants with increased virulence in this study. Our laboratory challenging tests in fattening pigs showed that these two strains could induce severe respiratory and neuronal signs and even death in pigs at 90-day-old. Bioinformatic analysis identified recombinant events in the genomes of these two strains, and the fragments at the recombinant sites were likely from either the field epidemic strains or the vaccine strains. Our data present herein may suggest the emergence of novel PRV strains in China.

## Data Availability

Whole genome sequences of PRV strains HeN21 and HuB20 were deposited into the NCBI GenBank database. Accession numbers are OP906304 for PRV strain HeN21 and OP906305 for PRV strain HuB20.

## Supplementary Materials

Supplementary Materials Figure S1: Cytotoxic effects caused by different PRV strains on different types of cells. Figure S2: Damages of main organs of fattening pigs caused by different PRV strains. Figure S3: Amino acid sequences alignments of main PRV glycoproteins (gB, gC, gD, gE, gG, gH, gL, gM, gN, and gK) between different PRV strains. Table S1: Reference PRV genome sequences used in this study. Table S2: Stable titers of PRV strains HeN21, HuB20, HBJZ-44-2021, and JSZL-2018 on PK-15 cells. Table S3: Comparisons of viral loads in different organs of pigs between different PRV-challenging groups. Table S4: Pathological injury scores of main organs of mice caused by different PRV strains. Table S5: Pathological injury scores of main organs of fattening pigs caused by different PRV strains.
