# Supplementary material for: Characterization of Pseudorabies Virus Associated with Severe Respiratory and Neuronal Signs in Old Pigs
Source: Transbound Emerg Dis. 2023 Feb 28;2023:8855739. doi: 10.1155/2023/8855739 (PMC12017139; doi:10.1155/2023/8855739)
Supplement: Supplementary Materials — Figure S1: Cytotoxic effects caused by different PRV strains on different types of cells. Figure S2: Damages of main organs of fattening pigs caused by different PRV strains. Figure S3: Amino acid sequences alignments of main PRV glycoproteins (gB, gC, gD, gE, gG, gH, gL, gM, gN, and gK) between different PRV strains. Table S1: Reference PRV genome sequences used in this study. Table S2: Stable titers of PRV strains HeN21, HuB20, HBJZ-44-2021, and JSZL-2018 on PK-15 cells. Table S3: Comparisons of viral loads in different organs of pigs between different PRV-challenging groups. Table S4: Pathological injury scores of main organs of mice caused by different PRV strains. Table S5: Pathological injury scores of main organs of fattening pigs caused by different PRV strains. [file 8855739.f1.zip › Figure S3.pdf]

gB

```
                           *        20         *        40         *        60         *
HeN21       : MPAGGGLWRGPRGHRPGHHGGAGLGRLWPAPHHAAAARGAVALALLLLALAATPTCGAAAVTRAASASPAPG :  72
HuB20       : MPAGGGLWRGPRGHRPGHHGGAGLGRLWPAPHHAAAARGAVALALLLLALAAAPPCGAAAVTRAASVSPTPV :  72
hSD-1       : MPAGGGLWRGPRGHRPGHHGGAGLGRLWPAPHHAAAARGAVALALLLLALAATPTCGAAAVTRAASASPAPG :  72
HeN1        : MPAGGGLWRGPRGHRPGHHGGAGLGRLWPAPHHAAAARGAVALALLLLALAATPTCGAAAVTRAASASPAPG :  72
HLJ8        : MPAGGGLWRGPRGHRPGHHGGAGLGRLWPAPHHAAAARGAVALALLLLALAATPTCGAAAVTRAASASPAPG :  72
HN1201      : MPAGGGLWRGPRGHRPGHHGGAGLGRLWPAPHHAAAARGAVALALLLLALAATPTCGAAAVTRAASASPAPG :  72
HNB         : MPAGGGLWRGPRGHRPGHHGGAGLGRLWPAPHHAAAARGAVALALLLLALAATPTCGAAAVTRAASASPAPG :  72
HNX         : MPAGGGLWRGPRGHRPGHHGGAGLGRLWPAPHHAAAARGAVALALLLLALAATPTCGAAAVTRAASASPAPG :  72
JS-2012     : MPAGGGLWRGPRGHRPGHHGGAGLGRLWPAPHHAAAARGAVALALLLLALAATPTCGAAAVTRAASASPAPG :  72
TJ          : MPAGGGLWRGPRGHRPGHHGGAGLGRLWPAPHHAAAARGAVALALLLLALAATPTCGAAAVTRAASASPAPG :  72
Ea          : MPAGGGLWRGPRGHRPGHHGGAGLGRLWPAPHHAAAARGAVALALLLLALAATPTCGAAAVTRAASASPAPG :  72
Fa          : MPAGGGLWRGPRGHRPGHHGGAGLGRLWPAPHHAAAARGAVALALLLLALAATPTCGAAAVTRAASASPAPG :  72
SC          : MPAGGGLWRGPRGHRPGHHGGAGLGRLWPAPHHAAAARGAVALALLLLALAATPTCGAAAVTRAASASPAPG :  72
Bartha-K61  : MPAGGGLWRGPRGHRPGHHGGAGLGRLWPAPHHAAAARGAVALALLLLALAAAPPCGAAAVTRAASASPTPV :  72
              MPAGGGLWRGPRGHRPGHHGGAGLGRLWPAPHHAAAARGAVALALLLLALAAtPtCGAAAVTRAASaSPaPg

                    80         *       100         *       120         *       140
HeN21       : TG---ATPDGFSAEESLEEIDGAVSPGPSDAPDGEYGDLDARTAVRAAATERDRFYVCPPPSGSTVVRLEPE : 141
HuB20       : PGSPGLTPNDVSAEESLEEIDGAVSPGPSDAPDGEYGDLDARTAVRAAATERDRFYVCPPPSGSTVVRLEPE : 144
hSD-1       : TG---ATPDGFSAEESLEEIDGAVSPGPSDAPDGEYGDLDARTAVRAAATERDRFYVCPPPSGSTVVRLEPE : 141
HeN1        : TG---ATPDGFSAEESLEEIDGAVSPGPSDAPDGEYGDLDARTAVRAAATERDRFYVCPPPSGSTVVRLEPE : 141
HLJ8        : TG---ATPDGFSAEESLEEIDGAVSPGPSDAPDGEYGDLDARTAVRAAATERDRFYVCPPPSGSTVVRLEPE : 141
HN1201      : TG---ATPDGFSAEESLEEIDGAVSPGPSDAPDGEYGDLDARTAVRAAATERDRFYVCPPPSGSTVVRLEPE : 141
HNB         : TG---ATPDGFSAEESLEEIDGAVSPGPSDAPDGEYGDLDARTAVRAAATERDRFYVCPPPSGSTVVRLEPE : 141
HNX         : TG---ATPDGFSAEESLEEIDGAVSPGPSDAPDGEYGDLDARTAVRAAATERDRFYVCPPPSGSTVVRLEPE : 141
JS-2012     : TG---ATPDGFSAEESLEEIDGAVSPGPSDAPDGEYGDLDARTAVRAAATERDRFYVCPPPSGSTVVRLEPE : 141
TJ          : TG---ATPDGFSAEESLEEIDGAVSPGPSDAPDGEYGDLDARTAVRAAATERDRFYVCPPPSGSTVVRLEPE : 141
Ea          : TG---ATPDGFSTEESLEEIDGAVSPGPSDAPDGEYGDLDARTAVRAAATERDRFYVCPPPSGSTVVRLEPE : 141
Fa          : TG---ATPDGFSTEESLEEIDGAVSPGPSDAPDGEYGDLDARTAVRAAATERDRFYVCPPPSGSTVVRLEPE : 141
SC          : TG---ATPDGFSTEESLEEIDGAVSPGPSDAPDGEYGDLDARTAVRAAATERDRFYVCPPPSGSTVVRLEPE : 141
Bartha-K61  : PGSPGLTPNDVSAEASLEEIE-AFTPGPSEAPDGEYGDLDARTAVRAAATERDRFYVCPPPSGSTVVRLEPE : 143
              tG   aTP1gfSaEeSLEEIdgAv3PGPSdAPDGEYGDLDARTAVRAAATERDRFYVCPPPSGSTVVRLEPE
```

```
                           *        160         *        180         *        200         *
HeN21      : QACPEYSQGRNFTEGIAVLFKENIAPHKFKAHIYYKNVIVTTVWSGSTYAAITNRFTDRVPVPVQEITDVID : 213
HuB20      : QACPEYSQGRNFTEGIAVLFKENIAPHKFKAHIYYKNVIVTTVWSGSTYAAITNRFTDRVPVPVQEITDVID : 216
hSD-1      : QACPEYSQGRNFTEGIAVLFKENIAPHKFKAHIYYKNVIVTTVWSGSTYAAITNRFTDRVPVPVQEITDVID : 213
HeN1       : QACPEYSQGRNFTEGIAVLFKENIAPHKFKAHIYYKNVIVTTVWSGSTYAAITNRFTDRVPVPVQEITDVID : 213
HLJ8       : QACPEYSQGRNFTEGIAVLFKENIAPHKFKAHIYYKNVIVTTVWSGSTYAAITNRFTDRVPVPVQEITDVID : 213
HN1201     : QACPEYSQGRNFTEGIAVLFKENIAPHKFKAHIYYKNVIVTTVWSGSTYAAITNRFTDRVPVPVQEITDVID : 213
HNB        : QACPEYSQGRNFTEGIAVLFKENIAPHKFKAHIYYKNVIVTTVWSGSTYAAITNRFTDRVPVPVQEITDVID : 213
HNX        : QACPEYSQGRNFTEGIAVLFKENIAPHKFKAHIYYKNVIVTTVWSGSTYAAITNRFTDRVPVPVQEITDVID : 213
JS-2012    : QACPEYSQGRNFTEGIAVLFKENIAPHKFKAHIYYKNVIVTTVWSGSTYAAITNRFTDRVPVPVQEITDVID : 213
TJ         : QACPEYSQGRNFTEGIAVLFKENIAPHKFKAHIYYKNVIVTTVWSGSTYAAITNRFTDRVPVPVQEITDVID : 213
Ea         : QACPEYSQGRNFTEGIAVLFKENIAPHKFKAHIYYKNVIVTTVWSGSTYAAITNRFTDRVPVPVQEITDVID : 213
Fa         : QACPEYSQGRNFTEGIAVLFKENIAPHKFKAHIYYKNVIVTTVWSGSTYAAITNRFTDRVPVPVQEITDVID : 213
SC         : QACPEYSQGRNFTEGIAVLFKENIAPHKFKAHIYYKNVIVTTVWSGSTYAAITNRFTDRVPVPVQEITDVID : 213
Bartha-K61 : QACPEYSQGRNFTEGIAVLFKENIAPHKFKAHIYYKNVIVTTVWSGSTYAAITNRFTDRVPVPVQEITDVID : 215
             QACPEYSQGRNFTEGIAVLFKENIAPHKFKAHIYYKNVIVTTVWSGSTYAAITNRFTDRVPVPVQEITDVID

              220         *        240         *        260         *        280
HeN21      : RRGKCVSKAEYVRNNHKVTAFDRDENPVEVDLRPSRLNALGTRGWHTTNDTYTKIGAAGFYHTGTSVNCIVE : 285
HuB20      : RRGKCVSKAEYVRNNHKVTAFDRDENPVEVDLRPSRLNALGTRGWHTTNDTYTKIGAAGFYHTGTSVNCIVE : 288
hSD-1      : RRGKCVSKAEYVRNNHKVTAFDRDENPVEVDLRPSRLNALGTRGWHTTNDTYTKIGAAGFYHTGTSVNCIVE : 285
HeN1       : RRGKCVSKAEYVRNNHKVTAFDRDENPVEVDLRPSRLNALGTRGWHTTNDTYTKIGAAGFYHTGTSVNCIVE : 285
HLJ8       : RRGKCVSKAEYVRNNHKVTAFDRDENPVEVDLRPSRLNALGTRGWHTTNDTYTKIGAAGFYHTGTSVNCIVE : 285
HN1201     : RRGKCVSKAEYVRNNHKVTAFDRDENPVEVDLRPSRLNALGTRGWHTTNDTYTKIGAAGFYHTGTSVNCIVE : 285
HNB        : RRGKCVSKAEYVRNNHKVTAFDRDENPVEVDLRPSRLNALGTRGWHTTNDTYTKIGAAGFYHTGTSVNCIVE : 285
HNX        : RRGKCVSKAEYVRNNHKVTAFDRDENPVEVDLRPSRLNALGTRGWHTTNDTYTKIGAAGFYHTGTSVNCIVE : 285
JS-2012    : RRGKCVSKAEYVRNNHKVTAFDRDENPVEVDLRPSRLNALGTRGWHTTNDTYTKIGAAGFYHTGTSVNCIVE : 285
TJ         : RRGKCVSKAEYVRNNHKVTAFDRDENPVEVDLRPSRLNALGTRGWHTTNDTYTKIGAAGFYHTGTSVNCIVE : 285
Ea         : RRGKCVSKAEYVRNNHKVTAFDRDENPVEVDLRPSRLNALGTRGWHTTNDTYTKIGAAGFYHTGTSVNCIVE : 285
Fa         : RRGKCVSKAEYVRNNHKVTAFDRDENPVEVDLRPSRLNALGTRGWHTTNDTYTKIGAAGFYHTGTSVNCIVE : 285
SC         : RRGKCVSKAEYVRNNHKVTAFDRDENPVEVDLRPSRLNALGTRGWHTTNDTYTKIGAAGFYHTGTSVNCIVE : 285
Bartha-K61 : RRGKCVSKAEYVRNNHKVTAFDRDENPVEVDLRPSRLNALGTRGWHTTNDTHTKIGAAGFYHTGTSVNCIVE : 287
             RRGKCVSKAEYVRNNHKVTAFDRDENPVEVDLRPSRLNALGTRGWHTTNDTyTKIGAAGFYHTGTSVNCIVE
```

```
                    *       300         *       320         *       340         *       360
HeN21       : EVEARSVYPYDSFALSTGDIVYMSPFYGLREGAHGEHIGYAPGRFQQVEHYYPIDLDSRLRASESVTRNFLR : 357
HuB20       : EVEARSVYPYDSFALSTGDIVYMSPFYGLREGAHGEHIGYAPGRFQQVEHYYPIDLDSRLRASESVTRNFLR : 360
hSD-1       : EVEARSVYPYDSFALSTGDIVYMSPFYGLREGAHGEHIGYAPGRFQQVEHYYPIDLDSRLRASESVTRNFLR : 357
HeN1        : EVEARSVYPYDSFALSTGDIVYMSPFYGLREGAHGEHIGYAPGRFQQVEHYYPIDLDSRLRASESVTRNFLR : 357
HLJ8        : EVEARSVYPYDSFALSTGDIVYMSPFYGLREGAHGEHIGYAPGRFQQVEHYYPIDLDSRLRASESVTRNFLR : 357
HN1201      : EVEARSVYPYDSFALSTGDIVYMSPFYGLREGAHGEHIGYAPGRFQQVEHYYPIDLDSRLRASESVTRNFLR : 357
HNB         : EVEARSVYPYDSFALSTGDIVYMSPFYGLREGAHGEHIGYAPGRFQQVEHYYPIDLDSRLRASESVTRNFLR : 357
HNX         : EVEARSVYPYDSFALSTGDIVYMSPFYGLREGAHGEHIGYAPGRFQQVEHYYPIDLDSRLRASESVTRNFLR : 357
JS-2012     : EVEARSVYPYDSFALSTGDIVYMSPFYGLREGAHGEHIGYAPGRFQQVEHYYPIDLDSRLRASESVTRNFLR : 357
TJ          : EVEARSVYPYDSFALSTGDIVYMSPFYGLREGAHGEHIGYAPGRFQQVEHYYPIDLDSRLRASESVTRNFLR : 357
Ea          : EVEARSVYPYDSFALSTGDIVYMSPFYGLREGAHGEHIGYAPGRFQQVEHYYPIDLDSRLRASESVTRNFLR : 357
Fa          : EVEARSVYPYDSFALSTGDIVYMSPFYGLREGAHGEHIGYAPGRFQQVEHYYPIDLDSRLRASESVTRNFLR : 357
SC          : EVEARSVYPYDSFALSTGDIVYMSPFYGLREGAHGEHIGYAPGRFQQVEHYYPIDLDSRLRASESVTRNFLR : 357
Bartha-K61  : EVEARSVYPYDSFALSTGDIVYMSPFYGLREGAHGEHIGYAPGRFQQVEHYYPIDLDSRLRASESVTRNFLR : 359
              EVEARSVYPYDSFALSTGDIVYMSPFYGLREGAHGEHIGYAPGRFQQVEHYYPIDLDSRLRASESVTRNFLR

                      *       380         *       400         *       420         *
HeN21       : TPHFTVAWDWAPKTRRVCSLAKWREAEEMIRDETRDGSFRFTSRALGASFVSDVTQLDLQRVHLGDCVLREA : 429
HuB20       : TPHFTVAWDWAPKTRRVCSLAKWREAEEMIRDETRDGSFRFTSRALGASFVSDVTQLDLQRVHLGDCVLREA : 432
hSD-1       : TPHFTVAWDWAPKTRRVCSLAKWREAEEMIRDETRDGSFRFTSRALGASFVSDVTQLDLQRVHLGDCVLREA : 429
HeN1        : TPHFTVAWDWAPKTRRVCSLAKWREAEEMIRDETRDGSFRFTSRALGASFVSDVTQLDLQRVHLGDCVLREA : 429
HLJ8        : TPHFTVAWDWAPKTRRVCSLAKWREAEEMIRDETRDGSFRFTSRALGASFVSDVTQLDLQRVHLGDCVLREA : 429
HN1201      : TPHFTVAWDWAPKTRRVCSLAKWREAEEMIRDETRDGSFRFTSRALGASFVSDVTQLDLQRVHLGDCVLREA : 429
HNB         : TPHFTVAWDWAPKTRRVCSLAKWREAEEMIRDETRDGSFRFTSRALGASFVSDVTQLDLQRVHLGDCVLREA : 429
HNX         : TPHFTVAWDWAPKTRRVCSLAKWREAEEMIRDETRDGSFRFTSRALGASFVSDVTQLDLQRVHLGDCVLREA : 429
JS-2012     : TPHFTVAWDWAPKTRRVCSLAKWREAEEMIRDETRDGSFRFTSRALGASFVSDVTQLDLQRVHLGDCVLREA : 429
TJ          : TPHFTVAWDWAPKTRRVCSLAKWREAEEMIRDETRDGSFRFTSRALGASFVSDVTQLDLQRVHLGDCVLREA : 429
Ea          : TPHFTVAWDWAPKTRRVCSLAKWREAEEMIRDETRDGSFRFTSRALGASFVSDVTQLDLQRVHLGDCVLREA : 429
Fa          : TPHFTVAWDWAPKTRRVCSLAKWREAEEMIRDETRDGSFRFTSRALGASFVSDVTQLDLQRVHLGDCVLREA : 429
SC          : TPHFTVAWDWAPKTRRVCSLAKWREAEEMIRDETRDGSFRFTSRALGASFVSDVTQLDLQRVHLGDCVLREA : 429
Bartha-K61  : TPHFTVAWDWAPKTRRVCSLAKWREAEEMIRDETRDGSFRFTSRALGASFVSDVTQLDLQRVHLGDCVLREA : 431
              TPHFTVAWDWAPKTRRVCSLAKWREAEEMIRDETRDGSFRFTSRALGASFVSDVTQLDLQRVHLGDCVLREA
```

```
                  440         *        460         *        480         *        500
HeN21      : SEAIDAIYRRRYNNTHVLAGDKPEVYLARGGFVVAFRPLISNELAQLYARELERLGLAGVVGPASPAAARRA : 501
HuB20      : SEAIDAIYRRRYNNTHVLAGEKPEVYLARGGFVVAFRPLISNELAQLYARELERLGLAGVVGPASPAAARRT : 504
hSD-1      : SEAIDAIYRRRYNNTHVLAGDKPEVYLARGGFVVAFRPLISNELAQLYARELERLGLAGVVGPASPAAARRA : 501
HeN1       : SEAIDAIYRRRYNNTHVLAGDKPEVYLARGGFVVAFRPLISNELAQLYARELERLGLAGVVGPASPAAARRA : 501
HLJ8       : SEAIDAIYRRRYNNTHVLAGDKPEVYLARGGFVVAFRPLISNELAQLYARELERLGLAGVVGPASPAAARRA : 501
HN1201     : SEAIDAIYRRRYNNTHVLAGDKPEVYLARGGFVVAFRPLISNELAQLYARELERLGLAGVVGPASPAAARRA : 501
HNB        : SEAIDAIYRRRYNNTHVLAGDKPEVYLARGGFVVAFRPLISNELAQLYARELERLGLAGVVGPASPAAARRA : 501
HNX        : SEAIDAIYRRRYNNTHVLAGDKPEVYLARGGFVVAFRPLISNELAQLYARELERLGLAGVVGPASPAAARRA : 501
JS-2012    : SEAIDAIYRRRYNNTHVLAGDKPEVYLARGGFVVAFRPLISNELAQLYARELERLGLAGVVGPASPAAARRA : 501
TJ         : SEAIDAIYRRRYNNTHVLAGDKPEVYLARGGFVVAFRPLISNELAQLYARELERLGLAGVVGPASPAAARRA : 501
Ea         : SEAIDAIYRRRYNNTHVLAGDRPEVYLARGGFVVAFRPLISNELAQLYARELERLGLAGVVGPASPAAARRA : 501
Fa         : SEAIDAIYRRRYNNTHVLAGDRPEVYLARGGFVVAFRPLISNELAQLYARELERLGLAGVVGPASPAAARRA : 501
SC         : SEAIDAIYRRRYNNTHVLAGDRPEVYLARGGFVVAFRPLISNELAQLYARELERLGLAGVVGPASPAAARRA : 501
Bartha-K61 : SEAIDAIYQRRYNNTHVLAGDRPEVYLARGGFVVAFRPLISNELAQLYARELERLGLAGVVGPASPAAARRA : 503
             SEAIDAIYrRRYNNTHVLAGd4PEVYLARGGFVVAFRPLISNELAQLYARELERLGLAGVVGPASPAAARRa

                  *        520         *        540         *        560         *
HeN21      : RRSPGPAGTPEPPAVNGTGHLRITTGSAEFARLQFTYDHIQAHVNDMLSRIAAAWCELQNKDRTLWGEMSRL : 573
HuB20      : RRSPGQAGTPEPPAVNGTGHLRITTGSAEFARLQFTYDHIQAHVNDMLSRIAAAWCELQNKDRTLWGEMSRL : 576
hSD-1      : RRSPGPAGTPEPPAVNGTGHLRITTGSAEFARLQFTYDHIQAHVNDMLSRIAAAWCELQNKDRTLWGEMSRL : 573
HeN1       : RRSPGPAGTPEPPAVNGTGHLRITTGSAEFARLQFTYDHIQAHVNDMLSRIAAAWCELQNKDRTLWGEMSRL : 573
HLJ8       : RRSPGPAGTPEPPAVNGTGHLRITTGSAEFARLQFTYDHIQAHVNDMLSRIAAAWCELQNKDRTLWGEMSRL : 573
HN1201     : RRSPGPAGTPEPPAVNGTGHLRITTGSAEFARLQFTYDHIQAHVNDMLSRIAAAWCELQNKDRTLWGEMSRL : 573
HNB        : RRSPGPAGTPEPPAVNGTGHLRITTGSAEFARLQFTYDHIQAHVNDMLSRIAAAWCELQNKDRTLWGEMSRL : 573
HNX        : RRSPGPAGTPEPPAVNGTGHLRITTGSAEFARLQFTYDHIQAHVNDMLSRIAAAWCELQNKDRTLWGEMSRL : 573
JS-2012    : RRSPGPAGTPEPPAVNGTGHLRITTGSAEFARLQFTYDHIQAHVNDMLSRIAAAWCELQNKDRTLWGEMSRL : 573
TJ         : RRSPGPAGTPEPPAVNGTGHLRITTGSAEFARLQFTYDHIQAHVNDMLSRIAAAWCELQNKDRTLWGEMSRL : 573
Ea         : RRSPGPAGTPEPPAVNGTGHLRITTGSAEFARLQFTYDHIQAHVNDMLSRIAAAWCELHNKDRTLWGEMSRL : 573
Fa         : RRSPGPAGTPEPPAVNGTGHLRITTGSAEFARLQFTYDHIQAHVNDMLSRIAAAWCELHNKDRTLWGEMSRL : 573
SC         : RRSPGPAGTPEPPAVNGTGHLRITTGSAEFARLQFTYDHIQAHVNDMLSRIAAAWCELHNKDRTLWGEMSRL : 573
Bartha-K61 : RRAAGQAGTPEPPAVNGTGHLRITTGSAEFARLQFTYDHIQAHVNDMLGRIAAAWCELQNKDRTLWSEMSRL : 575
             RRspGpAGTPEPPAVNGTGHLRITTGSAEFARLQFTYDHIQAHVNDMLsRIAAAWCELqNKDRTLWgEMSRL
```

```
                 580         *       600         *       620         *       640
HeN21       : NPSAVATAALGQRVSARMLGDVMAISRCVEVRGGVYVQNSMRVPGERGTCYSRPLVTFEHNGTGVIEGQLGD : 645
HuB20       : NPSAVATAALGQRVSARMLGDVMAISRCVEVRGGVYVQNSMRVPGERGTCYSRPLVTFEHNGTGVIEGQLGD : 648
hSD-1       : NPSAVATAALGQRVSARMLGDVMAISRCVEVRGGVYVQNSMRVPGERGTCYSRPLVTFEHNGTGVIEGQLGD : 645
HeN1        : NPSAVATAALGQRVSARMLGDVMAISRCVEVRGGVYVQNSMRVPGERGTCYSRPLVTFEHNGTGVIEGQLGD : 645
HLJ8        : NPSAVATAALGQRVSARMLGDVMAISRCVEVRGGVYVQNSMRVPGERGTCYSRPLVTFEHNGTGVIEGQLGD : 645
HN1201      : NPSAVATAALGQRVSARMLGDVMAISRCVEVRGGVYVQNSMRVPGERGTCYSRPLVTFEHNGTGVIEGQLGD : 645
HNB         : NPSAVATAALGQRVSARMLGDVMAISRCVEVRGGVYVQNSMRVPGERGTCYSRPLVTFEHNGTGVIEGQLGD : 645
HNX         : NPSAVATAALGQRVSARMLGDVMAISRCVEVRGGVYVQNSMRVPGERGTCYSRPLVTFEHNGTGVIEGQLGD : 645
JS-2012     : NPSAVATAALGQRVSARMLGDVMAISRCVEVRGGVYVQNSMRVPGERGTCYSRPLVTFEHNGTGVIEGQLGD : 645
TJ          : NPSAVATAALGQRVSARMLGDVMAISRCVEVRGGVYVQNSMRVPGERGTCYSRPLVTFEHNGTGVIEGQLGD : 645
Ea          : NPSAVATAALGQRVSARMLGDVMAISRCVEVRGGVYVQNSMRVPGERGTCYSRPLVTFEHNGTGVIEGQLGD : 645
Fa          : NPSAVATAALGQRVSARMLGDVMAISRCVEVRGGVYVQNSMRVPGERGTCYSRPLVTFEHNGTGVIEGQLGD : 645
SC          : NPSAVATAALGQRVSARMLGDVMAISRCVEVRGGVYVQNSMRVPGERGTCYSRPLVTFEHNGTGVIEGQLGD : 645
Bartha-K61  : NPSAVATAALGQRVSARMLGDVMAISRCVEVRGGVYVQNSMRVPGERGTCYSRPLVTFEHNGTGVIEGQLGD : 647
              NPSAVATAALGQRVSARMLGDVMAISRCVEVRGGVYVQNSMRVPGERGTCYSRPLVTFEHNGTGVIEGQLGD

               *       660         *       680         *       700         *       720
HeN21       : DNELLISRDLIEPCTGNHRRYFKLGGGYVYYEDYSYVRMVEVPETISTRVTLNLTLLEDREFLPLEVYTREE : 717
HuB20       : DNELLISRDLIEPCTGNHRRYFKLGGGYVYYEDYSYVRMVEVPETISTRVTLNLTLLEDREFLPLEVYTREE : 720
hSD-1       : DNELLISRDLIEPCTGNHRRYFKLGGGYVYYEDYSYVRMVEVPETISTRVTLNLTLLEDREFLPLEVYTREE : 717
HeN1        : DNELLISRDLIEPCTGNHRRYFKLGGGYVYYEDYSYVRMVEVPETISTRVTLNLTLLEDREFLPLEVYTREE : 717
HLJ8        : DNELLISRDLIEPCTGNHRRYFKLGGGYVYYEDYSYVRMVEVPETISTRVTLNLTLLEDREFLPLEVYTREE : 717
HN1201      : DNELLISRDLIEPCTGNHRRYFKLGGGYVYYEDYSYVRMVEVPETISTRVTLNLTLLEDREFLPLEVYTREE : 717
HNB         : DNELLISRDLIEPCTGNHRRYFKLGGGYVYYEDYSYVRMVEVPETISTRVTLNLTLLEDREFLPLEVYTREE : 717
HNX         : DNELLISRDLIEPCTGNHRRYFKLGGGYVYYEDYSYVRMVEVPETISTRVTLNLTLLEDREFLPLEVYTREE : 717
JS-2012     : DNELLISRDLIEPCTGNHRRYFKLGGGYVYYEDYSYVRMVEVPETISTRVTLNLTLLEDREFLPLEVYTREE : 717
TJ          : DNELLISRDLIEPCTGNHRRYFKLGGGYVYYEDYSYVRMVEVPETISTRVTLNLTLLEDREFLPLEVYTREE : 717
Ea          : DNELLISRDLIEPCTGNHRRYFKLGGGYVYYEDYSYVRMVEVPETISTRVTLNLTLLEDREFLPLEVYTREE : 717
Fa          : DNELLISRDLIEPCTGNHRRYFKLGGGYVYYEDYSYVRMVEVPETISTRVTLNLTLLEDREFLPLEVYTREE : 717
SC          : DNELLISRDLIEPCTGNHRRYFKLGGGYVYYEDYSYVRMVEVPETISTRVTLNLTLLEDREFLPLEVYTREE : 717
Bartha-K61  : DNELLISRDLIEPCTGNHRRYFKLGSGYVYYEDYSYVRMVEVPETISTRVTLNLTLLEDREFLPLEVYTREE : 719
              DNELLISRDLIEPCTGNHRRYFKLGgGYVYYEDYSYVRMVEVPETISTRVTLNLTLLEDREFLPLEVYTREE
```

```
                      *       740         *       760         *       780         *
HeN21      : LADTGLLDYSEIQRRNQLHALKFYDIDRVVKVDHNVVLLRGIANFFQGLGDVGAAVGKVVLGATGAVISAVG : 789
HuB20      : LADTGLLDYSEIQRRNQLHALKFYDIDRVVKVDHNVVLLRGIANFFQGLGDVGAAVGKVVLGATGAVISAVG : 792
hSD-1      : LADTGLLDYSEIQRRNQLHALKFYDIDRVVKVDHNVVLLRGIANFFQGLGDVGAAVGKVVLGATGAVISAVG : 789
HeN1       : LADTGLLDYSEIQRRNQLHALKFYDIDRVVKVDHNVVLLRGIANFFQGLGDVGAAVGKVVLGATGAVISAVG : 789
HLJ8       : LADTGLLDYSEIQRRNQLHALKFYDIDRVVKVDHNVVLLRGIANFFQGLGDVGAAVGKVVLGATGAVISAVG : 789
HN1201     : LADTGLLDYSEIQRRNQLHALKFYDIDRVVKVDHNVVLLRGIANFFQGLGDVGAAVGKVVLGATGAVISAVG : 789
HNB        : LADTGLLDYSEIQRRNQLHALKFYDIDRVVKVDHNVVLLRGIANFFQGLGDVGAAVGKVVLGATGAVISAVG : 789
HNX        : LADTGLLDYSEIQRRNQLHALKFYDIDRVVKVDHNVVLLRGIANFFQGLGDVGAAVGKVVLGATGAVISAVG : 789
JS-2012    : LADTGLLDYSEIQRRNQLHALKFYDIDRVVKVDHNVVLLRGIANFFQGLGDVGAAVGKVVLGATGAVISAVG : 789
TJ         : LADTGLLDYSEIQRRNQLHALKFYDIDRVVKVDHNVVLLRGIANFFQGLGDVGAAVGKVVLGATGAVISAVG : 789
Ea         : LADTGLLDYSEIQRRNQLHTLKFYDIDRVVKVDHNVVLLRGIANFFQGLGDVGAAVGKVVLGATGAVISAVG : 789
Fa         : LADTGLLDYSEIQRRNQLHTLKFYDIDRVVKVDHNVVLLRGIANFFQGLGDVGAAVGKVVLGATGAVISAVG : 789
SC         : LADTGLLDYSEIQRRNQLHTLKFYDIDRVVKVDHNVVLLRGIANFFQGLGDVGAAVGKVVLGATGAVISAVG : 789
Bartha-K61 : LADTGLLDYSEIQRRNQLHALKFYDIDRVVKVDHNVVLLRGIANFFQGLGDVGAAVGKVVLGATGAVISAVG : 791
             LADTGLLDYSEIQRRNQLHaLKFYDIDRVVKVDHNVVLLRGIANFFQGLGDVGAAVGKVVLGATGAVISAVG

                  800         *       820         *       840         *       860
HeN21      : GMVSFLSNPFGALAIGLLVLAGLVAAFLAYRHISRLRRNPMKALYPVTTKALKEDGVEEDDVDEAKLDQARD : 861
HuB20      : GMVSFLSNPFGALAIGLLVLAGLVAAFLAYRHISRLRRNPMKALYPVTTKALKEDGVEEGDVDEAKLDQARD : 864
hSD-1      : GMVSFLSNPFGALAIGLLVLAGLVAAFLAYRHISRLRRNPMKALYPVTTKALKEDGVEEDDVDEAKLDQARD : 861
HeN1       : GMVSFLSNPFGALAIGLLVLAGLVAAFLAYRHISRLRRNPMKALYPVTTKALKEDGVEEDDVDEAKLDQARD : 861
HLJ8       : GMVSFLSNPFGALAIGLLVLAGLVAAFLAYRHISRLRRNPMKALYPVTTKALKEDGVEEDDVDEAKLDQARD : 861
HN1201     : GMVSFLSNPFGALAIGLLVLAGLVAAFLAYRHISRLRRNPMKALYPVTTKALKEDGVEEDDVDEAKLDQARD : 861
HNB        : GMVSFLSNPFGALAIGLLVLAGLVAAFLAYRHISRLRRNPMKALYPVTTKALKEDGVEEDDVDEAKLDQARD : 861
HNX        : GMVSFLSNPFGALAIGLLVLAGLVAAFLAYRHISRLRRNPMKALYPVTTKALKEDGVEEDDVDEAKLDQARD : 861
JS-2012    : GMVSFLSNPFGALAIGLLVLAGLVAAFLAYRHISRLRRNPMKALYPVTTKALKEDGVEEDDVDEAKLDQARD : 861
TJ         : GMVSFLSNPFGALAIGLLVLAGLVAAFLAYRHISRLRRNPMKALYPVTTKALKEDGVEEDDVDEAKLDQARD : 861
Ea         : GMVSFLSNPFGALAIGLLVLAGLVAAFLAYRHISRLRRNPMKALYPVTTKALKEDGVEEDDVDEAKLDQARD : 861
Fa         : GMVSFLSNPFGALAIGLLVLAGLVAAFLAYRHISRLRRNPMKALYPVTTKALKEDGVEEDDVDEAKLDQARD : 861
SC         : GMVSFLSNPFGALAIGLLVLAGLVAAFLAYRHISRLRRNPMKALYPVTTKALKEDGVEEDDVDEAKLDQARD : 861
Bartha-K61 : GMVSFLSNPFGALAIGLLVLAGLVAAFLAYRHISRLRRNPMKALYPVTTKTLKEDGVDEGDVDEAKLDQARD : 863
             GMVSFLSNPFGALAIGLLVLAGLVAAFLAYRHISRLRRNPMKALYPVTTKaLKEDGVeEdDVDEAKLDQARD
```

```
                   *        880         *        900         *
HeN21      : MIRYMSIVSALEQQEHKARKKNSGPALLASRVGAMATRRRHYQRLENEDPDAP* : 914
HuB20      : MIRYMSIVSALEQQEHKARKKNSGPALLASRVGAMATRRRHYQRLENEDPDAP* : 917
hSD-1      : MIRYMSIVSALEQQEHKARKKNSGPALLASRVGAMATRRRHYQRLENEDPDAP- : 914
HeN1       : MIRYMSIVSALEQQEHKARKKNSGPALLASRVGAMATRRRHYQRLENEDPDAP- : 914
HLJ8       : MIRYMSIVSALEQQEHKARKKNSGPALLASRVGAMATRRRHYQRLENEDPDAP- : 914
HN1201     : MIRYMSIVSALEQQEHKARKKNSGPALLASRVGAMATRRRHYQRLENEDPDAP- : 914
HNB        : MIRYMSIVSALEQQEHKARKKNSGPALLASRVGAMATRRRHYQRLENEDPDAP- : 914
HNX        : MIRYMSIVSALEQQEHKARKKNSGPALLASRVGAMATRRRHYQRLENEDPDAP- : 914
JS-2012    : MIRYMSIVSALEQQEHKARKKNSGPALLASRVGAMATRRRHYQRLENEDPDAP- : 914
TJ         : MIRYMSIVSALEQQEHKARKKNSGPALLASRVGAMATRRRHYQRLENEDPDAP- : 914
Ea         : MIRYMSIVSALEQQEHKARKKNSGPALLASRVGVMATRRRHYQRLENEDPDAP- : 914
Fa         : MIRYMSIVSALEQQEHKARKKNSGPALLASRVGVMATRRRHYQRLENEDPDAP- : 914
SC         : MIRYMSIVSALEQQEHKARKKNSGPALLASRVGVMATRRRHYQRLENEDPDAP- : 914
Bartha-K61 : MIRYMSIVSALEQQEHKARKKNSGPALLASRVGAMATRRRHYQRLESEDPDAL* : 916
             MIRYMSIVSALEQQEHKARKKNSGPALLASRVGaMATRRRHYQRLEnEDPDAp
```

gC

```
                           *        20         *        40         *        60         *
HeN21      : MASLARAMLALLALYTAAIAAAPSSTTALGTTPNGGGGGNSSAGELSPSPPSTPEPVSGTTGAAASTPAAVS :  72
HuB20      : MASLARAMLALLALYTAAIAAAPSTTTALGTTPNGGGGGNSSAGELSPSPPSTPEPVSGTPGAAASTPAAVS :  72
hSD-1      : MASLARAMLALLALYTAAIAAAPSSTTALGTTPNGGGGGNSSAGELSPSPPSTPEPVSGTTGAAASTPAAVS :  72
HeN1       : MASLARAMLALLALYTAAIAAAPSSTTALGTTPNGGGGGNSSAGELSPSPPSTPEPVSGTTGAAASTPAAVS :  72
HLJ8       : MASLARAMLALLALYTAAIAAAPSSTTALGTTPNGGGGGNSSAGELSPSPPSTPEPVSGTTGAAASTPAAVS :  72
HN1201     : MASLARAMLALLALYTAAIAAAPSSTTALGTTPNGGGGGNSSAGELSPSPPSTPEPVSGTTGAAASTPAAVS :  72
HNB        : MASLARAMLALLALYTAAIAAAPSSTTALGTTPNGGGGGNSSAGELSPSPPSTPEPVSGTTGAAASTPAAVS :  72
HNX        : MASLARAMLALLALYTAAIAAAPSSTTALGTTPNGGGGGNSSAGELSPSPPSTPEPVSGTTGAAASTPAAVS :  72
JS-2012    : MASLARAMLALLALYTAAIAAAPSSTTALGTTPNGGGGGNSSAGELSPSPPSTPEPVSGTTGAAASTPAAVS :  72
TJ         : MASLARAMLALLALYTAAIAAAPSSTTALGTTPNGGGGGNSSAGELSPSPPSTPEPVSGTTGAAASTPAAVS :  72
Ea         : MASLARAMLALLALYTAAIAAAPSSTTALGTTPTGGGGGNSSAGELSPSPPSTPEPVSGTTGAAASTPAAVS :  72
Fa         : MASLARAMLALLALYTAAIAAAPSSTTALGTTPTGGGGGNSSAGELSPSPPSTPEPVSGTTGAAASTPAAVS :  72
SC         : MASLARAMLALLAPYAAAIAAAPSTTTALGTTPNGGGGGNSSAGELSPSPPPTPAPASPEAG-------AVS :  65
Bartha-K61 : MASLARAMLALLAPYAAAIAAAPSTTTALGTTPNGGGGGNSSAGELSPSPPPTPAPASPEAG-------AVS :  65
             MASLARAMLALLAlYtAAIAAAPS3TTALGTTPnGGGGGNSSAGELSPSPPsTPePvSgttGaaastpaAVS

                  80         *       100         *       120         *       140
HeN21      : TPRVPPPSVSRRKPQRNGNRTRVHGDKATSHGRKRIVCRERLFSARVGDAVSFGCAVVPRAGETFEVRFCRR : 144
HuB20      : TPPVPPPSVSRRKPQRNGNRTRVHGDKATSHGRKRIVCRERLFSARVGDAVSFGCAVVPRAGETFEVRFYRR : 144
hSD-1      : TPRVPPPSVSRRKPQRNGNRTRVHGDKATSHGRKRIVCRERLFSARVGDAVSFGCAVVPRAGETFEVRFCRR : 144
HeN1       : TPRVPPPSVSRRKPQRNGNRTRVHGDKATSHGRKRIVCRERLFSARVGDAVSFGCAVVPRAGETFEVRFCRR : 144
HLJ8       : TPRVPPPSVSRRKPQRNGNRTRVHGDKATSHGRKRIVCRERLFSARVGDAVSFGCAVVPRAGETFEVRFCRR : 144
HN1201     : TPRVPPPSVSRRKPQRNGNRTRVHGDKATSHGRKRIVCRERLFSARVGDAVSFGCAVVPRAGETFEVRFCRR : 144
HNB        : TPRVPPPSVSRRKPQRNGNRTRVHGDKATSHGRKRIVCRERLFSARVGDAVSFGCAVVPRAGETFEVRFCRR : 144
HNX        : TPRVPPPSVSRRKPQRNGNRTRVHGDKATSHGRKRIVCRERLFSARVGDAVSFGCAVVPRAGETFEVRFCRR : 144
JS-2012    : TPRVPPPSVSRRKPQRNGNRTRVHGDKATSHGRKRIVCRERLFSARVGDAVSFGCAVVPRAGETFEVRFCRR : 144
TJ         : TPRVPPPSVSRRKPQRNGNRTRVHGDKATSHGRKRIVCRERLFSARVGDAVSFGCAVVPRAGETFEVRFCRR : 144
Ea         : TPRVPPPSVSRRKPQRNGNRTRVHGDEATSHGRKRIVCRERLFSARVGDAVSFGCAVVPRAGETFEVRFCRR : 144
Fa         : TPRVPPPSVSRRKPQRNGNRTRVHGDEATSHGRKRIVCRERLFSARVGDAVSFGCAVVPRAGETFEVRFCRR : 144
SC         : TPRAPPPSVSRRKPPRNNNRTRVHGDKATAHGRERIVCRERLFSARVGDAVSFGCAVFPRAGETFEVRFYRR : 137
Bartha-K61 : TPRAPPPSVSRRKPPRNNNRTRVHGDKATAHGRKRIVCRERLFSARVGDAVSFGCAVFPRAGETFEVRFYRR : 137
             TPrvPPPSVSRRKPqRNgNRTRVHGDkATsHGRkRIVCRERLFSARVGDAVSFGCAVvPRAGETFEVRFcRR
```

```
                       *       160         *       180         *       200         *
HeN21          : GRFRSPDADPEYFDEPPRPELPRERLLFSSANASLAHADALASAVVVEGERATVANVSGEVSVRVAAADAET : 216
HuB20          : GRFRSPDADPEYFDEPPRPELPRERLLFSSANASLAHADALAPAVVVEGERATVANVSGEVSVRVTAADAET : 216
hSD-1          : GRFRSPDADPEYFDEPPRPELPRERLLFSSANASLAHADALASAVVVEGERATVANVSGEVSVRVAAADAET : 216
HeN1           : GRFRSPDADPEYFDEPPRPELPRERLLFSSANASLAHADALASAVVVEGERATVANVSGEVSVRVAAADAET : 216
HLJ8           : GRFRSPDADPEYFDEPPRPELPRERLLFSSANASLAHADALASAVVVEGERATVANVSGEVSVRVAAADAET : 216
HN1201         : GRFRSPDADPEYFDEPPRPELPRERLLFSSANASLAHADALASAVVVEGERATVANVSGEVSVRVAAADAET : 216
HNB            : GRFRSPDADPEYFDEPPRPELPRERLLFSSANASLAHADALASAVVVEGERATVANVSGEVSVRVAAADAET : 216
HNX            : GRFRSPDADPEYFDEPPRPELPRERLLFSSANASLAHADALASAVVVEGERATVANVSGEVSVRVAAADAET : 216
JS-2012        : GRFRSPDADPEYFDEPPRPELPRERLLFSSANASLAHADALASAVVVEGERATVANVSGEVSVRVAAADAET : 216
TJ             : GRFRSPDADPEYFDEPPRPELPRERLLFSSANASLAHADALASAVVVEGERATVANVSGEVSVRVAAADAET : 216
Ea             : GRFRSPDADPEYFDEPPRPELPRERLLFSSANASLAHADALASAVVVEGGRATVANVSGEVSVRVAAADAET : 216
Fa             : GRFRSPDADPEYFDEPPRPELPRERLLFSSANASLAHADALASAVVVEGGRATVANVSGEVSVRVAAADAET : 216
SC             : GRFRSPDADPEYFDEPPRSELPRERLLFSSANASLAHADALTPVVEDEGGRATVANVSGEVSVRVAAADAET : 209
Bartha-K61     : GRFRSPDADPEYFDEPPRSELPRERLLFSSANASLAHADALTPVVEDEGGRATVANVSGEVSVRVAAADAET : 209
                 GRFRSPDADPEYFDEPPRpELPRERLLFSSANASLAHADALasaVvvEG RATVANVSGEVSVRVaAADAET

                  220         *       240         *       260         *       280
HeN21          : EGVYTWRVLSANGTEVRSANVSLVLYHQPEFGLSAPPVLFGEPFRAVCVVRDYYPRRSVRLRWFADEHPVDA : 288
HuB20          : EGVYTWRVLSTNGTEVRSANVSLVLYHQPEFGLSAPPVLFGEPFRAVCVVRDYYPRRSVRLRWFADEHPVDA : 288
hSD-1          : EGVYTWRVLSANGTEVRSANVSLVLYHQPEFGLSAPPVLFGEPFRAVCVVRDYYPRRSVRLRWFADEHPVDA : 288
HeN1           : EGVYTWRVLSANGTEVRSANVSLVLYHQPEFGLSAPPVLFGEPFRAVCVVRDYYPRRSVRLRWFADEHPVDA : 288
HLJ8           : EGVYTWRVLSANGTEVRSANVSLVLYHQPEFGLSAPPVLFGEPFRAVCVVRDYYPRRSVRLRWFADEHPVDA : 288
HN1201         : EGVYTWRVLSANGTEVRSANVSLVLYHQPEFGLSAPPVLFGEPFRAVCVVRDYYPRRSVRLRWFADEHPVDA : 288
HNB            : EGVYTWRVLSANGTEVRSANVSLVLYHQPEFGLSAPPVLFGEPFRAVCVVRDYYPRRSVRLRWFADEHPVDA : 288
HNX            : EGVYTWRVLSANGTEVRSANVSLVLYHQPEFGLSAPPVLFGEPFRAVCVVRDYYPRRSVRLRWFADEHPVDA : 288
JS-2012        : EGVYTWRVLSANGTEVRSANVSLVLYHQPEFGLSAPPVLFGEPFRAVCVVRDYYPRRSVRLRWFADEHPVDA : 288
TJ             : EGVYTWRVLSANGTEVRSANVSLVLYHQPEFGLSAPPVLFGEPFRAVCVVRDYYPRRSVRLRWFADEHPVDA : 288
Ea             : EGVYTWRVLSANGTEVSSANVSLVLYHQPEFGLSAPPVLFGEPFRAVCVVRDYYPRRSVRLRWFADEHPVDA : 288
Fa             : EGVYTWRVLSANGTEVRSANVSLVLYHQPEFGLSAPPVLFGEPFRAVCVVRDYYPRRSVRLRWFADEHPVDA : 288
SC             : EGVYTWRVLSANGTEVRSANVSLLLYSQPEFGLSAPPVLFGEPFRAVCVVRDYYPRRSVRLRWFADEHPVDA : 281
Bartha-K61     : EGVYTWRVLSANGTEVRSANVSLLLYSQPEFGLSAPPVLFGEPFRAVCVVRDYYPRRSVRLRWFADEHPVDA : 281
                 EGVYTWRVLSaNGTEVrSANVSL6LYhQPEFGLSAPPVLFGEPFRAVCVVRDYYPRRSVRLRWFADEHPVDA
```

```
                *         300         *         320         *         340         *         360
HeN21      : AFVTNSTVADELGRRTRVSVVNVTRADVPGLAAADDADALAPSLRCEAVWYRDSVASQRFSEALRPHVYHPA : 360
HuB20      : AFVTNSTVADELGRRTRVSVVNVTRADVPGLAAADDADALAPSLRCEAVWYRDSVASQRFSEALRPHVYHPA : 360
hSD-1      : AFVTNSTVADELGRRTRVSVVNVTRADVPGLAAADDADALAPSLRCEAVWYRDSVASQRFSEALRPHVYHPA : 360
HeN1       : AFVTNSTVADELGRRTRVSVVNVTRADVPGLAAADDADALAPSLRCEAVWYRDSVASQRFSEALRPHVYHPA : 360
HLJ8       : AFVTNSTVADELGRRTRVSVVNVTRADVPGLAAADDADALAPSLRCEAVWYRDSVASQRFSEALRPHVYHPA : 360
HN1201     : AFVTNSTVADELGRRTRVSVVNVTRADVPGLAAADDADALAPSLRCEAVWYRDSVASQRFSEALRPHVYHPA : 360
HNB        : AFVTNSTVADELGRRTRVSVVNVTRADVPGLAAADDADALAPSLRCEAVWYRDSVASQRFSEALRPHVYHPA : 360
HNX        : AFVTNSTVADELGRRTRVSVVNVTRADVPGLAAADDADALAPSLRCEAVWYRDSVASQRFSEALRPHVYHPA : 360
JS-2012    : AFVTNSTVADELGRHTRVSVVNVTRADVPGLAAADDADALAPSLRCEAVWYRDSVASQRFSEALRPHVYHPA : 360
TJ         : AFVTNSTVADELGRRTRVSVVNVTRADVPGLAAADDADALAPSLRCEAVWYRDSVASQRFSEALRPHVYHPA : 360
Ea         : AFVTNSTVADELGRRTRVSVVNVTRADVPGLAAADDADALAPSLRCEAVWYRDSVASQRFSEALRPHVYHPA : 360
Fa         : AFVTNSTVADELGRRTRVSVVNVTRADVPGLAAADDADALAPSLRCEAVWYRDSVASQRFSEALRPHVYHPA : 360
SC         : AFVTNSTVADELGRRTRVSVVNVTRADVPGLAAADDADALAPSLRCEAVWYRDSVASQRFSEALRPHVYHPA : 353
Bartha-K61 : AFVTNSTVADELGRRTRVSVVNVTRADVPGLAAADDADALAPSLRCEAVWYRDSVASQRFSEALRPHVYHPA : 353
             AFVTNSTVADELGRrTRVSVVNVTRADVPGLAAADDADALAPSLRCEAVWYRDSVASQRFSEALRPHVYHPA

                        *         380         *         400         *         420         *
HeN21      : AVSVRFVEGFAVCDGLCVPPEARLAWSDHAADTVYHLGACAEHPGLLNVRSARPLSDLDGPVDYTCRLEGMP : 432
HuB20      : AVSVRFVEGFAVCDGLCVPPEARLAWSDHAADTVYHLGACAEHPGLLNVRSARPLSDLDGPVDYTCRLEGMP : 432
hSD-1      : AVSVRFVEGFAVCDGLCVPPEARLAWSDHAADTVYHLGACAEHPGLLNVRSARPLSDLDGPVDYTCRLEGMP : 432
HeN1       : AVSVRFVEGFAVCDGLCVPPEARLAWSDHAADTVYHLGACAEHPGLLNVRSARPLSDLDGPVDYTCRLEGMP : 432
HLJ8       : AVSVRFVEGFAVCDGLCVPPEARLAWSDHAADTVYHLGACAEHPGLLNVRSARPLSDLDGPVDYTCRLEGMP : 432
HN1201     : AVSVRFVEGFAVCDGLCVPPEARLAWSDHAADTVYHLGACAEHPGLLNVRSARPLSDLDGPVDYTCRLEGMP : 432
HNB        : AVSVRFVEGFAVCDGLCVPPEARLAWSDHAADTVYHLGACAEHPGLLNVRSARPLSDLDGPVDYTCRLEGMP : 432
HNX        : AVSVRFVEGFAVCDGLCVPPEARLAWSDHAADTVYHLGACAEHPGLLNVRSARPLSDLDGPVDYTCRLEGMP : 432
JS-2012    : AVSVRFVEGFAVCDGLCVPPEARLAWSDHAADTVYHLGACAEHPGLLNVRSARPLSDLDGPVDYTCRLEGMP : 432
TJ         : AVSVRFVEGFAVCDGLCVPPEARLAWSDHAADTVYHLGACAEHPGLLNVRSARPLSDLDGPVDYTCRLEGMP : 432
Ea         : AVSVRFVEGFAVCDGLCVPPEARLAWSDHAADTVYHLGACAEHPGLLNVRSARPLSDLDGPVDYTCRLEGMP : 432
Fa         : AVSVRFVEGFAVCDGLCVPPEARLAWSDHAADTVYHLGACAEHPGLLNVRSARPLSDLDGPVDYTCRLEGMP : 432
SC         : AVSVRFVEGFAVCDGLCVPPEARLAWSDHAADTVYHLGACAEHPGLLNVRSARPLSDLDGPVDYTCRLEGMP : 425
Bartha-K61 : AVSVRFVEGFAVCDGLCVPPEARLAWSDHAADTVYHLGACAEHPGLLNVRSARPLSDLDGPVDYTCRLEGLP : 425
             AVSVRFVEGFAVCDGLCVPPEARLAWSDHAADTVYHLGACAEHPGLLNVRSARPLSDLDGPVDYTCRLEG6P
```

```
                        440         *        460         *        480
HeN21       : SQLPIFEDTQRYDASPTSVSWPVVTSMITVIAGIAILAIVLVIMATCVYYRRSAL* : 487
HuB20       : SQLPVFEDTQRYDASPTSVSWPVVTSMITVIAGIAILAIVLVIMATCVYYRRPVL* : 487
hSD-1       : SQLPIFEDTQRYDASPTSVSWPVVTSMITVIAGIAILAIVLVIMATCVYYRRSAL- : 487
HeN1        : SQLPIFEDTQRYDASPTSVSWPVVTSMITVIAGIAILAIVLVIMATCVYYRRSAL- : 487
HLJ8        : SQLPIFEDTQRYDASPTSVSWPVVTSMITVIAGIAILAIVLVIMATCVYYRRSAL- : 487
HN1201      : SQLPIFEDTQRYDASPTSVSWPVVTSMITVIAGIAILAIVLVIMATCVYYRRSAL- : 487
HNB         : SQLPIFEDTQRYDASPTSVSWPVVTSMITVIAGIAILAIVLVIMATCVYYRRSAL- : 487
HNX         : SQLPIFEDTQRYDASPTSVSWPVVTSMITVIAGIAILAIVLVIMATCVYYRRSAL- : 487
JS-2012     : SQLPIFEDTQRYDASPTSVSWPVVTSMITVIAGIAILAIVLVIMATCVYYRRSAL- : 487
TJ          : SQLPIFEDTQRYDASPTSVSWPVVTSMITVIAGIAILAIVLVIMATCVYYRRSAL- : 487
Ea          : SQLPIFEDTQRYDASPTSVSWPVVTSMITVIAGIAILAIVLVIMATCVYYRRSAL- : 487
Fa          : SQLPIFEDTQRYDASPTSVSWPVVTSMITVIAGIAILAIVLVIMATCVYYRRSAL- : 487
SC          : SQLPIFEDTQRYDASPTSVSWPVVTSMITVIAGIAILAIVLVIMATCVYYRRSAL- : 480
Bartha-K61  : SQLPVFEDTQRYDASPASVSWPVVSSMIVVIAGIGILAIVLVIMATCVYYRRAGP* : 480
              SQLP6FEDTQRYDASPtSVSWPVV3SMItVIAGIaILAIVLVIMATCVYYRRsal
```

gD

```
                         *        20         *        40         *        60         *
HeN21      : MLLAALLAALVARTTLGADVDAVPAPTFPPPAYPYTESWQLTLTTVPSPFVGPADVYHTRPLEDPCGVVALI :  72
HuB20      : MLLAALLVALVARTTLGADVDAVPAPTFPPPAYPYTESWQLTLTTVPSPFVGPADVYHTRPLEDPCGVVALI :  72
hSD-1      : MLLAALLAALVARTTLGADVDAVPAPTFPPPAYPYTESWQLTLTTVPSPFVGPADVYHTRPLEDPCGVVALI :  72
HeN1       : MLLAALLAALVARTTLGADVDAVPAPTFPPPAYPYTESWQLTLTTVPSPFVGPADVYHTRPLEDPCGVVALI :  72
HLJ8       : MLLAALLAALVARTTLGADVDAVPAPTFPPPAYPYTESWQLTLTTVPSPFVGPADVYHTRPLEDPCGVVALI :  72
HN1201     : MLLAALLAALVARTTLGADVDAVPAPTFPPPAYPYTESWQLTLTTVPSPFVGPADVYHTRPLEDPCGVVALI :  72
HNB        : MLLAALLAALVARTTLGADVDAVPAPTFPPPAYPYTESWQLTLTTVPSPFVGPADVYHTRPLEDPCGVVALI :  72
HNX        : MLLAALLAALVARTTLGADVDAVPAPTFPPPAYPYTESWQLTLTTVPSPFVGPADVYHTRPLEDPCGVVALI :  72
JS-2012    : MLLAALLAALVARTTLGADVDAVPAPTFPPPAYPYTESWQLTLTTVPSPFVGPADVYHTRPLEDPCGVVALI :  72
TJ         : MLLAALLAALVARTTLGADVDAVPAPTFPPPAYPYTESWQLTLTTVPSPFVGPADVYHTRPLEDPCGVVALI :  72
Ea         : MLLAALLAALVARTTLGADVDAVPAPTFPPPAYPYTESWQLTLTTVPSPFVGPADVYHTRPLEDPCGVVALI :  72
Fa         : MLLAALLAALVARTTLGADVDAVPAPTFPPPAYPYTESWQLTLTTVPSPFVGPADVYHTRPLEDPCGVVALI :  72
SC         : MLLAALLAALVARTTLGADVDAVPAPTFPPPAYPYTESWQLTLTTVPSPFVGPADVYHTRPLEDPCGVVALI :  72
Bartha-K61 : MLLAALLAALVARTTLGADVDAVPAPTFPPPAYPYTESWQLTLTTVPSPFVGPADVYHTRPLEDPCGVAALI :  72
             MLLAALLaALVARTTLGADVDAVPAPTFPPPAYPYTESWQLTLTTVPSPFVGPADVYHTRPLEDPCGVvALI

                    80         *       100         *       120         *       140
HeN21      : SDPQVDRLLNEAVAHRRPTYRAHVAWYRIADGCAHLLYFIEYADCDPRQIFGRCRRRTTPMWWTPSADYMFP : 144
HuB20      : SDPQVDRLLNEAVAHRRPTYRAHVAWYRIADGCAHLLYFIEYADCDPRQIFGRCRRRTTPMWWTPSADYMFP : 144
hSD-1      : SDPQVDRLLNEAVAHRRPTYRAHVAWYRIADGCAHLLYFIEYADCDPRQIFGRCRRRTTPMWWTPSADYMFP : 144
HeN1       : SDPQVDRLLNEAVAHRRPTYRAHVAWYRIADGCAHLLYFIEYADCDPRQIFGRCRRRTTPMWWTPSADYMFP : 144
HLJ8       : SDPQVDRLLNEAVAHRRPTYRAHVAWYRIADGCAHLLYFIEYADCDPRQIFGRCRRRTTPMWWTPSADYMFP : 144
HN1201     : SDPQVDRLLNEAVAHRRPTYRAHVAWYRIADGCAHLLYFIEYADCDPRQIFGRCRRRTTPMWWTPSADYMFP : 144
HNB        : SDPQVDRLLNEAVAHRRPTYRAHVAWYRIADGCAHLLYFIEYADCDPRQIFGRCRRRTTPMWWTPSADYMFP : 144
HNX        : SDPQVDRLLNEAVAHRRPTYRAHVAWYRIADGCAHLLYFIEYADCDPRQIFGRCRRRTTPMWWTPSADYMFP : 144
JS-2012    : SDPQVDRLLNEAVAHRRPTYRAHVAWYRIADGCAHLLYFIEYADCDPRQIFGRCRRRTTPMWWTPSADYMFP : 144
TJ         : SDPQVDRLLNEAVAHRRPTYRAHVAWYRIADGCAHLLYFIEYADCDPRQIFGRCRRRTTPMWWTPSADYMFP : 144
Ea         : SDPQVDRLLNEAVAHRRPTYRAHVAWYRIADGCAHLLYFIEYADCDPRQIFGRCRRRTTPMWWTPSADYMFP : 144
Fa         : SDPQVDRLLNEAVAHRRPTYRAHVAWYRIADGCAHLLYFIEYADCDPRQIFGRCRRRTTPMWWTPSADYMFP : 144
SC         : SDPQVDRLLNEAVAHRRPTYRAHVAWYRIADGCAHLLYFIEYADCDPRQIFGRCRRRTTPMWWTPSADYMFP : 144
Bartha-K61 : SDPQVDRLLSEAVAHRRPTYRAHVAWYRIADGCAHLLYFIEYADCDPRQIFGRCRRRTTPMWWTPSADYMFP : 144
             SDPQVDRLLnEAVAHRRPTYRAHVAWYRIADGCAHLLYFIEYADCDPRQIFGRCRRRTTPMWWTPSADYMFP
```

```
                       *        160         *        180         *        200         *
HeN21       : TEDELGLLMVAPGRFNEGQYRRLVSVDGVNILTDFMVALPEGQECPFARVDQHRTYKFGACWSDDSFKRGVD : 216
HuB20       : TEDELGLLMVAPGRFNEGQYRRLVSVDGVNILTDFMVALPEGQECPFARVDQHRTYKFGACWSDDSFKRGVD : 216
hSD-1       : TEDELGLLMVAPGRFNEGQYRRLVSVDGVNILTDFMVALPEGQECPFARVDQHRTYKFGACWSDDSFKRGVD : 216
HeN1        : TEDELGLLMVAPGRFNEGQYRRLVSVDGVNILTDFMVALPEGQECPFARVDQHRTYKFGACWSDDSFKRGVD : 216
HLJ8        : TEDELGLLMVAPGRFNEGQYRRLVSVDGVNILTDFMVALPEGQECPFARVDQHRTYKFGACWSDDSFKRGVD : 216
HN1201      : TEDELGLLMVAPGRFNEGQYRRLVSVDGVNILTDFMVALPEGQECPFARVDQHRTYKFGACWSDDSFKRGVD : 216
HNB         : TEDELGLLMVAPGRFNEGQYRRLVSVDGVNILTDFMVALPEGQECPFARVDQHRTYKFGACWSDDSFKRGVD : 216
HNX         : TEDELGLLMVAPGRFNEGQYRRLVSVDGVNILTDFMVALPEGQECPFARVDQHRTYKFGACWSDDSFKRGVD : 216
JS-2012     : TEDELGLLMVAPGRFNEGQYRRLVSVDGVNILTDFMVALPEGQECPFARVDQHRTYKFGACWSDDSFKRGVD : 216
TJ          : TEDELGLLMVAPGRFNEGQYRRLVSVDGVNILTDFMVALPEGQECPFARVDQHRTYKFGACWSDDSFKRGVD : 216
Ea          : TEDELGLLMVAPGRFNEGQYRRLVSVDGVNILTDFMVALPEGQECPFARVDQHRTYKFGACWSDDSFKRGVD : 216
Fa          : TEDELGLLMVAPGRFNEGQYRRLVSVDGVNILTDFMVALPEGQECPFARVDQHRTYKFGACWSDDSFKRGVD : 216
SC          : TEDELGLLMVAPGRFNEGQYRRLVSVDGVNILTDFMVALPEGQECPFARVDQHRTYKFGACWSDDSFKRGVD : 216
Bartha-K61  : TEDELGLLMVAPGRFNEGQYRRLVSVDGVNILTDFMVALPEGQECPFARVDQHRTYKFGACWNDESFRRGVD : 216
              TEDELGLLMVAPGRFNEGQYRRLVSVDGVNILTDFMVALPEGQECPFARVDQHRTYKFGACWsDdSF4RGVD

               220         *        240         *        260         *        280
HeN21       : VMRFLTPFYQQPPHREVVNYWYRKNGRTLPRAYAAATPYAIDPARPSAGSPRPRPRPRPRPR--PKPEPAPA : 286
HuB20       : VMRFLTPFYQQPPHREVVNYWYRKNGRTLPRAYAAATPYAIDPARPSAGSPRPRPRPRPRPK----PEPAPA : 284
hSD-1       : VMRFLTPFYQQPPHREVVNYWYRKNGRTLPRAYAAATPYAIDPARPSAGSPRPRPRPRPRPR--PKPEPAPA : 286
HeN1        : VMRFLTPFYQQPPHREVVNYWYRKNGRTLPRAYAAATPYAIDPARPSAGSPRPRPRPRPRPR--PKPEPAPA : 286
HLJ8        : VMRFLTPFYQQPPHREVVNYWYRKNGRTLPRAYAAATPYAIDPARPSAGSPRPRPRPRPRPR--PKPEPAPA : 286
HN1201      : VMRFLTPFYQQPPHREVVNYWYRKNGRTLPRAYAAATPYAIDPARPSAGSPRPRPRPRPRPR--PKPEPAPA : 286
HNB         : VMRFLTPFYQQPPHREVVNYWYRKNGRTLPRAYAAATPYAIDPARPSAGSPRPRPRPRPRPR--PKPEPAPA : 286
HNX         : VMRFLTPFYQQPPHREVVNYWYRKNGRTLPRAYAAATPYAIDPARPSAGSPRPRPRPRPRPR--PKPEPAPA : 286
JS-2012     : VMRFLTPFYQQPPHREVVNYWYRKNGRTLPRAYAAATPYAIDPARPSAGSPRPRPRPRPRPR--PKPEPAPA : 286
TJ          : VMRFLTPFYQQPPHREVVNYWYRKNGRTLPRAYAAATPYAIDPARPSAGSPRPRPRPRPRPR--PKPEPAPA : 286
Ea          : VMRFLTPFYQQPPHREVVNYWYRKNGRTLPRAYAAATPYAIDPARPSAGSPRPRPRPRPRPRPRPKPEPAPA : 288
Fa          : VMRFLTPFYQQPPHREVVNYWYRKNGRTLPRAYAAATPYAIDPARPSAGSPRPRPRPRPRPRPRPKPEPAPA : 288
SC          : VMRFLTPFYQQPPHREVVNYWYRKNGRTLPRAYAAATPYAIDPARPSAGSPRPRPRPRPRPRPRPKPEPAPA : 288
Bartha-K61  : VMRFLTPFYQQPPHREVVNYWYRKNGRTLPRAYAAATPYAIDPARPSAGSPRPRPRPRPRPK----PEPAPV : 284
              VMRFLTPFYQQPPHREVVNYWYRKNGRTLPRAYAAATPYAIDPARPSAGSPRPRPRPRPRP4  pkPEPAPa
```

2

```
                      *        300         *        320         *        340         *        360
HeN21        : TPAPPGRLPEPATRDHAAGGRPTPRPPRPETPHRPFAPPAVVPSGWPQPAEPFPPRTTAAPGVSRHRSVIVG : 358
HuB20        : TPAPPGRLPEPATRDHAAGGRPTPRPPRPETPHRPFAPPAVVPSGWPQPAEPFPPRTTAAPGVSRHRSVIVG : 356
hSD-1        : TPAPPGRLPEPATRDHAAGGRPTPRPPRPETPHRPFAPPAVVPSGWPQPAEPFPPRTTAAPGVSRHRSVIVG : 358
HeN1         : TPAPPGRLPEPATRDHAAGGRPTPRPPRPETPHRPFAPPAVVPSGWPQPAEPFPPRTTAAPGVSRHRSVIVG : 358
HLJ8         : TPAPPGRLPEPATRDHAAGGRPTPRPPRPETPHRPFAPPAVVPSGWPQPAEPFPPRTTAAPGVSRHRSVIVG : 358
HN1201       : TPAPPGRLPEPATRDHAAGGRPTPRPPRPETPHRPFAPPAVVPSGWPQPAEPFPPRTTAAPGVSRHRSVIVG : 358
HNB          : TPAPPGRLPEPATRDHAAGGRPTPRPPRPETPHRPFAPPAVVPSGWPQPAEPFPPRTTAAPGVSRHRSVIVG : 358
HNX          : TPAPPGRLPEPATRDHAAGGRPTPRPPRPETPHRPFAPPAVVPSGWPQPAEPFPPRTTAAPGVSRHRSVIVG : 358
JS-2012      : TPAPPGRLPEPATRDHAAGGRPTPRPPRPETPHRPFAPPAVVPSGWPQPAEPFPPRTTAAPGVSRHRSVIVG : 358
TJ           : TPAPPGRLPEPATRDHAAGGRPTPRPPRPETPHRPFAPPAVVPSGWPQPAEPFPPRTTAAPGVSRHRSVIVG : 358
Ea           : TPAPPGRLPEPATRDHAAGGRPTPRPPRPETPHRPFAPPAVVPSGWPQPVEPFPPRTTAAPGVSRHRSVIVG : 360
Fa           : TPAPPGRLPEPATRDHAAGGRPTPRPPRPETPHRPFAPPAVVPSGWPQPVEPFPPRTTAAPGVSRHRSVIVG : 360
SC           : TPAPPGRLPEPATRDHAAGGRPTPRPPRPETPHRPFAPPAVVPSGWPQPVEPFPPRTTAAPGVSRHRSVIVG : 360
Bartha-K61   : TPAPPGRLPEPATRDHAAGGHPTPRPPRPETPHRPFAPPAVVPSGWPQPAEPFQPRTPAAPGVSRHRSVIVG : 356
               TPAPPGRLPEPATRDHAAGGrPTPRPPRPETPHRPFAPPAVVPSGWPQPaEPFpPRTtAAPGVSRHRSVIVG

                        *        380         *        400
HeN21        : TGTAMGALLVGVCVYIFFRLRGAKGYRLLGGPADADELKAQPGP* : 402
HuB20        : TGTAMGALLVGVCVYIFFRLRGAKGYRLLGGPADADELKAQPGP* : 400
hSD-1        : TGTAMGALLVGVCVYIFFRLRGAKGYRLLGGPADADELKAQPGP- : 402
HeN1         : TGTAMGALLVGVCVYIFFRLRGAKGYRLLGGPADADELKAQPGP- : 402
HLJ8         : TGTAMGALLVGVCVYIFFRLRGAKGYRLLGGPADADELKAQPGP- : 402
HN1201       : TGTAMGALLVGVCVYIFFRLRGAKGYRLLGGPADADELKAQPGP- : 402
HNB          : TGTAMGALLVGVCVYIFFRLRGAKGYRLLGGPADADELKAQPGP- : 402
HNX          : TGTAMGALLVGVCVYIFFRLRGAKGYRLLGGPADADELKAQPGP- : 402
JS-2012      : TGTAMGALLVGVCVYIFFRLRGAKGYRLLGGPADADELKAQPGP- : 402
TJ           : TGTAMGALLVGVCVYIFFRLRGAKGYRLLGGPADADELKAQPGP- : 402
Ea           : TGTAMGALLVGVCVYIFFRLRGAKGYRLLGGPADADELKAQPGP- : 404
Fa           : TGTAMGALLVGVCVYIFFRLRGAKGYRLLGGPADADELKAQPGP- : 404
SC           : TGTAMGALLVGVCVYIFFRLRGAKGYRLLGGPADADELKAQPGP- : 404
Bartha-K61   : TGTAMGALLVGVCVYIFFRLRGAKGYRLLGGPADTDELKAQPGP* : 400
               TGTAMGALLVGVCVYIFFRLRGAKGYRLLGGPADaDELKAQPGP
```

gE

```
                    *        20         *        40         *        60         *
HeN21    : MRPFLLRAAQLLALLALALSTEAPSLSAETTPGPVTEVPSPSAEVWDDLSTEADDDDLNGDLDGDDRRAGFGSAL :  75
HuB20    : MRPFLLRAAQLLALLALALSTEAPSLSAETTPGPVTEVPSPSAEVWDDRSTEAGDDDLN----GDDRRAGFGSAL :  71
hSD-1    : MRPFLLRAAQLLALLALALSTEAPSLSAETTPGPVTEVPSPSAEVWDDLSTEADDDDLNGDLDGDDRRAGFGSAL :  75
HeN1     : MRPFLLRAAQLLALLALALSTEAPSLSAETTPGPVTEVPSPSAEVWDDLSTEADDDDLNGDLDGDDRRAGFGSAL :  75
HLJ8     : MRPFLLRAAQLLALLALALSTEAPSLSAETTPGPVTEVPSPSAEVWDDLSTEADDDDLNGDLDGDDRRAGFGSAL :  75
HN1201   : MRPFLLRAAQLLALLALALSTEAPSLSAETTPGPVTEVPSPSAEVWDDLSTEADDDDLNGDLDGDDRRAGFGSAL :  75
HNB      : MRPFLLRAAQLLALLALALSTEAPSLSAETTPGPVTEVPSPSAEVWDDLSTEADDDDLNGDLDGDDRRAGFGSAL :  75
HNX      : MRPFLLRAAQLLALLALALSTEAPSLSAETTPGPVTEVPSPSAEVWDDLSTEADDDDLNGDLDGDDRRAGFGSAL :  75
JS-2012  : MRPFLLRAAQLLALLALALSTEAPSLSAETTPGPVTEVPSPSAEVWDDLSTEADDDDLNGDLDGDDRRAGFGSAL :  75
TJ       : MRPFLLRAAQLLALLALALSTEAPSLSAETTPGPVTEVPSPSAEVWDDLSTEADDDDLNGDLDGDDRRAGFGSAL :  75
Ea       : MRPFLLRAAQLLALLALALSTEAPSLSAETTPGPVTEVPSPSAEVWDDLSTEAGDDDLNGDLDGDDRRAGFGSAL :  75
Fa       : MRPFLLRAAQLLALLALALSTEAPSLSAETTPGPVTEVPSPSAEVWDDLSTEAGDDDLNGDLDGDDRRAGFGSAL :  75
SC       : MRPFLLRAAQLLALLALALSTEAPSLSAETTPGPVTEVPSPSAEVWDDLSTEAGDDDLNGDLDGDDRRAGFGSAL :  75
           MRPFLLRAAQLLALLALALSTEAPSLSAETTPGPVTEVPSPSAEVWDDlSTEA DDDLNgdldGDDRRAGFGSAL

              80         *       100         *       120         *       140         *
HeN21    : ASLREAPPAHLVNVSEGANFTLDARGDGAVLAGIWTFLPVRGCDAVSVTTVCFETACHPDLVLGRACVPEAPEMG : 150
HuB20    : ASLREAPPAHLVNVSEGANFTLDARGDGAVLAGIWTFLPVRGCDAVSVTTVCFETACHPDLVLGRACVPEAPEMG : 146
hSD-1    : ASLREAPPAHLVNVSEGANFTLDARGDGAVLAGIWTFLPVRGCDAVSVTTVCFETACHPDLVLGRACVPEAPEMG : 150
HeN1     : ASLREAPPAHLVNVSEGANFTLDARGDGAVLAGIWTFLPVRGCDAVSVTTVCFETACHPDLVLGRACVPEAPEMG : 150
HLJ8     : ASLREAPPAHLVNVSEGANFTLDARGDGAVLAGIWTFLPVRGCDAVSVTTVCFETACHPDLVLGRACVPEAPEMG : 150
HN1201   : ASLREAPPAHLVNVSEGANFTLDARGDGAVLAGIWTFLPVRGCDAVSVTTVCFETACHPDLVLGRACVPEAPEMG : 150
HNB      : ASLREAPPAHLVNVSEGANFTLDARGDGAVLAGIWTFLPVRGCDAVSVTTVCFETACHPDLVLGRACVPEAPEMG : 150
HNX      : ASLREAPPAHLVNVSEGANFTLDARGDGAVLAGIWTFLPVRGCDAVSVTTVCFETACHPDLVLGRACVPEAPEMG : 150
JS-2012  : ASLREAPPAHLVNVSEGANFTLDARGDGAVLAGIWTFLPVRGCDAVSVTTVCFETACHPDLVLGRACVPEAPEMG : 150
TJ       : ASLREAPPAHLVNVSEGANFTLDARGDGAVLAGIWTFLPVRGCDAVAVTTVCFETACHPDLVLGRACVPEAPEMG : 150
Ea       : ASLREAPPAHLVNVSEGANFTLDARGDGAVLAGIWTFLPVRGCDAVSVTTVCFETACHPDLVLGRACVPEAPEMG : 150
Fa       : ASLREAPPAHLVNVSEGANFTLDARGDGAVLAGIWTFLPVRGCDAVSVTTVCFETACHPDLVLGRACVPEAPEMG : 150
SC       : ASLREAPPAHLVNVSEGANFTLDARGDGAVLAGIWTFLPVRGCDAVSVTTVCFETACHPDLVLGRACVPEAPEMG : 150
           ASLREAPPAHLVNVSEGANFTLDARGDGAVLAGIWTFLPVRGCDAVsVTTVCFETACHPDLVLGRACVPEAPEMG
```

```
                  160         *       180         *       200         *       220
HeN21    : IGDYLPPEVPRLRREPPIVTPERWSPHLSVLRATPNDTGLYTLHDASGPRAVFFVAVGDRPPAPADPVGPARHEP : 225
HuB20    : IGDYLPPEVPRLRREPPIVTPERWSPHLSVLRATPNDTGLYTLHDASGPRAVFFVAVGDRPPAPADPVGPARHEP : 221
hSD-1    : IGDYLPPEVPRLRREPPIVTPERWSPHLSVLRATPNDTGLYTLHDASGPRAVFFVAVGDRPPAPADPVGPARHEP : 225
HeN1     : IGDYLPPEVPRLRREPPIVTPERWSPHLSVLRATPNDTGLYTLHDASGPRAVFFVAVGDRPPAPADPVGPARHEP : 225
HLJ8     : IGDYLPPEVPRLRREPPIVTPERWSPHLSVLRATPNDTGLYTLHDASGPRAVFFVAVGDRPPAPADPVGPARHEP : 225
HN1201   : IGDYLPPEVPRLRREPPIVTPERWSPHLSVLRATPNDTGLYTLHDASGPRAVFFVAVGDRPPAPADPVGPARHEP : 225
HNB      : IGDYLPPEVPRLRREPPIVTPERWSPHLSVLRATPNDTGLYTLHDASGPRAVFFVAVGDRPPAPADPVGPARHEP : 225
HNX      : IGDYLPPEVPRLRREPPIVTPERWSPHLSVLRATPNDTGLYTLHDASGPRAVFFVAVGDRPPAPADPVGPARHEP : 225
JS-2012  : IGDYLPPEVPRLRREPPIVTPERWSPHLSVLRATPNDTGLYTLHDASGPRAVFFVAVGDRPPAPADPVGPARHEP : 225
TJ       : IGDYLPPEVPRLRREPPIVTPERWSPHLSVLRATPNDTGLYTLHDASGPRAVFFVAVGDRPPAPADPVGPARHEP : 225
Ea       : IGDYLPPEVPRLRREPPIVTPERWSPHLSVLRATPNDTGLYTLHDASGPRAVFFVAVGDRPPAPADPVGPARHEP : 225
Fa       : IGDYLPPEVPRLRREPPIVTPERWSPHLSVLRATPNDTGLYTLHDASGPRAVFFVAVGDRPPAPADPVGPARHEP : 225
SC       : IGDYLPPEVPRLRREPPIVTPERWSPHLSVLRATPNDTGLYTLHDASGPRAVFFVAVGDRPPAPADPVGPARHEP : 225
           IGDYLPPEVPRLRREPPIVTPERWSPHLSVLRATPNDTGLYTLHDASGPRAVFFVAVGDRPPAPADPVGPARHEP

               *       240         *       260         *       280         *       300
HeN21    : RFHALGFHSQLFSPGDTFDLMPRVVSDMGDSRENFTATLDWYYARAPPRCLLYYVYEPCIYHPRAPECLRPVDPA : 300
HuB20    : RFHALGFHSQLFSPGDTFDLMPRVVSDMGDSRENFTATLDWYYARAPPRCLLYYVYEPCIYHPRAPECLRPVDPA : 296
hSD-1    : RFHALGFHSQLFSPGDTFDLMPRVVSDMGDSRENFTATLDWYYARAPPRCLLYYVYEPCIYHPRAPECLRPVDPA : 300
HeN1     : RFHALGFHSQLFSPGDTFDLMPRVVSDMGDSRENFTATLDWYYARAPPRCLLYYVYEPCIYHPRAPECLRPVDPA : 300
HLJ8     : RFHALGFHSQLFSPGDTFDLMPRVVSDMGDSRENFTATLDWYYARAPPRCLLYYVYEPCIYHPRAPECLRPVDPA : 300
HN1201   : RFHALGFHSQLFSPGDTFDLMPRVVSDMGDSRENFTATLDWYYARAPPRCLLYYVYEPCIYHPRAPECLRPVDPA : 300
HNB      : RFHALGFHSQLFSPGDTFDLMPRVVSDMGDSRENFTATLDWYYARAPPRCLLYYVYEPCIYHPRAPECLRPVDPA : 300
HNX      : RFHALGFHSQLFSPGDTFDLMPRVVSDMGDSRENFTATLDWYYARAPPRCLLYYVYEPCIYHPRAPECLRPVDPA : 300
JS-2012  : RFHALGFHSQLFSPGDTFDLMPRVVSDMGDSRENFTATLDWYYARAPPRCLLYYVYEPCIYHPRAPECLRPVDPA : 300
TJ       : RFHALGFHSQLFSPGDTFDLMPRVVSDMGDSRENFTATLDWYYARAPPRCLLYYVYEPCIYHPRAPECLRPVDPA : 300
Ea       : RFHALGFHSQLFSPGDTFDLMPRVVSDMGDSRENFTATLDWYYARAPPRCLLYYVYEPCIYHPRAPECLRPVDPA : 300
Fa       : RFHALGFHSQLFSPGDTFDLMPRVVSDMGDSRENFTATLDWYYARAPPRCLLYYVYEPCIYHPRAPECLRPVDPA : 300
SC       : RFHALGFHSQLFSPGDTFDLMPRVVSDMGDSRENFTATLDWYYARAPPRCLLYYVYEPCIYHPRAPECLRPVDPA : 300
           RFHALGFHSQLFSPGDTFDLMPRVVSDMGDSRENFTATLDWYYARAPPRCLLYYVYEPCIYHPRAPECLRPVDPA
```

```
                      *        320         *        340         *        360         *
HeN21    : CSFTSPARARLVARRAYASCSPLLGDRWLTACPFDAFGEEVHTNATADESGLYVLVMTHNGHVATWDYTLVATAA : 375
HuB20    : CSFTSPARARLVARRAYASCSPLLGDRWLTACPFDAFGEEVHTNATADESGLYVLVMTHNGHVATWDYTLVATAA : 371
hSD-1    : CSFTSPARARLVARRAYASCSPLLGDRWLTACPFDAFGEEVHTNATADESGLYVLVMTHNGHVATWDYTLVATAA : 375
HeN1     : CSFTSPARARLVARRAYASCSPLLGDRWLTACPFDAFGEEVHTNATADESGLYVLVMTHNGHVATWDYTLVATAA : 375
HLJ8     : CSFTSPARARLVARRAYASCSPLLGDRWLTACPFDAFGEEVHTNATADESGLYVLVMTHNGHVATWDYTLVATAA : 375
HN1201   : CSFTSPARARLVARRAYASCSPLLGDRWLTACPFDAFGEEVHTNATADESGLYVLVMTHNGHVATWDYTLVATAA : 375
HNB      : CSFTSPARARLVARRAYASCSPLLGDRWLTACPFDAFGEEVHTNATADESGLYVLVMTHNGHVATWDYTLVATAA : 375
HNX      : CSFTSPARARLVARRAYASCSPLLGDRWLTACPFDAFGEEVHTNATADESGLYVLVMTHNGHVATWDYTLVATAA : 375
JS-2012  : CSFTSPARARLVARRAYASCSPLLGDRWLTACPFDAFGEEVHTNATADESGLYVLVMTHNGHVATWDYTLVATAA : 375
TJ       : CSFTSPARARLVARRAYASCSPLLGDRWLTACPFDAFGEEVHTNATADESGLYVLVMTHNGHVATWDYTLVATAA : 375
Ea       : CSFTSPARARLVARRAYASCSPLLGDRWLTACPFDAFGEEVHTNATADESGLYVLVMTHNGHVATWDYTLVATAA : 375
Fa       : CSFTSPARARLVARRAYASCSPLLGDRWLTACPFDAFGEEVHTNATADESGLYVLVMTHNGHVATWDYTLVATAA : 375
SC       : CSFTSPARARLVARRAYASCSPLLGDRWLTACPFDAFGEEVHTNATADESGLYVLVMTHNGHVATWDYTLVATAA : 375
           CSFTSPARARLVARRAYASCSPLLGDRWLTACPFDAFGEEVHTNATADESGLYVLVMTHNGHVATWDYTLVATAA

             380         *        400         *        420         *        440         *
HeN21    : EYVTVIKELTAPARAPGTPWGPGGGDDAIYVDGVTTPAPPARPWNPYGRTTPGRLFVLALGSFVMTCVVGGAIWL : 450
HuB20    : EYVTVIKELTAPARAPGTPWGPGGGDDAIYVDGVTTPAPPARPWNPYGRTTPGRLFVLALGSFVMTCVVGGAVWL : 446
hSD-1    : EYVTVIKELTAPARAPGTPWGPGGGDDAIYVDGVTTPAPPARPWNPYGRTTPGRLFVLALGSFVMTCVVGGAIWL : 450
HeN1     : EYVTVIKELTAPARAPGTPWGPGGGDDAIYVDGVTTPAPPARPWNPYGRTTPGRLFVLALGSFVMTCVVGGAIWL : 450
HLJ8     : EYVTVIKELTAPARAPGTPWGPGGGDDAIYVDGVTTPAPPARPWNPYGRTTPGRLFVLALGSFVMTCVVGGAIWL : 450
HN1201   : EYVTVIKELTAPARAPGTPWGPGGGDDAIYVDGVTTPAPPARPWNPYGRTTPGRLFVLALGSFVMTCVVGGAIWL : 450
HNB      : EYVTVIKELTAPARAPGTPWGPGGGDDAIYVDGVTTPAPPARPWNPYGRTTPGRLFVLALGSFVMTCVVGGAIWL : 450
HNX      : EYVTVIKELTAPARAPGTPWGPGGGDDAIYVDGVTTPAPPARPWNPYGRTTPGRLFVLALGSFVMTCVVGGAIWL : 450
JS-2012  : EYVTVIKELTAPARAPGTPWGPGGGDDAIYVDGVTTPAPPARPWNPYGRTTPGRLFVLALGSFVMTCVVGGAIWL : 450
TJ       : EYVTVIKELTAPARAPGTPWGPGGGDDAIYVDGVTTPAPPARPWNPYGRTTPGRLFVLALGSFVMTCVVGGAVWL : 450
Ea       : EYVTVIKELTAPARAPGTPWGPGGGDDPIYVDGVTTPAPPARPWNPYGRTTPGRLFVLALGSFVMTCVVGGAVWL : 450
Fa       : EYVTVIKELTAPARAPGTPWGPGGGDDPIYVDGVTTPAPPARPWNPYGRTTPGRLFVLALGSFVMTCVVGGAVWL : 450
SC       : EYVTVIKELTAPARAPGTPWGPGGGDDPIYVDGVTTPAPPARPWNPYGRTTPGRLFVLALGSFVMTCVVGGAVWL : 450
           EYVTVIKELTAPARAPGTPWGPGGGDDaIYVDGVTTPAPPARPWNPYGRTTPGRLFVLALGSFVMTCVVGGA6WL
```

```
                      460         *       480         *       500         *       520
HeN21    : CVLCSRRRAASRPFRVPTRARTHMLSPVYTSLPTHEDYYDGDDDDDEEAGVIRRRPASPSGDSGYEGPYASLDPE : 525
HuB20    : CVLCSRRRAASRPFRVPTRARTHMLSPVYTSLPTHEDYYDGDDDD-EEAGVVRRRPASPGGDSGYEGPYASLDPE : 520
hSD-1    : CVLCSRRRAASRPFRVPTRARTHMLSPVYTSLPTHEDYYDGDDDDDEEAGVIRRRPASPSGDSGYEGPYASLDPE : 525
HeN1     : CVLCSRRRAASRPFRVPTRARTHMLSPVYTSLPTHEDYYDGDDDDDEEAGVIRRRPASPSGDSGYEGPYASLDPE : 525
HLJ8     : CVLCSRRRAASRPFRVPTRARTHMLSPVYTSLPTHEDYYDGDDDDDEEAGVIRRRPASPGGDSGYEGPYASLDPE : 525
HN1201   : CVLCSRRRAASRPFRVPTRARTHMLSPVYTSLPTHEDYYDGDDDDDEEAGVIRRRPASPSGDSGYEGPYASLDPE : 525
HNB      : CVLCSRRRAASRPFRVPTRARTHMLSPVYTSLPTHEDYYDGDDDDDEEAGVIRRRPASPSGDSGYEGPYASLDPE : 525
HNX      : CVLCSRRRAASRPFRVPTRARTHMLSPVYTSLPTHEDYYDGDDDDDEEAGVIRRRPASPSGDSGYEGPYASLDPE : 525
JS-2012  : CVLCSRRRAASRPFRVPTRARTHMLSPVYTSLPTHEDYYDGDDDDDEEAGVIRRRPASPSGDSGYEGPYASLDPE : 525
TJ       : CVLCSRRRAASRPFRVPTRARTHMLSPVYTSLPTHEDYYDGDDDDDEEAGVIRRRPASPGGDSGYEGPYASLDPE : 525
Ea       : CVLCSRRRAASRPFRVPTRARTHMLSPVYTSLPTHEDYYDGDDDD-EEAGVIRRRPASPGGDSGYEGSYASLDPE : 524
Fa       : CVLCSRRRAASRPFRVPTRARTHMLSPVYTSLPTHEDYYDGDDDD-EEAGVIRRRPASPGGDSGYEGSYASLDPE : 524
SC       : CVLCSRRRAASRPFRVPTRARTHMLSPVYTSLPTHEDYYDGDDDD-EEAGVIRRRPASPGGDSGYEGSYASLDPE : 524
           CVLCSRRRAASRPFRVPTRARTHMLSPVYTSLPTHEDYYDGDDDD EEAGV6RRRPASP GDSGYEGpYASLDPE

                 *       540         *       560         *       580
HeN21    : DEFSSDEDDGLYVRPEEAPRSGFDVWFRDPEKPEVTNGPNYGVTANRLLMSRPA* : 579
HuB20    : DEFSSDEDDGLYVRPEEAPRSGFDVWFRDPEKPEVTNGPNYGVTANRLLMSRPA* : 574
hSD-1    : DEFSSDEDDGLYVRPEEAPRSGFDVWFRDPEKPEVTNGPNYGVTANRLLMSRPA- : 579
HeN1     : DEFSSDEDDGLYVRPEEAPRSGFDVWFRDPEKPEVTNGPNYGVTANRLLNARPA- : 579
HLJ8     : DEFSSDEDDGLYVRPEEAPRSGFDVWFRDPEKPEVTNGPNYGVTANRLLMSRPA- : 579
HN1201   : DEFSSDEDDGLYVRPEEAPRSGFDVWFRDPEKPEVTNGPNYGVTANRLLMSRPA- : 579
HNB      : DEFSSDEDDGLYVRPEEAPRSGFDVWFRDPEKPEVTNGPNYGVTANRLLMSRPA- : 579
HNX      : DEFSSDEDDGLYVRPEEAPRSGFDVWFRDPEKPEVTNGPNYGVTANRLLMSRPA- : 579
JS-2012  : DEFSSDEDDGLYVRPEEAPRSGFDVWFRDPEKPEVTNGPNYGVTANRLLMSRPA- : 579
TJ       : DEFSSDEDDGLYVRPEEAPRSGFDVWFRDPEKPEVTNGPNYGVTANRLLMSRPA- : 579
Ea       : DEFSSDEDDGLYVRPEEAPRSGFDVWFRDPEKPEVTNGPNYGVTANRLLMSRPA- : 578
Fa       : DEFSSDEDDGLYVRPEEAPRSGFDVWFRDPEKPEVTNGPNYGVTANRLLMSRPA- : 578
SC       : DEFSSDEDDGLYVRPEEAPRSGFDVWFRDPEKPEVTNGPNYGVTANRLLMSRPA- : 578
           DEFSSDEDDGLYVRPEEAPRSGFDVWFRDPEKPEVTNGPNYGVTANRLLmsRPA
```

gG

```
                              *        20         *        40         *        60         *
HeN21       : MKWATWILALGLLVVRTVVAREAPRELCYGHPVHDDRRPVGPATDAQPVNPLAPANATGTDYSRGCEMRLLD :  72
HuB20       : MKWATWILALGLLVVRTVVAREAPRELCYGHPVHDDRRPVGPATDAQPVNPLAPANATGTDYSRGCEMRLLD :  72
hSD-1       : MKWATWILALGLLVVRTVVAREAPRELCYGHPVHDDRRPVGPATDAQPVNPLAPANATGTDYSRGCEMRLLD :  72
HeN1        : MKWATWILALGLLVVRTVVAREAPRELCYGHPVHDDRRPVGPATDAQPVNPLAPANATGTDYSRGCEMRLLD :  72
HLJ8        : MKWATWILALGLLVVRTVVAREAPRELCYGHPVHDDRRPVGPATDAQPVNPLAPANATGTDYSRGCEMRLLD :  72
HN1201      : MKWATWILALGLLVVRTVVAREAPRELCYGHPVHDDRRPVGPATDAQPVNPLAPANATGTDYSRGCEMRLLD :  72
HNB         : MKWATWILALGLLVVRTVVAREAPRELCYGHPVHDDRRPVGPATDAQPVNPLAPANATGTDYSRGCEMRLLD :  72
HNX         : MKWATWILALGLLVVRTVVAREAPRELCYGHPVHDDRRPVGPATDAQPVNPLAPANATGTDYSRGCEMRLLD :  72
JS-2012     : MKWATWILALGLLVVRTVVAREAPRELCYGHPVHDDRRPVGPATDAQPVNPLAPANATGTDYSRGCEMRLLD :  72
TJ          : MKWATWILALGLLVVRTVVAREAPRELCYGHPVHDDRRPVGPATDAQPVNPLAPANATGTDYSRGCEMRLLD :  72
Ea          : MKWATWILALGLLVVRTVVAREAPRELCYGHPVHDDRRPVGPATDAQPVNPLAPANATGTDYSRGCEMRLLD :  72
Fa          : MKWATWILALGLLVVRTVVAREAPRELCYGHPVHDDRRPVGPATDAQPVNPLAPANATGTDYSRGCEMRLLD :  72
SC          : MKWATWILALGLLVVRTVVAREAPRELCYGHPVHDDRRPVGPATDAQPVNPLAPANATGTDYSRGCEMRLLD :  72
Bartha-K61  : MKWATWILALGLLVVRTVVAREAPRELCYGHPVHDDRRPVGPATDAQPVNPLAPANATGTDYSRGCEMRLLD :  72
              MKWATWILALGLLVVRTVVAREAPRELCYGHPVHDDRRPVGPATDAQPVNPLAPANATGTDYSRGCEMRLLD

                     80         *       100         *       120         *       140
HeN21       : PPLDVSSRSPDPVNVTVAWFFDGGHCKVPLVHREYYGCPGDAMPSVETCTGGYSYTRTRIDTLMEYALVNAS : 144
HuB20       : PPLDVSSRSSDPVNVTVAWFFDGGHCKVPLVHREYYGCPGDAMPSVETCTGGYSYTRTRIDTLMEYALVNAS : 144
hSD-1       : PPLDVSSRSPDPVNVTVAWFFDGGHCKVPLVHREYYGCPGDAMPSVETCTGGYSYTRTRIDTLMEYALVNAS : 144
HeN1        : PPLDVSSRSPDPVNVTVAWFFDGGHCKVPLVHREYYGCPGDAMPSVETCTGGYSYTRTRIDTLMEYALVNAS : 144
HLJ8        : PPLDVSSRSPDPVNVTVAWFFDGGHCKVPLVHREYYGCPGDAMPSVETCTGGYSYTRTRIDTLMEYALVNAS : 144
HN1201      : PPLDVSSRSPDPVNVTVAWFFDGGHCKVPLVHREYYGCPGDAMPSVETCTGGYSYTRTRIDTLMEYALVNAS : 144
HNB         : PPLDVSSRSPDPVNVTVAWFFDGGHCKVPLVHREYYGCPGDAMPSVETCTGGYSYTRTRIDTLMEYALVNAS : 144
HNX         : PPLDVSSRSPDPVNVTVAWFFDGGHCKVPLVHREYYGCPGDAMPSVETCTGGYSYTRTRIDTLMEYALVNAS : 144
JS-2012     : PPLDVSSRSPDPVNVTVAWFFDGGHCKVPLVHREYYGCPGDAMPSVETCTGGYSYTRTRIDTLMEYALVNAS : 144
TJ          : PPLDVSSRSPDPVNVTVAWFFDGGHCKVPLVHREYYGCPGDAMPSVETCTGGYSYTRTRIDTLMEYALVNAS : 144
Ea          : PPLDVSSRSSDPVNVTVAWFFDGGHCKVPLVHREYYGCPGDAMPSVETCTGGYSYTRTRIDTLMEYALVNAS : 144
Fa          : PPLDVSSRSSDPVNVTVAWFFDGGHCKVPLVHREYYGCPGDAMPSVETCTGGYSYTRTRIDTLMEYALVNAS : 144
SC          : PPLDVSSRSSDPVNVTVAWFFDGGHCKVPLVHREYYGCPGDAMPSVETCTGGYSYTRTRIDTLMEYALVNAS : 144
Bartha-K61  : PPLDVSSRSSDPVNVTVAWFFDGGHCKVPLVHREYYGCPGDAMPSVETCTGGYSYTRTRIDTLMEYALVNAS : 144
              PPLDVSSRS DPVNVTVAWFFDGGHCKVPLVHREYYGCPGDAMPSVETCTGGYSYTRTRIDTLMEYALVNAS
```

```
                     *         160         *         180         *         200         *
HeN21      : LVLQPGLYDAGLYIVVLVFGDDAYLGTVSLSVEANLDYPCGMKHGLTITRPGATLPPIAPTAGDHQRWRGCF : 216
HuB20      : LVLQPGLYDAGLYIVVLVFGDDAYLGTVSLSVEANLDYPCGMKHGLTITRPGATLPPIAPTAGDHQRWRGCF : 216
hSD-1      : LVLQPGLYDAGLYIVVLVFGDDAYLGTVSLSVEANLDYPCGMKHGLTITRPGATLPPIAPTAGDHQRWRGCF : 216
HeN1       : LVLQPGLYDAGLYIVVLVFGDDAYLGTVSLSVEANLDYPCGMKHGLTITRPGATLPPIAPTAGDHQRWRGCF : 216
HLJ8       : LVLQPGLYDAGLYIVVLVFGDDAYLGTVSLSVEANLDYPCGMKHGLTITRPGATLPPIAPTAGDHQRWRGCF : 216
HN1201     : LVLQPGLYDAGLYIVVLVFGDDAYLGTVSLSVEANLDYPCGMKHGLTITRPGATLPPIAPTAGDHQRWRGCF : 216
HNB        : LVLQPGLYDAGLYIVVLVFGDDAYLGTVSLSVEANLDYPCGMKHGLTITRPGATLPPIAPTAGDHQRWRGCF : 216
HNX        : LVLQPGLYDAGLYIVVLVFGDDAYLGTVSLSVEANLDYPCGMKHGLTITRPGATLPPIAPTAGDHQRWRGCF : 216
JS-2012    : LVLQPGLYDAGLYIVVLVFGDDAYLGTVSLSVEANLDYPCGMKHGLTITRPGATLPPIAPTAGDHQRWRGCF : 216
TJ         : LVLQPGLYDAGLYIVVLVFGDDAYLGTVSLSVEANLDYPCGMKHGLTITRPGATLPPIAPTAGDHQRWRGCF : 216
Ea         : LVLQPGLYDAGLYIVVLVFGDDAYLGTVSLSVEANLDYPCGMKHGLTITRPGATLPPIAPTAGDHQRWRGCF : 216
Fa         : LVLQPGLYDAGLYIVVLVFGDDAYLGTVSLSVEANLDYPCGMKHGLTITRPGATLPPIAPTAGDHQRWRGCF : 216
SC         : LVLQPGLYDAGLYIVVLVFGDDAYLGTVSLSVEANLDYPCGMKHGLTITRPGATLPPIAPTAGDHQRWRGCF : 216
Bartha-K61 : LVLQPGLYDAGLYIVVLVFGDDAYLGTVSLSVEANLDYPCGMKHGLTITRPGATLPPIAPTAGDHQRWRGCF : 216
             LVLQPGLYDAGLYIVVLVFGDDAYLGTVSLSVEANLDYPCGMKHGLTITRPGATLPPIAPTAGDHQRWRGCF

              220         *         240         *         260         *         280
HeN21      : PSTDEGAWENVTAAEKGLSDDYADYYDVHIFRLESDDEVVHGDAPEAPEGEEVTEEEAELTSSDLDNIEIEV : 288
HuB20      : PSTDEGAWENVTAAEKGLSDDYADYYDVHIFRLESDDEVVHGDAPEAPEGEEVTEEEAELTSSDLDNIEIEV : 288
hSD-1      : PSTDEGAWENVTAAEKGLSDDYADYYDVHIFRLESDDEVVHGDAPEAPEGEEVTEEEAELTSSDLDNIEIEV : 288
HeN1       : PSTDEGAWENVTAAEKGLSDDYADYYDVHIFRLESDDEVVHGDAPEAPEGEEVTEEEAELTSSDLDNIEIEV : 288
HLJ8       : PSTDEGAWENVTAAEKGLSDDYADYYDVHIFRLESDDEVVHGDAPEAPEGEEVTEEEAELTSSDLDNIEIEV : 288
HN1201     : PSTDESAWENVTAAEKGLSDDYADYYDVHIFRLESDDEVVHGDAPEAPEGEEVTEEEAELTSSDLDNIEIEV : 288
HNB        : PSTDEGAWENVTAAEKGLSDDYADYYDVHIFRLESDDEVVHGDAPEAPEGEEVTEEEAELTSSDLDNIEIEV : 288
HNX        : PSTDEGAWENVTAAEKGLSDDYADYYDVHIFRLESDDEVVHGDAPEAPEGEEVTEEEAELTSSDLDNIEIEV : 288
JS-2012    : PSTDEGAWENVTAAEKGLSDDYADYYDVHIFRLESDDEVVHGDAPEAPEGEEVTEEEAELTSSDLDNIEIEV : 288
TJ         : PSTDEGAWENVTAAEKGLSDDYADYYDVHIFRLESDDEVVHGDAPEAPEGEEVTEEEAELTSSDLDNIEIEV : 288
Ea         : PSTDEGAWENVTAAEKGLSDDYADYYDVHIFRLESDDEVVHGDAPEAPEGEEVTEEEAELTSSDLDNIEIEV : 288
Fa         : PSTDEGAWENVTAAEKGLSDDYADYYDVHIFRLESDDEVVHGDAPEAPEGEEVTEEEAELTSSDLDNIEIEV : 288
SC         : PSTDEGAWENVTAAEKGLSDDYADYYDVHIFRLESDDEVVHGDAPEAPEGEEVTEEEAELTSSDLDNIEIEV : 288
Bartha-K61 : PSTDEGAWENVTAAEKGLSDDYADYYDAHIFRLESDDEVVHGDAPEAPEGEEVTEEEAELTSSDLDNIEIEV : 288
             PSTDEgAWENVTAAEKGLSDDYADYYDvHIFRLESDDEVVHGDAPEAPEGEEVTEEEAELTSSDLDNIEIEV
```

```
                     *        300         *        320         *        340         *        360
HeN21       : VGSPAAPVEGAGDGEEGHGDEEDEELTSSDLDNIEIEVVGSPAAARFFAASTTPRAPTRAAEITTMTTVTTV : 360
HuB20       : VGSPAAPVEGAGDGEEGHGDEEDEELTSSDLDNIEIEVVGSPAAARFFAASTTPRAPTRATEITTMTTVTTV : 360
hSD-1       : VGSPAAPVEGAGDGEEGHGDEEDEELTSSDLDNIEIEVVGSPAAARFFAASTTPRAPTRAAEITTMTTVTTV : 360
HeN1        : VGSPAAPVEGAGDGEEGHGDEEDEELTSSDLDNIEIEVVGSPAAARFFAASTTPRAPTRAAEITTMTTVTTV : 360
HLJ8        : VGSPAAPVEGAGDGEEGHGDEEDEELTSSDLDNIEIEVVGSPAAARFFAASTTPRAPTRAAEITTMTTVTTV : 360
HN1201      : VGSPAAPVEGAGDGEEGHGDEEDEELTSSDLDNIEIEVVGSPAAARFFAASTTPRAPTRAAEITTMTTVTTV : 360
HNB         : VGSPAAPVEGAGDGEEGHGDEEDEELTSSDLDNIEIEVVGSPAAARFFAASTTPRAPTRAAEITTMTTVTTV : 360
HNX         : VGSPAAPVEGAGDGEEGHGDEEDEELTSSDLDNIEIEVVGSPAAARFFAASTTPRAPTRAAEITTMTTVTTV : 360
JS-2012     : VGSPAAPVEGAGDGEEGHGDEEDEELTSSDLDNIEIEVVGSPAAARFFAASTTPRAPTRAAEITTMTTVTTV : 360
TJ          : VGSPAAPVEGAGDGEEGHGDEEDEELTSSDLDNIEIEVVGSPAAARFFAASTTPRAPTRAAEITTMTTVTTV : 360
Ea          : VGSPAAPVEGAGDGEEGHGDEEDEELTSSDLDNIEIEVVGSPAAARFFAASTTPRAPTRAAEITTMTTVTTV : 360
Fa          : VGSPAAPVEGAGDGEEGHGDEEDEELTSSDLDNIEIEVVGSPAAARFFAASTTPRAPTRAAEITTMTTVTTV : 360
SC          : VGSPAAPVEGAGDGEEGHGDEEDEELTSSDLDNIEIEVVGSPAAARFFAASTTPRAPTRAAEITTMTTVTTV : 360
Bartha-K61  : VGSPAAPVEGAGDGEEGHRDEEDEELTSSDLDNIEIEVVGSPAAARFFAASTTPRAPTRAAEITTMTTVTTV : 360
              VGSPAAPVEGAGDGEEGHgDEEDEELTSSDLDNIEIEVVGSPAAARFFAASTTPRAPTRAaEITTMTTVTTV

                         *        380         *        400         *        420         *
HeN21       : RTTEDPSGITDCRRSDFVSPSDIFVTPTGSPALLLGFLGSALASRPLHLTAGETAQHVREAQQKSRHVRSLG : 432
HuB20       : RTTEDPSGITDCRRSDFVSPSDIFVTPTGSPALLLGFLGSALASRPLHLTAGETAQHVREAQQKSRHVRSLG : 432
hSD-1       : RTTEDPSGITDCRRSDFVSPSDIFVTPTGSPALLLGFLGSALASRPLHLTAGETAQHVREAQQKSRHVRSLG : 432
HeN1        : RTTEDPSGITDCRRSDFVSPSDIFVTPTGSPALLLGFLGSALASRPLHLTAGETAQHVREAQQKSRHVRSLG : 432
HLJ8        : RTTEDPSGITDCRRSDFVSPSDIFVTPTGSPALLLGFLGSALASRPLHLTAGETAQHVREAQQKSRHVRSLG : 432
HN1201      : RTTEDPSGITDCRRSDFVSPSDIFVTPTGSPALLLGFLGSALASRPLHLTAGETAQHVREAQQKSRHVRSLG : 432
HNB         : RTTEDPSGITDCRRSDFVSPSDIFVTPTGSPALLLGFLGSALASRPLHLTAGETAQHVREAQQKSRHVRSLG : 432
HNX         : RTTEDPSGITDCRRSDFVSPSDIFVTPTGSPALLLGFLGSALASRPLHLTAGETAQHVREAQQKSRHVRSLG : 432
JS-2012     : RTTEDPSGITDCRRSDFVSPSDIFVTPTGSPALLLGFLGSALASRPLHLTAGETAQHVREAQQKSRHVRSLG : 432
TJ          : RTTEDPSGITDCRRSDFVSPSDIFVTPTGSPALLLGFLGSALASRPLHLTAGETAQHVREAQQKSRHVRSLG : 432
Ea          : RTTEDPSGITDCRRSDFVSPSDIFVTPTGSPALLLGFLGSALASRPLHLTAGETAQHVREAQQKSRHVRSLG : 432
Fa          : RTTEDPSGITDCRRSDFVSPSDIFVTPTGSPALLLGFLGSALASRPLHLTAGETAQHVREAQQKSRHVRSLG : 432
SC          : RTTEDPSGITDCRRSDFVSPSDIFVTPTGSPALLLGFLGSALASRPLHLTAGETAQHVREAQQKSRHVRSLG : 432
Bartha-K61  : RTTEDPSGITDCRRSDFVSPSDIFVTPTGSPALLLGFLGSALASRPLHLTAGETAQHVREAQQKSRHIRSLG : 432
              RTTEDPSGITDCRRSDFVSPSDIFVTPTGSPALLLGFLGSALASRPLHLTAGETAQHVREAQQKSRH6RSLG
```

```
                        440         *        460         *        480         *        500
HeN21       : GLQLSVETETTNTTTTQTGLSGDIRTSIYICVALAGLVVVGIVIMCLHMAIIRARARNDGYRHVASA* : 499
HuB20       : GLQLSVETETTNTTTTQTGLSGDIRTSIYICVALAGLVVVGIVIMCLHMAIIRARARNDGYRHVASA* : 499
hSD-1       : GLQLSVETETTNTTTTQTGLSGDIRTSIYICVALAGLVVVGIVIMCLHMAIIRARARNDGYRHVASA- : 499
HeN1        : GLQLSVETETTNTTTTQTGLSGDIRTSIYICVALAGLVVVGIVIMCLHMAIIRARARNDGYRHVASA- : 499
HLJ8        : GLQLSVETETTNTTTTQTGLSGDIRTSIYICVALAGLVVVGIVIMCLHMAIIRARARNDGYRHVASA- : 499
HN1201      : GLQLSVETETTNTTTTQTGLSGDIRTSIYICVALAGLVVVGIVIMCLHMAIIRARARNDGYRHVASA- : 499
HNB         : GLQLSVETETTNTTTTQTGLSGDIRTSIYICVALAGLVVVGIVIMCLHMAIIRARARNDGYRHVASA- : 499
HNX         : GLQLSVETETTNTTTTQTGLSGDIRTSIYICVALAGLVVVGIVIMCLHMAIIRARARNDGYRHVASA- : 499
JS-2012     : GLQLSVETETTNTTTTQTGLSGDIRTSIYICVALAGLVVVGIVIMCLHMAIIRARARNDGYRHVASA- : 499
TJ          : GLQLSVETETTNTTTTQTGLSGDIRTSIYICVALAGLVVVGIVIMCLHMAIIRARARNDGYRHVASA- : 499
Ea          : GLQLSVETETTNTTTTQTGLSGDIRTSIYICVALAGLVVVGIVIMCLHMAIIRARARNDGYRHVASA- : 499
Fa          : GLQLSVETETTNTTTTQTGLSGDIRTSIYICVALAGLVVVGIVIMCLHMAIIRARARNDGYRHVASA- : 499
SC          : GLQLSVETETTNTTTTQTGLSGDIRTSIYICVALAGLVVVGIVIMCLHMAIIRARARNDGYRHVASA- : 499
Bartha-K61  : GLQLSVETETTNTTTTQTGLSGDIRTSIYICVALAGLVVVGIVIMCLHMAITRARARNDGYRHVASA* : 499
              GLQLSVETETTNTTTTQTGLSGDIRTSIYICVALAGLVVVGIVIMCLHMAIiRARARNDGYRHVASA
```

## gH

```
                            *        20         *        40         *        60         *
HeN21       : MPASSVRLPLRLLTLAGLLALAGAAALARGAPQGGPPSPQGGPAPTAAPARGPTLFVLDGDGSAWFVFQLGG :  72
HuB20       : MPASSVRLPLRLLTLAGLLALAGAAALARGAPQGGPPSPQGGPAPTAAPARGPTLFVLVGDGSAWFVFQLGG :  72
hSD-1       : MPASSVRLPLRLLTLAGLLALAGAAALARGAPQGGPPSPQGGPAPTAAPARGPTLFVLDGDGSAWFVFQLGG :  72
HeN1        : MPASSVRLPLRLLTLAGLLALAGAAALARGAPQGGPPSPQGGPAPTAAPARGPTLFVLDGDGSAWFVFQLGG :  72
HLJ8        : MPASSVRLPLRLLTLAGLLALAGAAALARGAPQGGPPSPQGGPAPTAAPARGPTLFVLDGDGSAWFVFQLGG :  72
HN1201      : MPASSVRLPLRLLTLAGLLALAGAAALARGAPQGGPPSPQGGPAPTAAPARGPTLFVLDGDGSAWFVFQLGG :  72
HNB         : MPASSVRLPLRLLTLAGLLALAGAAALARGAPQGGPPSPQGGPAPTAAPARGPTLFVLDGDGSAWFVFQLGG :  72
HNX         : MPASSVRLPLRLLTLAGLLALAGAAALARGAPQGGPPSPQGGPAPTAAPARGPTLFVLDGDGSAWFVFQLGG :  72
JS-2012     : MPASSVRLPLRLLTLAGLLALAGAAALARGAPQGGPPSPQGGPAPTAAPARGPTLFVLDGDGSAWFVFQLGG :  72
TJ          : MPASSVRLPLRLLTLAGLLALAGAAALARGAPQGGPPSPQGGPAPTAAPARGPTLFVLDGDGSAWFVFQLGG :  72
Ea          : MPASSVRLPLRLLTLAGLLALAGAAALARGAPQGGPPSPQGGPAPTAAPARGPTLFVLVGDGSAWFVFQLGG :  72
Fa          : MPASSVRLPLRLLTLAGLLALAGAAALARGAPQGGPPSPQGGPAPTAAPARGPTLFVLVGDGSAWFVFQLGG :  72
SC          : MPASSVRLPLRLLTLAGLLALAGAAALARGAPQGGPPSPQGGPAPTAAPARGPTLFVLDGDGSAWFVFQLGG :  72
Bartha-K61  : MPASSVRLPLRLLTLAGLLALAGAAALARGAPQGGPPSPQGGPAPTAAPARGPTLFVLDGDGSAWFVFQLGG :  72
              MPASSVRLPLRLLTLAGLLALAGAAALARGAPQGGPPSPQGGPAPTAAPARGPTLFVLdGDGSAWFVFQLGG

                     80         *       100         *       120         *       140
HeN21       : LGALNDTRIRGHLLGRYLVSYQVVPPPVSAWYFVQRPRERPRLSGPPSGAELVAFDAPGVRRTYTTAAVWPA : 144
HuB20       : LGALNDTRIRGHLLGRYLVSYQVVPPPVSAWYFVQRPRERPRLSGPPSGAELVAFDAPGVRRTYTTAAVWPA : 144
hSD-1       : LGALNDTRIRGHLLGRYLVSYQVVPPPVSAWYFVQRPRERPRLSGPPSGAELVAFDAPGVRRTYTTAAVWPA : 144
HeN1        : LGALNDTRIRGHLLGRYLVSYQVVPPPVSAWYFVQRPRERPRLSGPPSGAELVAFDAPGVRRTYTTAAVWPA : 144
HLJ8        : LGALNDTRIRGHLLGRYLVSYQVVPPPVSAWYFVQRPRERPRLSGPPSGAELVAFDAPGVRRTYTTAAVWPA : 144
HN1201      : LGALNDTRIRGHLLGRYLVSYQVVPPPVSAWYFVQRPRERPRLSGPPSGAELVAFDAPGVRRTYTTAAVWPA : 144
HNB         : LGALNDTRIRGHLLGRYLVSYQVVPPPVSAWYFVQRPRERPRLSGPPSGAELVAFDAPGVRRTYTTAAVWPA : 144
HNX         : LGALNDTRIRGHLLGRYLVSYQVVPPPVSAWYFVQRPRERPRLSGPPSGAELVAFDAPGVRRTYTTAAVWPA : 144
JS-2012     : LGALNDTRIRGHLLGRYLVSYQVVPPPVSAWYFVQRPRERPRLSGPPSGAELVAFDAPGVRRTYTTAAVWPA : 144
TJ          : LGALNDTRIRGHLLGRYLVSYQVVPPPVSAWYFVQRPRERPRLSGPPSGAELVAFDAPGVWRTYTTAAVWPA : 144
Ea          : LGALNDTRIRGHLLGRYLVSYQVVPPPVSAWYFVQRPRERPRLSGPPSGAELVAFDAPGVRRTYTTAAVWPA : 144
Fa          : LGALNDTRIRGHLLGRYLVSYQVVPPPVSAWYFVQRPRERPRLSGPPSGAELVAFDAPGVRRTYTTAAVWPA : 144
SC          : LGALNDTRIRGHLLGRYLVSYQVVPPPVSAWYFVQRPRERPRLSGPPSGAELVAFDAPGVRRTYTTAAVWPA : 144
Bartha-K61  : LGALNDTRIRGHLLGRYLVSYQVVPPPVSAWYFVQRPRERPRLSGPPSGAELVAFDAPGVRRTYTTAAVWPA : 144
              LGALNDTRIRGHLLGRYLVSYQVVPPPVSAWYFVQRPRERPRLSGPPSGAELVAFDAPGVrRTYTTAAVWPA
```

```
                  *        160        *        180        *        200        *
HeN21      : EVAVLADAEARCPAAVFNVTLGEAFLGLRVALRSFLPLEVIISAERMRMIAPPALGSDLEPPGPPAGRFHVY : 216
HuB20      : EVAVLADAEARCPAAVFNVTLGEAFLGLRVALRSFLPLEVIISAERMRMIAPPALGSDLEPPGPPAGRFHVY : 216
hSD-1      : EVAVLADAEARCPAAVFNVTLGEAFLGLRVALRSFLPLEVIISAERMRMIAPPALGSDLEPPGPPAGRFHVY : 216
HeN1       : EVAVLADAEARCPAAVFNVTLGEAFLGLRVALRSFLPLEVIISAERMRMIAPPALGSDLEPPGPPAGRFHVY : 216
HLJ8       : EVAVLADAEARCPAAVFNVTLGEAFLGLRVALRSFLPLEVIISAERMRMIAPPALGSDLEPPGPPAGRFHVY : 216
HN1201     : EVAVLADAEARCPAAVFNVTLGEAFLGLRVALRSFLPLEVIISAERMRMIAPPALGSDLEPPGPPAGRFHVY : 216
HNB        : EVAVLADAEARCPAAVFNVTLGEAFLGLRVALRSFLPLEVIISAERMRMIAPPALGSDLEPPGPPAGRFHVY : 216
HNX        : EVAVLADAEARCPAAVFNVTLGEAFLGLRVALRSFLPLEVIISAERMRMIAPPALGSDLEPPGPPAGRFHVY : 216
JS-2012    : EVAVLADAEARCPAAVFNVTLGEAFLGLRVALRSFLPLEVIISAERMRMIAPPALGSDLEPPGPPAGRFHVY : 216
TJ         : EVAVLADAEARCPAAVFNVTLGEAFLGLRVALRSFLPLEVIISAERMRMIAPPALGSDLEPPGPPAGRFHVY : 216
Ea         : EVAVLADAEARCPAAVFNVTLGEAFLGLRVALRSFLPLEVIISAERMRMIAPPALGSDLEPPGPPAGRFHVY : 216
Fa         : EVAVLADAEARCPAAVFNVTLGEAFLGLRVALRSFLPLEVIISAERMRMIAPPALGSDLEPPGPPAGRFHVY : 216
SC         : EVAVLADAEARCPAAVFNVTLGEAFLGLRVALRSFLPLEVIISAERMRMIAPPALGSDLEPPGPPAGRFHVY : 216
Bartha-K61 : EVAVLADAEARCPAAVFNVTLGEAFLGLRVALRSFLPLEVIISAERMRMIAPPALGSDLEPPGPPAGRFHVY : 216
             EVAVLADAEARCPAAVFNVTLGEAFLGLRVALRSFLPLEVIISAERMRMIAPPALGSDLEPPGPPAGRFHVY

               220        *        240        *        260        *        280
HeN21      : TLGFLSDGAMHQTMRDVAAYVHESDDYLAQLSAAHAAALAAVVQPGPYYFYRAAVRLGVAAFVFSEAARRDR : 288
HuB20      : TLGFLSDGAMHQTMRDVAAYVHESDDYLAQLSAAHAAALAAVVQPGPYYFYRAAVRLGVAAFVFSEAARRDR : 288
hSD-1      : TLGFLSDGAMHQTMRDVAAYVHESDDYLAQLSAAHAAALAAVVQPGPYYFYRAAVRLGVAAFVFSEAARRDR : 288
HeN1       : TLGLLSDGAMHQTMRDVAAYVHESDDYLAQLSAARAAALAAVVQPGPYYFYRAAVRLGVAAFVFSEAARRDR : 288
HLJ8       : TLGFLSDGAMHQTMRDVAAYVHESDDYLAQLSAAHAAALAAVVQPGPYYFYRAAVRLGVAAFVFSEAARRDR : 288
HN1201     : TLGFLSDGAMHQTMRDVAAYVHESDDYLAQLSAAHAAALAAVVQPGPYYFYRAAVRLGVAAFVFSEAARRDR : 288
HNB        : TLGFLSDGAMHQTMRDVAAYVHESDDYLAQLSAAHAAALAAVVQPGPYYFYRAAVRLGVAAFVFSEAARRDR : 288
HNX        : TLGLLSDGAMHQTMRDVAAYVHESDDYLAQLSAARAAALAAVVQPGPYYFYRAAVRLGVAAFVFSEAARRDR : 288
JS-2012    : TLGFLSDGAMHQTMRDVAAYVHESDDYLAQLSAAHAAALAAVVQPGPYYFYRAAVRLGVAAFVFSEAARRDR : 288
TJ         : TLGFLSDGAMHQTMRDVAAYVHESDDYLAQLSAAHAAALAAVVQPGPYYFYRAAVRLGVAAFVFSEAARRDR : 288
Ea         : TLGFLSDGAMHQTMRDVAAYVHESDDYLAQLSAAHAAALAAVVQPGPYYFYRAAVRLGVAAFVFSEAARRDR : 288
Fa         : TLGFLSDGAMHQTMRDVAAYVHESDDYLAQLSAAHAAALAAVVQPGPYYFYRAAVRLGVAAFVFSEAARRDR : 288
SC         : TLGFLSDGAMHQTMRDVAAYVHESDDYLAQLSAAHAAALAAVVQPGPYYFYRAAVRLGVAAFVFSEAARRDR : 288
Bartha-K61 : TLGFLSDGAMHQTMRDVAAYVHESDDYLAQLSAAHAAALAAVVQPGPYYFYRAAVRLGVAAFVFSEAARRDR : 288
             TLGfLSDGAMHQTMRDVAAYVHESDDYLAQLSAAhAAALAAVVQPGPYYFYRAAVRLGVAAFVFSEAARRDR
```

```
                      *        300         *        320         *        340         *        360
HeN21      : RASAPALLRVESDARLLSRLLMRAAGCPAGFAGLFDGRAERVPVAPADQLRAAWTFGEDPAPRLDLARATVA : 360
HuB20      : RASAPALLRVESDARLLSRLLMRAAGCPAGFAGLFDGRAERVPVAPADQLRAAWTFGEDPAPRLDLARATVA : 360
hSD-1      : RASAPALLRVESDARLLSRLLMRAAGCPAGFAGLFDGRAERVPVAPADQLRAAWTFGEDPAPRLDLARATVA : 360
HeN1       : RASAPALLRVESDARLLSRLLMRAAGCPASFAGLFDGRAERVPVAPADQLRAAWTFGEDPAPRLDLARATVA : 360
HLJ8       : RASAPALLRVESDARLLSRLLMRAAGCPAGFAGLFDGRAERVPVAPADQLRAAWTFGEDPAPRLDLARATVA : 360
HN1201     : RASAPALLRVESDARLLSRLLMRAAGCPAGFAGLFDGRAERVPVAPADQLRAAWTFGEDPAPRLDLARATVA : 360
HNB        : RASAPALLRVESDARLLSRLLMRAAGCPAGFAGLFDGRAERVPVAPADQLRAAWTFGEDPAPRLDLARATVA : 360
HNX        : RASAPALLRVESDARLLSRLLMRAAGCPASFAGLFDGRAERVPVAPADQLRAAWTFGEDPAPRLDLARATVA : 360
JS-2012    : RASAPALLRVESDARLLSRLLMRAAGCPAGFAGLFDGRAERVPVAPADQLRAAWTFGEDPAPRLDLARATVA : 360
TJ         : RASAPALLRVESDARLLSRLLMRAAGCPAGFAGLFDGRAERVPVAPADQLRAAWTFGEDPAPRLDLARATVA : 360
Ea         : RASAPALLRVESDARLLSRLLMRAAGCPAGFAGLFDGRAERVPVAPADQLRAAWTFGEDPAPRLDLARATVA : 360
Fa         : RASAPALLRVESDARLLSRLLMRAAGCPAGFAGLFDGRAERVPVAPADQLRAAWTFGEDPAPRLDLARATVA : 360
SC         : RASAPALLRVESDARLLSRLLMRAAGCPAGFAGLFDGRAERVPVAPADQLRAAWTFGEDPAPRLDLARATVA : 360
Bartha-K61 : RASAPALLRVESDARLLSRLLMRAAGCPAGFAGLFDGRAERVPVAPADQLRAAWTFGEDPAPRLDLARATVA : 360
             RASAPALLRVESDARLLSRLLMRAAGCPAgFAGLFDGRAERVPVAPADQLRAAWTFGEDPAPRLDLARATVA

                      *        380         *        400         *        420         *
HeN21      : EAYRRSVRGKPFDQQALFFAVALLLRAGGPGDARETLLRTTAMCTAERAAAAAELTRAALSPTAAWNEPFSL : 432
HuB20      : EAYRRSVRGKPFDQQALFFAVALLLRAGGPGDARETLLRTTAMCTAERAAAAAELTRAALSPTAAWNEPFSL : 432
hSD-1      : EAYRRSVRGKPFDQQALFFAVALLLRAGGPGDARETLLRTTAMCTAERAAAAAELTRAALSPTAAWNEPFSL : 432
HeN1       : EAYRRSVRGKPFDQQALFFAVALLLRAGGPGDARETLLRTTAMCTAERAAAAAELTRAALSPTAAWNEPFSL : 432
HLJ8       : EAYRRSVRGKPFDQQALFFAVALLLRAGGPGDARETLLRTTAMCTAERAAAAAELTRAALSPTAAWNEPFSL : 432
HN1201     : EAYRRSVRGKPFDQQALFFAVALLLRAGGPGDARETLLRTTAMCTAERAAAAAELTRAALSPTAAWNEPFSL : 432
HNB        : EAYRRSVRGKPFDQQALFFAVALLLRAGGPGDARETLLRTTAMCTAERAAAAAELTRAALSPTAAWNEPFSL : 432
HNX        : EAYRRSVRGKPFDQQALFFAVALLLRAGGPGDARETLLRTTAMCTAERAAAAAELTRAALSPTAAWNEPFSL : 432
JS-2012    : EAYRRSVRGKPFDQQALFFAVALLLRAGGPGDARETLLRTTAMCTAERAAAAAELTRAALSPTAAWNEPFSL : 432
TJ         : EAYRRSVRGKPFDQQALFFAVALLLRAGGPGDARETLLRTTAMCTAERAAAAAELTRAALSPTAAWNEPFSL : 432
Ea         : EAYRRSVRGKPFDQQALFFAVALLLRAGGPGDARETLLRTTAMCTAERAAAAAELTRAALSPTAAWNEPFSL : 432
Fa         : EAYRRSVRGKPFDQQALFFAVALLLRAGGPGDARETLLRTTAMCTAERAAAAAELTRAALSPTAAWNEPFSL : 432
SC         : EAYRRSVRGKPFDQQALFFAVALLLRAGGPGDARETLLRTTAMCTAERAAAAAELTRAALSPTAAWNEPFSL : 432
Bartha-K61 : EAYRRSVRGKPFDQQALFFAVALLLRAGGPGDARETLLRTTAMCTAERAAAAAELTRAALSPTAAWNEPFSL : 432
             EAYRRSVRGKPFDQQALFFAVALLLRAGGPGDARETLLRTTAMCTAERAAAAAELTRAALSPTAAWNEPFSL
```

```
                       440         *       460         *       480         *       500
HeN21      : LDGLSPCAVSLRRDLGGDATLANLGAAARLALAPAGAPGAAAATDEGAEEEE-DPVARAAPEIPAEALLALP : 503
HuB20      : LDVLSPCAVSLRRDLGGDATLANLGAAARLALAPAGAPGAAAATDEGAEEEE-DPVARAAPEIPAEALLALP : 503
hSD-1      : LDVLSPCAVSLRRDLGGDATLANLGAAARLALAPAGAPGAAAATDEGAEEEE-DPVARAAPEIPAEALLALP : 503
HeN1       : LDGLSPCAVSLRRDLGGDATLANLGAAARLALAPAGAPGAAAATDEGAEEEEEDPVARAAPEIPAEALLALP : 504
HLJ8       : LDVLSPCAVSLRRDLGGDATLANLGAAARLALAPAGAPGAAAATDEGAEEEE-DPVARAAPEIPAEALLALP : 503
HN1201     : LDVLSPCAVSLRRDLGGDATLANLGAAARLALAPAGAPGAAAATDEGAEEEE-DPVARAAPEIPAEALLALP : 503
HNB        : LDVLSPCAVSLRRDLGGDATLANLGAAARLALAPAGAPGAAAATDEGAEEEE-DPVARAAPEIPAEALLALP : 503
HNX        : LDGLSPCAVSLRRDLGGDATLANLGAAARLALAPAGAPGAAAATDEGAEEEEEDPVARAAPEIPAEALLALP : 504
JS-2012    : PDVLSPCAVSLRRDLGGDATLANLGAAARLALAPAGAPGAAAATDEGAEEEE-DPVARAAPEIPAEALLALP : 503
TJ         : LDVLSPCAVSLRRDLGGDATLANLGAAARLALAPAGAPGAAAATDEGAEEEE-DPVARAAPEIPAEALLALP : 503
Ea         : LDVLSPCAVSLRRDLGGDATLANLGAAARLALAPAGAPGAAAATDEGAEEEE-DPVARAAPEIPAEALLALP : 503
Fa         : LDVLSPCAVSLRRDLGGDATLANLGAAARLALAPAGAPGAAAATDEGAEEEE-DPVARAAPEIPAEALLALP : 503
SC         : LDVLSPCAVSLRRDLGGDATLANLGAAARLALAPAGAPGAAAATDEGAEEEE-DPVARAAPEIPAEALLALP : 503
Bartha-K61 : LDVLSSCAVSLRRDLGGDATLANLGAAARLALAPAGAPGAAAATDEGAGEEE-DPVARAAPEIPAEALLALP : 503
             lDvLSpCAVSLRRDLGGDATLANLGAAARLALAPAGAPGAAAATDEGAeEEE DPVARAAPEIPAEALLALP

                   *       520         *       540         *       560         *
HeN21      : LRGGASFVFTRRRPDCGPAYTLGGVDIANPLVLALVSNDSAACDYTDRMPESQHLPATDNPSVCVYCDCVFV : 575
HuB20      : LRGGASFVFTRRRPDCGPAYTLGGVDIANPLVLALVSNDSAACDYTDRMPESQHLPATDNPSVCVYCDCVFV : 575
hSD-1      : LRGGASFVFTRRRPDCGPAYTLGGVDIANPLVLALVSNDSAACDYTDRMPESQHLPATDNPSVCVYCDCVFV : 575
HeN1       : LRGGASFVFTRRRPDCGPAYTLGGVDIANPLVLALVSNDSAACDYTDRMPESQHLPATDNPSVCVYCDCVFV : 576
HLJ8       : LRGGASFVFTRRRPDCGPAYTLGGVDIANPLVLALVSNDSAACDYTDRMPESQHLPATDNPSVCVYCDCVFV : 575
HN1201     : LRGGASFVFTRRRPDCGPAYTLGGVDIANPLVLALVSNDSAACDYTDRMPESQHLPATDNPSVCVYCDCVFV : 575
HNB        : LRGGASFVFTRRRPDCGPAYTLGGVDIANPLVLALVSNDSAACDYTDRMPESQHLPATDNPSVCVYCDCVFV : 575
HNX        : LRGGASFVFTRRRPDCGPAYTLGGVDIANPLVLALVSNDSAACDYTDRMPESQHLPATDNPSVCVYCDCVFV : 576
JS-2012    : LRGGASFVFTRRRPDCGPAYTLGGVDIANPLVLALVSNDSAACDYTDRMPESQHLPATDNPSVCVYCDCVFV : 575
TJ         : LRGGASFVFTRRRPDCGPAYTLGGVDIANPLVLALVSNDSAACDYTDRMPESQHLPATDNPSVCVYCDCVFV : 575
Ea         : LRGGASFVFTRRRPDCGPAYTLGGVDIANPLVLALVSNDSAACDYTDRMPESQHLPATDNPSVCVYCDCVFV : 575
Fa         : LRGGASFVFTRRRPDCGPAYTLGGVDIANPLVLALVSNDSAACDYTDRMPESQHLPATDNPSVCVYCDCVFV : 575
SC         : LRGGASFVFTRRRPDCGPAYTLGGVDIANPLVLALVSNDSAACDYTDRMPESQHLPATDNPSVCVYCDCVFV : 575
Bartha-K61 : LRGGASFVFTRRRPDCGPAYTLGGVDIANPLVLALVSNDSAACDYTDRMPESQHLPATDNPSVCVYCDCVFV : 575
             LRGGASFVFTRRRPDCGPAYTLGGVDIANPLVLALVSNDSAACDYTDRMPESQHLPATDNPSVCVYCDCVFV
```

```
                 580         *        600         *        620         *        640
HeN21       : RYSSAGTILETVLIESKDMEEQLMAGANSTIPSFNPTLHGGDVKALMLFPNGTVVDLLSFTSTRLAPVSPAY : 647
HuB20       : RYSSAGTILETVLIESKDMEEQLMAGANSTIPSFNPTLHGGDVKALMLFPNGTVVDLLSFTSARLAPVSPAY : 647
hSD-1       : RYSSAGTILETVLIESKDMEEQLMAGANSTIPSFNPTLHGGDVKALMLFPNGTVVDLLSFTSTRLAPVSPAY : 647
HeN1        : RYSSAGTILETVLIESKDMEEQLMAGANSTIPSFNPTLHGGDVKALMLFPNGTVVDLLSFTSTRLAPVSPAY : 648
HLJ8        : RYSSAGTILETVLIESKDMEEQLMAGANSTIPSFNPTLHGGDVKALMLFPNGTVVDLLSFTSTRLAPVSPAY : 647
HN1201      : RYSSAGTILETVLIESKDMEEQLMAGANSTIPSFNPTLHGGDVKALMLFPNGTVVDLLSFTSTRLAPVSPAY : 647
HNB         : RYSSAGTILETVLIESKDMEEQLMAGANSTIPSFNPTLHGGDVKALMLFPNGTVVDLLSFTSTRLAPVSPAY : 647
HNX         : RYSSAGTILETVLIESKDMEEQLMAGANSTIPSFNPTLHGGDVKALMLFPNGTVVDLLSFTSTRLAPVSPAY : 648
JS-2012     : RYSSAGTILETVLIESKDMEEQLMAGANSTIPSFNPTLHGGDAKALMLFPNGTVVDLLSFTSTRLAPVSPAY : 647
TJ          : RYSSAGTILETVLIESKDMEEQLMAGANSTIPSFNPTLHGGDVKALMLFPNGTVVDLLSFTSTRLAPVSPAY : 647
Ea          : RYSSAGTILETVLIESKDMEEQLMAGANSTIPSFNPTLHGGDVKALMLFPNGTVVDLLSFTSTRLAPVSPAY : 647
Fa          : RYSSAGTILETVLIESKDMEEQLMAGANSTIPSFNPTLHGGDVKALMLFPNGTVVDLLSFTSTRLAPVSPAY : 647
SC          : RYSSAGTILETVLIESKDMEEQLMAGANSTIPSFNPTLHGGDVKALMLFPNGTVVDLLSFTSTRLAPVSPAY : 647
Bartha-K61  : RYSSAGTILETVLIESKDMEEQLMAGANSTIPSFNPTLHGGDVKALMLFPNGTVVDLLSFTSTRLAPVSPAY : 647
              RYSSAGTILETVLIESKDMEEQLMAGANSTIPSFNPTLHGGDvKALMLFPNGTVVDLLSFTStRLAPVSPAY

               *        660         *        680
HeN21       : VVASVVGAAITVGILYALFKMLCSFSSEGYSRLINARS* : 685
HuB20       : VVASVVGAAITVGILYALFKMLCSFSSEGYSRLINARS* : 685
hSD-1       : VVASVVGAAITVGILYALFKMLCSFSSEGYSRLINARS- : 685
HeN1        : VVASVVGAAITVGILYALFKMLCSFSSEGYSRLINARS- : 686
HLJ8        : VVASVVGAAITVGILYALFKMLCSFSSEGYSRLINARS- : 685
HN1201      : VVASVVGAAITVGILYALFKMLCSFSSEGYSRLINARS- : 685
HNB         : VVASVVGAAITVGILYALFKMLCSFSSEGYSRLINARS- : 685
HNX         : VVASVVGAAITVGILYALFKMLCSFSSEGYSRLINARS- : 686
JS-2012     : VVASVVGAAITVGILYALFKMLCSFSSEGYSRLINARS- : 685
TJ          : VVASVVGAAITVGILYALFKMLCSFSSEGYSRLINARS- : 685
Ea          : VVASVVGAAITVGILYALFKMLCSFSSEGYSRLINARS- : 685
Fa          : VVASVVGAAITVGILYALFKMLCSFSSEGYSRLINARS- : 685
SC          : VVASVVGAAITVGILYALFKMLCSFSSEGYSRLINARS- : 685
Bartha-K61  : VVASVVGAAITVGILYALFKMLCSFSSEGYSRLINARS* : 685
              VVASVVGAAITVGILYALFKMLCSFSSEGYSRLINARS
```

gI

```
                          *        20         *        40         *        60         *
HeN21    : -MMVARDVTRLPAGLLLAALTLATLTPRVGGVLFRGAGVSVHVAGSAVLVPGDAPNLTIDGTLLFLEGPSPSNYS :  74
HuB20    : MMMVARDVTRLPAGLLLAALTLAALTPRVGGVLFRGAGVSVHVAGSAVLVPGDAPNLTIDGTLLFLEGPSPSNYS :  75
hSD-1    : MMMVARDVTRLPAGLLLAALTLAALTPRVGGVLFRGAGVSVHVAGSAVLVPGDAPNLTIDGTLLFLEGPSPSNYS :  75
HeN1     : -MMVARDVTRLPAGLLLAALTLAALTPRVGGVLFRGAGVSVHVAGSAVLVPGDAPNLTIDGTLLFLEGPSPSNYS :  74
HLJ8     : -MMVARDVTRLPAGLLLAALTLAALTPRVGGVLFRGAGVSVHVAGSAVLVPGDAPNLTIDGTLLFLEGPSPSNYS :  74
HN1201   : MMMVARDVTRLPAGLLLAALTLAALTPRVGGVLFRGAGVSVHVAGSAVLVPGDAPNLTIDGTLLFLEGPSPSNYS :  75
HNB      : MMMVARDVTRLPAGLLLAALTLAALTPRVGGVLFRGAGVSVHVAGSAVLVPGDAPNLTIDGTLLFLEGPSPSNYS :  75
HNX      : MMMVARDVTRLPAGLLLAALTLAALTPRVGGVLFRGAGVSVHVAGSAVLVPGDAPNLTIDGTLLFLEGPSPSNYS :  75
JS-2012  : MMMVARDVTRLPAGLLLAALTLAALTPRVGGVLFRGAGVSVHVAGSAVLVPGDAPNLTIDGTLLFLEGPSPSNYS :  75
TJ       : MMMVARDVTRLPAGLLLAALTLAALTPRVGGVLFRGAGVSVHVAGSAVLVPGDAPNLTIDGTLLFLEGPSPSNYS :  75
Ea       : MMMVARDVTRLPAGLLLAALTLAALTPRVGGVLFRGAGVSVHVAGSAVLVPGDAPNLTIDGTLLFLEGPSPSNYS :  75
Fa       : MMMVARDVTRLPAGLLLAALTLAALTPRVGGVLFRGAGVSVHVAGSAVLVPGDAPNLTIDGTLLFLEGPSPSNYS :  75
SC       : -MMVARDVTRLPAGLLLAALTLAALTPRVGGVLFRGAGVSVHVAGSAVLVPGDAPNLTIDGTLLFLEGPSPSNYS :  74
            MMVARDVTRLPAGLLLAALTLAaLTPRVGGVLFRGAGVSVHVAGSAVLVPGDAPNLTIDGTLLFLEGPSPSNYS

              80         *       100         *       120         *       140         *
HeN21    : GRVELLRLDPKRACYTREYAAEYDLCPRVHHEAFRGCLRKREPLARRASAAVEARRLLFVSRPASGDAGSYVLRV : 149
HuB20    : GRVELLRLDPKRACYTREYAAEYDLCPRVHHEAFRGCLRKREPLARRASAAVEARRLLFVSRPASGDAGSYVLRV : 150
hSD-1    : GRVELLRLDPKRACYTREYAAEYDLCPRVHHEAFRGCLRKREPLARRASAAVEARRLLFVSRPASGDAGSYVLRV : 150
HeN1     : GRVELLRLDPKRACYTREYAAEYDLCPRVHHEAFRGCLRKREPLARRASAAVEARRLLFVSRPASGDAGSYVLRV : 149
HLJ8     : GRVELLRLDPKRACYTREYAAEYDLCPRVHHEAFRGCLRKREPLARRASAAVEARRLLFVSRPASGDAGSYVLRV : 149
HN1201   : GRVELLRLDPKRACYTREYAAEYDLCPRVHHEAFRGCLRKREPLARRASAAVEARRLLFVSRPASGDAGSYVLRV : 150
HNB      : GRVELLRLDPKRACYTREYAAEYDLCPRVHHEAFRGCLRKREPLARRASAAVEARRLLFVSRPASGDAGSYVLRV : 150
HNX      : GRVELLRLDPKRACYTREYAAEYDLCPRVHHEAFRGCLRKREPLARRASAAVEARRLLFVSRPASGDAGSYVLRV : 150
JS-2012  : GRVELLRLDPKRACYTREYAAEYDLCPRVHHEAFRGCLRKREPLARRASAAVEARRLLFVSRPASGDAGSYVLRV : 150
TJ       : GRVELLRLDPKRACYTREYAAEYDLCPRVHHEAFRGCLRKREPLARRASAAVEARRLLFVSRPASGDAGSYVLRV : 150
Ea       : GRVELLRLDPKRACYTREYAAEYDLCPRVHHEAFRGCLRKREPLARRASAAVEARRLLFVSRPASGDAGSYVLRV : 150
Fa       : GRVELLRLDPKRACYTREYAAEYDLCPRVHHEAFRGCLRKREPLARRASAAVEARRLLFVSRPASGDAGSYVLRV : 150
SC       : GRVELLRLDPKRACYTREYAAEYDLCPRVHHEAFRGCLRKREPLARRASAAVEARRLLFVSRPASGDAGSYVLRV : 149
           GRVELLRLDPKRACYTREYAAEYDLCPRVHHEAFRGCLRKREPLARRASAAVEARRLLFVSRPASGDAGSYVLRV
```

```
                    160         *       180         *       200         *       220
HeN21    : RVNGTTDLFVLTALVPPRGRPVPTSPPADECRPVVGSWHDSLRVVDPAEDAVFTTQPPPEPEPPTTPAPPRGTGA : 224
HuB20    : RVNGTTDLFVLTALVPPRGRPVPTSPSADECRPVVGSWHDSLRVVDPAEDAVFTTQPPPEPEPPTTPAPPRGTGA : 225
hSD-1    : RVNGTTDLFVLTALVPPRGRPVPTSPPADECRPVVGSWHDSLRVVDPAEDAVFTTQPPPEPEPPTTPAPPRGTGA : 225
HeN1     : RVNGTTDLFVLTALVPPRGRPVPTSPPADECRPVVGSWHDSLRVVDPAEDAVFTTQPPPEPEPPTTPAPPRGTGA : 224
HLJ8     : RVNGTTDLFVLTALVPPRGRPVPTSPPADECRPVVGSWHDSLRVVDPAEDAVFTTQPPPEPEPPTTPAPPRGTGA : 224
HN1201   : RVNGTTDLFVLTALVPPRGRPVPTSPPADECRPVVGSWHDSLRVVDPAEDAVFTTQPPPEPEPPTTPAPPRGTGA : 225
HNB      : RVNGTTDLFVLTALVPPRGRPVPTSPPADECRPVVGSWHDSLRVVDPAEDAVFTTQPPPEPEPPTTPAPPRGTGA : 225
HNX      : RVNGTTDLFVLTALVPPRGRPVPTSPPADECRPVVGSWHDSLRVVDPAEDAVFTTQPPPEPEPPTTPAPPRGTGA : 225
JS-2012  : RVNGTTDLFVLTALVPPRGRPVPTSPPADECRPVVGSWHDSLRVVDPAEDAVFTTQPPPEPEPPTTPAPPRGTGA : 225
TJ       : RVNGTTDLFVLTALVPPRGRPVPTSPPADECRPVVGSWHDSLRVVDPAEDAVFTTQPPPEPEPPTTPAPPRGTGA : 225
Ea       : RVNGTTDLFVLTALVPPRGRPVPTSPPADECRPVVGSWHDSLRVVDPAEDAVFTTQPPPEPEPPTTPAPPRGTGA : 225
Fa       : RVNGTTDLFVLTALVPPRGRPVPTSPPADECRPVVGSWHDSLRVVDPAEDAVFTTQPPPEPEPPTTPAPPRGTGA : 225
SC       : RVNGTTDLFVLTALVPPRGRPVPTSPPADECRPVVGSWHDSLRVVDPAEDAVFTTQPPPEPEPPTTPAPPRGTGA : 224
           RVNGTTDLFVLTALVPPRGRPVPTSPpADECRPVVGSWHDSLRVVDPAEDAVFTTQPPPEPEPPTTPAPPRGTGA

               *         240         *       260         *       280         *       300
HeN21    : TPEPRSDEEEEGDAETTTPTLTPAPGTLDANGTMVLNASVVSRVLLAAANATAGARSPGKIAMVLGPTIVVLLIF : 299
HuB20    : TPEPRSDEEEEGDAETTTPTMTPAPGTLDANGTMVLNASVVSRVLLAAANATAGARSPGKIAMVLGPTIVVLLIF : 300
hSD-1    : TPEPRSDEEEEGDAETTTPTLTPAPGTLDANGTMVLNASVVSRVLLAAANATAGARSPGKIAMVLGPTIVVLLIF : 300
HeN1     : TPEPRSDEEEEGDAETTTPTLTPAPGTLDANGTMVLNASVVSRVLLAAANATAGARSPGKIAMVLGPTIVVLLIF : 299
HLJ8     : TPEPRSDEEEEGDAETTTPTLTPAPGTLDANGTMVLNASVVSRVLLAAANATAGARSPGKIAMVLGPTIVVLLIF : 299
HN1201   : TPEPRSDEEEEGDAETTTPTLTPAPGTLDANGTMVLNASVVSRVLLAAANATAGARSPGKIAMVLGPTIVVLLIF : 300
HNB      : TPEPRSDEEEEGDAETTTPTLTPAPGTLDANGTMVLDASVVSRVLLAAANATAGARSPGKIAMVLGPTIVVLLIF : 300
HNX      : TPEPRSDEEEEGDAETTTPTLTPAPGTLDANGTMVLNASVVSRVLLAAANATAGARSPGKIAMVLGPTIVVLLIF : 300
JS-2012  : TPEPRSDEEEEGDAETTTPTLTPAPGTLDANGTMVLNASVVSRVLLAAANATAGARSPGKIAMVLGPTIVVLLIF : 300
TJ       : TPEPRSDEEEEGDAETTTPTLTPAPGTLDANGTMVLNASVVSRVLLAAANATAGARGPGKIAMVLGPTIVVLLIF : 300
Ea       : TPEPRSDEEEEGDAETTTPTLTPAPGTLDANGTMVLNASVVSRVLLAAANATAGARSPGKIAMVLGPTIVVLLIF : 300
Fa       : TPEPRSDEEEEGDAETTTPTLTPAPGTLDANGTMVLNASVVSRVLLAAANATAGARSPGKIAMVLGPTIVVLLIF : 300
SC       : TPEPRSDEEEEGDAETTTPTLTPAPGTLDANGTMVLNASVVSRVLLAAANATAGARSPGKIAMVLGPTIVVLLIF : 299
           TPEPRSDEEEEGDAETTTPT6TPAPGTLDANGTMVL1ASVVSRVLLAAANATAGARsPGKIAMVLGPTIVVLLIF
```

```
                          *         320         *         340         *         360
HeN21    : LGGIACVARRCARNRIYRPRPGRGSAVHAAPPRRPPPNPVAGAPVPQPKMTLAELRQKLATIAEEQ* : 365
HuB20    : LGGIACVARRCARNRIYRPRPGRGSAVHAAPPRRPPPNPVAGAPVPQPKMTLAELRQKLATIAEEQ* : 366
hSD-1    : LGGIACVARRCARNRIYRPRPGRGSAVHAAPPRRPPPNPVAGAPVPQPKMTLAELRQKLATIAEEQ- : 366
HeN1     : LGGIACVARRCARNRIYRPRPGRGSAVHAAPPRRPPPNPVAGAPVPQPKMTLAELRQKLATIAEEQ- : 365
HLJ8     : LGGIACVARRCARNRIYRPRPGRGSAVHAAPPRRPPPNPVAGAPVPQPKMTLAELRQKLATIAEEQ- : 365
HN1201   : LGGIACVARRCARNRIYRPRPGRGSAVHAAPPRRPPPNPVAGAPVPQPKMTLAELRQKLATIAEEQ- : 366
HNB      : LGGIACVARRCARNRIYRPRPGRGSAVHAAPPRRPPPNPVAGAPVPQPKMTLAELRQKLATIAEEQ- : 366
HNX      : LGGIACVARRCARNRIYRPRPGRGSAVHAAPPRRPPPNPVAGAPVPQPKMTLAELRQKLATIAEEQ- : 366
JS-2012  : LGGIACVARRCARNRIYRPRPGRGSAVHAAPPRRPPPNPVAGAPVPQPKMTLAELRQKLATIAEEQ- : 366
TJ       : LGGIACVARRCARNRIYRPRPGRGSAVHAAPPRRPPPNPVAGAPVPQPKMTLAELRQKLATIAEEQ- : 366
Ea       : LGGIACVARRCARNRIYRPRPGRGSAVHAAPPRRPPPNPVAGAPVPQPKMTLAELRQKLATIAEEQ- : 366
Fa       : LGGIACVARRCARNRIYRPRPGRGSAVHAAPPRRPPPNPVAGAPVPQPKMTLAELRQKLATIAEEQ- : 366
SC       : LGGIACVARRCARNRIYRPRPGRGSAVHAAPPRRPPPNPVAGAPVPQPKMTLAELRQKLATIAEEQ- : 365
           LGGIACVARRCARNRIYRPRPGRGSAVHAAPPRRPPPNPVAGAPVPQPKMTLAELRQKLATIAEEQ
```

gK

```
                             *        20         *        40         *        60         *
HeN21       : MLLGGRPLHLLVLGVMGAYAGLGAYYATVARLPHPVVYAALPLGEHAGGGAPDWEAFNATAIYVGPNETGAL :  72
HuB20       : MLLGGRPLHLLVLGVMGAYAGLGAYYATVARLPHPVVYAALPLGEHAGGGAPDWEAFNATAIYVGPNETGAL :  72
hSD-1       : MLLGGRPLHLLVLGVMGAYAGLGAYYATVARLPHPVVYAALPLGEHAGGGAPDWEAFNATAIYVGPNETGAL :  72
HeN1        : MLLGGRPLHLLVLGVMGAYAGLGAYYATVARLPHPVVYAALPLGEHAGGGAPDWEAFNATAIYVGPNETGAL :  72
HLJ8        : MLLGGRPLHLLVLGVMGAYAGLGAYYATVARLPHPVVYAALPLGEHAGGGAPDWEAFNATAIYVGPNETGAL :  72
HN1201      : MLLGGRPLHLLVLGVMGAYAGLGAYYATVARLPHPVVYAALPLGEHAGGGAPDWEAFNATAIYVGPNETGAL :  72
HNB         : MLLGGRPLHLLVLGVMGAYAGLGAYYATVARLPHPVVYAALPLGEHAGGGAPDWEAFNATAIYVGPNETGAL :  72
HNX         : MLLGGRPLHLLVLGVMGAYAGLGAYYATVARLPHPVVYAALPLGEHAGGGAPDWEAFNATAIYVGPNETGAL :  72
JS-2012     : MLLGGRPLHLLVLGVMGAYAGLGAYYATVARLPHPVVYAALPLGEHAGGGAPDWEAFNATAIYVGPNETGAL :  72
TJ          : MLLGGRPLHLLVLGVMGAYAGLGAYYATVARLPHPVVYAALPLGEHAGGGAPDWEAFNATAIYVGPNETGAL :  72
Ea          : MLLGGRPLHLLVLGVMGAYAGLGAYYATVARLPHPVVYAALPLGEHAGGGAPDWEAFNATAIYVGPNETGAL :  72
Fa          : MLLGGRPLHLLVLGVMGAYAGLGAYYATVARLPHPVVYAALPLGEHAGGGAPDWEAFNATAIYVGPNETGAL :  72
SC          : MLLGGRPLHLLVLGVMGAYAGLGAYYATVARLPHPVVYAALPLGEHAGGGAPDWEAFNATAIYVGPNETGAL :  72
Bartha-K61  : MLLGGRPLHLLVLGVMGAYAGLGAYYATVARLPHPVVYAALPLGEDAAGGAPDWEAFNATAIYVAPNETDAL :  72
              MLLGGRPLHLLVLGVMGAYAGLGAYYATVARLPHPVVYAALPLGEhAgGGAPDWEAFNATAIYVgPNETgAL

                     80         *       100         *       120         *       140
HeN21       : SPELRDRARVVYARRDCRAYLWDVHFRLAALAWLLYAAFVYARQERRMFGPFRDPAEFLTPEKYTLNYAASV : 144
HuB20       : SPELRDRARVVYARRDCRAYLWDVHFRLAALAWLLYAAFVYARQERRMFGPFRDPAEFLTPEKYTLNYAASV : 144
hSD-1       : SPELRDRARVVYARRDCRAYLWDVHFRLAALAWLLYAAFVYARQERRMFGPFRDPAEFLTPEKYTLNYAASV : 144
HeN1        : SPELRDRARVVYARRDCRAYLWDVHFRLAALAWLLYAAFVYARQERRMFGPFRDPAEFLTPEKYTLNYAASV : 144
HLJ8        : SPELRDRARVVYARRDCRAYLWDVHFRLAALAWLLYAAFVYARQERRMFGPFRDPAEFLTPEKYTLNYAASV : 144
HN1201      : SPELRDRARVVYARRDCRAYLWDVHFRLAALAWLLYAAFVYARQERRMFGPFRDPAEFLTPEKYTLNYAASV : 144
HNB         : SPELRDRARVVYARRDCRAYLWDVHFRLAALAWLLYAAFVYARQERRMFGPFRDPAEFLTPEKYTLNYAASV : 144
HNX         : SPELRDRARVVYARRDCRAYLWDVHFRLAALAWLLYAAFVYARQERRMFGPFRDPAEFLTPEKYTLNYAASV : 144
JS-2012     : SPELRDRARVVYARRDCRAYLWDVHFRLAALAWLLYAAFVYARQERRMFGPFRDPAEFLTPEKYTLNYAASV : 144
TJ          : SPELRDRARVVYARRDCRAYLWDVHFRLAALAWLLYAAFVYARQERRMFGPFRDPAEFLTPEKYTLNYAASV : 144
Ea          : SPELRDRARVVYARRDCRAYLWDVHFRLAALAWLLYAAFVYARQERRMFGPFRDPAEFLTPEKYTLNYAASV : 144
Fa          : SPELRDRARVVYARRDCRAYLWDVHFRLAALAWLLYAAFVYARQERRMFGPFRDPAEFLTPEKYTLNYAASV : 144
SC          : SPELRDRARVVYARRDCRAYLWDVHFRLAALAWLLYAAFVYARQERRMFGPFRDPAEFLTPEKYTLNYAASV : 144
Bartha-K61  : SPALRDRARVVYARRDCRAYLWDVHFRLAAVAWLLYAAFVYARQERRMFGPFRDPAEFLTPEKYTLNYAASV : 144
              SPeLRDRARVVYARRDCRAYLWDVHFRLAA6AWLLYAAFVYARQERRMFGPFRDPAEFLTPEKYTLNYAASV
```

```
                 *         160         *         180         *         200         *
HeN21        : LAATVIGCSYTKFAWYMAELATRRAALSRDLREDPITLAHRHPTLIALILLEIGLRLGARMALFTTLGVTRA : 216
HuB20        : LAATVIGCSYTKFAWYMAELATRRAALSRDLREDPITLAHRHPTLIALILLEIGLRLGARMALFTTLGVTRA : 216
hSD-1        : LAATVIGCSYTKFAWYMAELATRRAALSRDLREDPITLAHRHPTLIALILLEIGLRLGARMALFTTLGVTRA : 216
HeN1         : LAATVIGCSYTKFAWYMAELATRRAALSRDLREDPITLAHRHPTLIALILLEIGLRLGARMALFTTLGVTRA : 216
HLJ8         : LAATVIGCSYTKFAWYMAELATRRAALSRDLREDPITLAHRHPTLIALILLEIGLRLGARMALFTTLGVTRA : 216
HN1201       : LAATVIGCSYTKFAWYMAELATRRAALSRDLREDPITLAHRHPTLIALILLEIGLRLGARMALFTTLGVTRA : 216
HNB          : LAATVIGCSYTKFAWYMAELATRRAALSRDLREDPITLAHRHPTLIALILLEIGLRLGARMALFTTLGVTRA : 216
HNX          : LAATVIGCSYTKFAWYMAELATRRAALSRDLREDPITLAHRHPTLIALILLEIGLRLGARMALFTTLGVTRA : 216
JS-2012      : LAATVIGCSYTKFAWYMAEPATRRAAPSRDLREDPITLAHRHPTLIALILLEIGLRLGARMALFTTLGVTRA : 216
TJ           : LAATVIGCSYTKFAWYMAELATRRAALSRDLREDPITLAHRHPTLIALILLEIGLRLGARMALFTTLGVTRA : 216
Ea           : LAATVIGCSYTKFAWYMAELATRRAALSRDLREDPITLAHRHPTLIALILLEIGLRLGARMALFTTLGVTRA : 216
Fa           : LAATVIGCSYTKFAWYMAELATRRAALSRDLREDPITLAHRHPTLIALILLEIGLRLGARMALFTTLGVTRA : 216
SC           : LAATVIGCSYTKFAWYMAELATRRAALSRDLREDPITLAHRHPTLIALILLEIGLRLGARMALFTTLGVTRA : 216
Bartha-K61   : LAATVIGCSYTKFAWYMAELATRRAALSRDLREDPITLAHRHPTLIALILLELGLRLGARMALFTTLGVTRA : 216
               LAATVIGCSYTKFAWYMAElATRRAAlSRDLREDPITLAHRHPTLIALILLE6GLRLGARMALFTTLGVTRA

                220         *         240         *         260         *         280
HeN21        : PCALVFPLYARALVWLFVLAVGALELLAATLPHIARVSGATAPPARADGGRAALGVCGACCSTVLAGIFAKA : 288
HuB20        : PCALVFPLYARALVWLFVLAVGALELLAATLPHIARVSGATAPPARADGGRAALGVCGACCSTVLAGIFAKA : 288
hSD-1        : PCALVFPLYARALVWLFVLAVGALELLAATLPHIARVSGATAPPARADGGRAALGVCGACCSTVLAGIFAKA : 288
HeN1         : PCALVFPLYARALVWLFVLAVGALELLAATLPHIARVSGATAPPARADGGRAALGVCGACCSTVLAGIFAKA : 288
HLJ8         : PCALVFPLYARALVWLFVLAVGALELLAATLPHIARVSGATAPPARADGGRAALGVCGACCSTVLAGIFAKA : 288
HN1201       : PCALVFPLYARALVWLFVLAVGALELLAATLPHIARVSGATAPPARADGGRAALGVCGACCSTVLAGIFAKA : 288
HNB          : PCALVFPLYARALVWLFVLAVGALELLAATLPHIARVSGATAPPARADGGRAALGVCGACCSTVLAGIFAKA : 288
HNX          : PCALVFPLYARALVWLFVLAVGALELLAATLPHIARVSGATAPPARADGGRAALGVCGACCSTVLAGIFAKA : 288
JS-2012      : PCALVFPLYARALVWLFVLAVGALELLAATLPHIARVSGATAPPARADGGRAALGVCGACCSTVLAGIFAKA : 288
TJ           : PCALVFPLYARALVWLFVLAVGALELLAATLPHIARVSGATAPPARADGGRAALGVCGACCSTVLAGIFAKA : 288
Ea           : PCALVFPLYARALVWLFVLAVGALELLAATLPHIARVSGATAPPARADGGRAALGVCGACCSTVLAGIFAKA : 288
Fa           : PCALVFPLYARALVWLFVLAVGALELLAATLPHIARVSGATAPPARADGGRAALGVCGACCSTVLAGIFAKA : 288
SC           : PCALVFPLYARALVWLFVLAVGALELLAATLPHIARVSGATAPPARADGGRAALGVCGACCSTVLAGIFAKA : 288
Bartha-K61   : PCALVFPLYARALVWIFVLAVGALELLAATLPHIARVSGATATPARSDGGRAALGVCGACCSTVLAGIFAKA : 288
               PCALVFPLYARALVW6FVLAVGALELLAATLPHIARVSGATApPARaDGGRAALGVCGACCSTVLAGIFAKA
```

```
                  *         300          *
HeN21       : LYLLLLVGGVLLFLHYERHITIFG* : 312
HuB20       : LYLLLLVGGVLLFLHYERHITIFG* : 312
hSD-1       : LYLLLLVGGVLLFLHYERHITIFG- : 312
HeN1        : LYLLLLVGGVLLFLHYERHITIFG- : 312
HLJ8        : LYLLLLVGGVLLFLHYERHITIFG- : 312
HN1201      : LYLLLLVGGVLLFLHYERHITIFG- : 312
HNB         : LYLLLLVGGVLLFLHYERHITIFG- : 312
HNX         : LYLLLLVGGVLLFLHYERHITIFG- : 312
JS-2012     : LYLLLLVGGVLLFLHYERHITIFG- : 312
TJ          : LYLLLLVGGVLLFLHYERHITIFG- : 312
Ea          : LYLLLLVGGVLLFLHYERHITIFG- : 312
Fa          : LYLLLLVGGVLLFLHYERHITIFG- : 312
SC          : LYLLLLVGGVLLFLHYERHITIFG- : 312
Bartha-K61  : LYLCLLVGGVLLFLHYERHITIFG* : 312
              LYLlLLVGGVLLFLHYERHITIFG
```

gL

```
                              *        20         *        40         *        60         *
HeN21        : MSPLVAVLVFFSAALGIPGPGVAGNPRGLDAIFEAPVT-----PAPPTRHPRREELEWDDEDHPLLDLEPPV :  67
HuB20        : MSPLVAVLVFFSAALGIPGPGVAGNPRGLDAIFEAPVT-----PAPPTRHPRREELEWDDEDHPLLDLEPPV :  67
hSD-1        : MSPLVAVLVFFSAALGIPGPGVAGNPRGLDAIFEAPVT-----PAPPTRHPRREELEWDDEDHPLLDLEPPV :  67
HeN1         : MSPLVAVLVFFSAALGIPGPGVAGNPRGLDAIFEPPVT-----PAPPTRAPRREELEWDDEDHPLLGLEPPV :  67
HLJ8         : MSPLVAVLVFFSAALGIPGPGVAGNPRGLDAIFEAPVT-----PAPPTRHPRREELEWDDEDHPLLDLEPPV :  67
HN1201       : MSPLVAVLVFFSAALGIPGPGVAGNPRGLDAIFEAPVT-----PAPPTRHPRREELEWDDEDHPLLDLEPPV :  67
HNB          : MSPLVAVLVFFSAALGIPGPGVAGNPRGLDAIFEAPVT-----PAPPTRHPRREELEWDDEDHPLLDLEPPV :  67
HNX          : MSPLVAVLVFFSAALGIPGPGVAGNPRGLDAIFEAPVT-----PAPPTRHPRREELEWDDEDHPLLDLEPPV :  67
JS-2012      : MSPLVAVLVFFSAALGIPGPGVAGNPRGLDAIFEAPVT-----PAPPTRHPRREELEWDDEDHPLLDLEPPV :  67
TJ           : MSPLVAVLVFFSAALGIPGPGVAGNPRGLDAIFEAPVT-----PAPPTRHPRREELEWDDEDHPLLDLEPPV :  67
Ea           : MSPLVAVLVFFSAALGIPGPGVAGNPRGLDAIFEAPVT-----PAPPTRHPRREELEWDDEDHPLLDLEPPV :  67
Fa           : MSPLVAVLVFFSAALGIPGPGVAGNPRGLDAIFEAPVTPAPVTPAPPTRHPRREELEWDDEDHPLLDLEPPV :  72
SC           : MSPLVAVLVFFSAALGIPGPGVAGNPRGLDAIFEAPVT-----PAPPTRHPRREELEWDDEDHPLLDLEPPV :  67
Bartha-K61   : MSPLVAVLVFFSAALGVPGTGVAGNPHGLDAIFEPPVT-----PAPPTRAPRREELEWDDEDHPLLGLEPPV :  67
               MSPLVAVLVFFSAALG6PGpGVAGNPrGLDAIFEaPVT     PAPPTRhPRREELEWDDEDHPLLdLEPPV

                           80         *       100         *       120         *       140
HeN21        : GSRCHPYIAYSLPPDMNAVTSVVVKPYCSPPEVILWASGTAYLVNPFVAIQALAVGEPLNEAALKELGEVAV : 139
HuB20        : GSRCHPYIAYSLPPDMNAVTSVVVKPYCSPPEVILWASGTAYLVNPFVAIQALAVGEPLNEAALKELGEVAV : 139
hSD-1        : GSRCHPYIAYSLPPDMNAVTSVVVKPYCSPPEVILWASGTAYLVNPFVAIQALAVGEPLNEAALKELGEVAV : 139
HeN1         : GSRCHPYIAYSLPPDMTAVTSVVVKPYCSPPEVILWASGTAYLVNPFVAIQALAIGEPLNEAALKELGEVAV : 139
HLJ8         : GSRCHPYIAYSLPPDMNAVTSVVVKPYCSPPEVILWASGTAYLVNPFVAIQALAVGEPLNEAALKELGEVAV : 139
HN1201       : GSRCHPYIAYSLPPDMNAVTSVVVKPYCSPPEVILWASGTAYLVNPFVAIQALAVGEPLNEAALKELGEVAV : 139
HNB          : GSRCHPYIAYSLPPDMNAVTSVVVKPYCSPPEVILWASGTAYLVNPFVAIQALAVGEPLNEAALKELGEVAV : 139
HNX          : GSRCHPYIAYSLPPDMNAVTSVVVKPYCSPPEVILWASGTAYLVNPFVAIQALAVGEPLNEAALKELGEVAV : 139
JS-2012      : GSRCHPYIAYSLPPDMNAVTSVVVKPYCSPPEVILWASGTAYLVNPFVAIQALAVGEPLNEAALKELGEVAV : 139
TJ           : GSRCHPYIAYSLPPDMNAVTSVVVKPYCSPPEVILWASGTAYLVNPFVAIQALAVGEPLNEAALKELGEVAV : 139
Ea           : GSRCHPYIAYSLPPDMNAVTSVVVKPYCSPPEVILWASGTAYLVNPFVAIQALAVGEPLNEAALKELGEVAV : 139
Fa           : GSRCHPYIAYSLPPDMNAVTSVVVKPYCSPPEVILWASGTAYLVNPFVAIQALAVGEPLNEAALKELGEVAV : 144
SC           : GSRCHPYIAYSLPPDMNAVTSVVVKPYCSPPEVILWASGTAYLVNPFVAIQALAVGEPLNEAALKELGEVAV : 139
Bartha-K61   : GSRCHPYIAYSLPPDMTAVTSVVVKPYCSPPEVILWASGTAYLVNPFVAIQALAIGEPLNEAALKELGEVAV : 139
               GSRCHPYIAYSLPPDMnAVTSVVVKPYCSPPEVILWASGTAYLVNPFVAIQALA6GEPLNEAALKELGEVAV
```

```
                         *        160
HeN21        : HKDSLPPLRYNGGPPAE* : 156
HuB20        : HKDSLPPLRYNGGPPAE* : 156
hSD-1        : HKDSLPPLRYNGGPPAE- : 156
HeN1         : HKDSLPPLRYNGGPPAE- : 156
HLJ8         : HKDSLPPLRYNGGPPAE- : 156
HN1201       : HKDSLPPLRYNGGPPAE- : 156
HNB          : HKDSLPPLRYNGGPPAE- : 156
HNX          : HKDSLPPLRYNGGPPAE- : 156
JS-2012      : HKDSLPPLRYNGGPPAE- : 156
TJ           : HKDSLPPLRYNGGPPAE- : 156
Ea           : HKDSLPPLRYNGGPPAE- : 156
Fa           : HKDSLPPLRYNGGPPAE- : 161
SC           : HKDSLPPLRYNGGPPAE- : 156
Bartha-K61 : HKDSLPPLRYNGGPPAE* : 156
               HKDSLPPLRYNGGPPAE
```

gM

```
                           *        20         *        40         *        60         *
HeN21       : MSGPRNAEAVSWRSWLIEVCGFALAALTLVLTLIFASLPEMGFPCFYATVADYDTLNDTSGGVWTRQPLVAP :  72
HuB20       : MSGPRNAEAVSWRSWLIEVCGFALAALTLVLTLIFASLPEMGFPCFYATVADYDTLNDTSGGVWTRQPLVAP :  72
hSD-1       : MSGPRNAEAVSWRSWLIEVCGFALAALTLVLTLIFASLPEMGFPCFYATVADYDTLNDTSGGVWTRQPLVAP :  72
HeN1        : MSGPRNAEAVSWRSWLIEVCGFALAALTLVLTLIFASLPEMGFPCFYATVADYDTLNDTSGGVWTRQPLVAP :  72
HLJ8        : MSGPRNAEAVSWRSWLIEVCGFALAALTLVLTLIFASLPEMGFPCFYATVADYDTLNDTSGGVWTRQPLVAP :  72
HN1201      : MSGPRNAEAVSWRSWLIEVCGFALAALTLVLTLIFASLPEMGFPCFYATVADYDTLNDTSGGVWTRQPLVAP :  72
HNB         : MSGPRNAEAVSWRSWLIEVCGFALAALTLVLTLIFASLPEMGFPCFYATVADYDTLNDTSGGVWTRQPLVAP :  72
HNX         : MSGPRNAEAVSWRSWLIEVCGFALAALTLVLTLIFASLPEMGFPCFYATVADYDTLNDTSGGVWTRQPLVAP :  72
JS-2012     : MSGPRNAEAVSWRSWLIEVCGFALAALTLVLTLIFASLPEMGFPCFYATVADYDTLNDTSGGVWTRQPLVAP :  72
TJ          : MSGPRNAEAVSWRSWLIEVCGFALAALTLVLTLIFASLPEMGFPCFYATVADYDTLNDTSGGVWTRQPLVAP :  72
Ea          : MSGPRNAEAVSWRSWLIEVCGFALAALTLVLTLIFASLPEMGFPCFYATVADYDTLNDTSGGVWTRQPLVAP :  72
Fa          : MSGPRNAEAVSWRSWLIEVCGFALAALTLVLTLIFASLPEMGFPCFYATVADYDTLNDTSGGVWTRQPLVAP :  72
SC          : MSGPRNAEAVSWRSWLIEVCGFALAALTLVLTLIFASLPEMGFPCFYATVADYDTLNDTSGGVWTRQPLVAP :  72
Bartha-K61  : MCGPRNAEAVSWRSWLIEVCGFALAALTLVLTLIFASLPEMGFPCFYATVADYDTLNDAPGGVWTRQPLVAP :  72
              MsGPRNAEAVSWRSWLIEVCGFALAALTLVLTLIFASLPEMGFPCFYATVADYDTLNDtsGGVWTRQPLVAP

                  80         *       100         *       120         *       140
HeN21       : ALFLETPTVTSFFGFTATVLLAHALYAVAGAVVLRREAGRLAFQPSVVLYAASTVAAPGTLMLGALCAWTLQ : 144
HuB20       : ALFLETPTVTSFFGFTATVLLAHALYAVAGAVVLRREAGRLAFQPSVVLYAASTVAAPGTLMLGALCAWTLQ : 144
hSD-1       : ALFLETPTVTSFFGFTATVLLAHALYAVAGAVVLRREAGRLAFQPSVVLYAASTVAAPGTLMLGALCAWTLQ : 144
HeN1        : ALFLETPTVTSFFGFTATVLLAHALYAVAGAVVLRREAGRLAFQPSVVLYAASTVAAPGTLMLGALCAWTLQ : 144
HLJ8        : ALFLETPTVTSFFGFTATVLLAHALYAVAGAVVLRREAGRLAFQPSVVLYAASTVAAPGTLMLGALCAWTLQ : 144
HN1201      : ALFLETPTVTSFFGFTATVLLAHALYAVAGAVVLRREAGRLAFQPSVVLYAASTVAAPGTLMLGALCAWTLQ : 144
HNB         : ALFLETPTVTSFFGFTATVLLAHALYAVAGAVVLRREAGRLAFQPSVVLYAASTVAAPGTLMLGALCAWTLQ : 144
HNX         : ALFLETPTVTSFFGFTATVLLAHALYAVAGAVVLRREAGRLAFQPSVVLYAASTVAAPGTLMLGALCAWTLQ : 144
JS-2012     : ALFLETPTVTSFFGFTATVLLAHALYAVAGAVVLRREAGRLAFQPSVVLYAASTVAAPGTLMLGALCAWTLQ : 144
TJ          : ALFLETPTVTSFFGFTATVLLAHALYAVAGAVVLRREAGRLAFQPSVVLYAASTVAAPGTLMLGALCAWTLQ : 144
Ea          : ALFLETPTVTSFFGFTATVLLAHALYAVAGAVVLRREAGRLAFQPSVVLYAASTVAAPGTLMLGALCAWTLQ : 144
Fa          : ALFLETPTVTSFFGFTATVLLAHALYAVAGAVVLRREAGRLAFQPSVVLYAASTVAAPGTLMLGALCAWTLQ : 144
SC          : ALFLETPTVTSFFGFTATVLLAHALYAVAGAVVLRREAGRLAFQPSVVLYAASTVAAPGTLMLGALCAWTLQ : 144
Bartha-K61  : ALFLETPTVTSFFGFTATVLLAHALYAVAGAVVLRREAGRLAFQPSVVLYAASTVAAPGTLMLGALCAWTLQ : 144
              ALFLETPTVTSFFGFTATVLLAHALYAVAGAVVLRREAGRLAFQPSVVLYAASTVAAPGTLMLGALCAWTLQ
```

```
                     *       160         *       180         *       200         *
HeN21      : AVVLLMAHKQAGLAAAAYITHFVFLALFGACHACKGAGDVRAALAASPPLRRVAVHARAVVTNVVLGAVGLG : 216
HuB20      : AVVLLMAHKQAGLAAAAYITHFVFLALFGACHACKGAGDVRAALAASPPLRRVAVHARAVVTNVVLGAVGLG : 216
hSD-1      : AVVLLMAHKQAGLAAAAYITHFVFLALFGACHACKGAGDVRAALAASPPLRRVAVHARAVVTNVVLGAVGLG : 216
HeN1       : AVVLLMAHKQAGLAAAAYITHFVFLALFGACHACKGAGDVRAALAASPPLRRVAVHARAVVTNVVLGAVGLG : 216
HLJ8       : AVVLLMAHKQAGLAAAAYITHFVFLALFGACHACKGAGDVRAALAASPPLRRVAVHARAVVTNVVLGAVGLG : 216
HN1201     : AVVLLMAHKQAGLAAAAYITHFVFLALFGACHACKGAGDVRAALAASPPLRRVAVHARAVVTNVVLGAVGLG : 216
HNB        : AVVLLMAHKQAGLAAAAYITHFVFLALFGACHACKGAGDVRAALAASPPLRRVAVHARAVVTNVVLGAVGLG : 216
HNX        : AVVLLMAHKQAGLAAAAYITHFVFLALFGACHACKGAGDVRAALAASPPLRRVAVHARAVVTNVVLGAVGLG : 216
JS-2012    : AVVLLMAHKQAGLAAAAYITHFVFLALFGACHACKGAGDVRAALAASPPLRRVAVHARAVVTNVVLGAVGLG : 216
TJ         : AVVLLMAHKQAGLAAAAYITHFVFLALFGACHACKGAGDVRAALAASPPLRRVAVHARAVVTNVVLGAVGLG : 216
Ea         : AVVLLMAHKQAGLAAAAYITHFVFLALFGACHACKGAGDVRAALAASPPLRRVAVHARAVVTNVVLGAVGLG : 216
Fa         : AVVLLMAHKQAGLAAAAYITHFVFLALFGACHACKGAGDVRAALAASPPLRRVAVHARAVVTNVVLGAVGLG : 216
SC         : AVVLLMAHKQAGLAAAAYITHFVFLALFGACHACKGTGDVRAALAASPPLRRVAVHARAVVTNVVLGAVGLG : 216
Bartha-K61 : AVVLLMAHKQAGLAAAAYITHFVFLALFGACHACKGTGDVRAALAASPPLRRVAVHARAVVTNVVLGAVGLG : 216
             AVVLLMAHKQAGLAAAAYITHFVFLALFGACHACKGaGDVRAALAASPPLRRVAVHARAVVTNVVLGAVGLG

              220         *       240         *       260         *       280
HeN21      : AAVVGLMLGVLLANSFHISLWKTAEVALAVFTVLALALMAFVEVVVSGYVQVLPTPAFCVLVASAALGVSAH : 288
HuB20      : AAVVGLMLGVLLANSFHISLWKTAEVALAVFTVLALALMAFVEVVVSGYVQVLPTPAFCVLVASAALGVSAH : 288
hSD-1      : AAVVGLMLGVLLANSFHISLWKTAEVALAVFTVLALALMAFVEVVVSGYVQVLPTPAFCVLVASAALGVSAH : 288
HeN1       : AAVVGLMLGVLLANSFHISLWKTAEVALAVFTVLALALMAFVEVVVSGYVQVLPTPAFCVLVASAALGVSAH : 288
HLJ8       : AAVVGLMLGVLLANSFHISLWKTAEVALAVFTVLALALMAFVEVVVSGYVQVLPTPAFCVLVASAALGVSAH : 288
HN1201     : AAVVGLMLGVLLANSFHISLWKTAEVALAVFTVLALALMAFVEVVVSGYVQVLPTPAFCVLVASAALGVSAH : 288
HNB        : AAVVGLMLGVLLANSFHISLWKTAEVALAVFTVLALALMAFVEVVVSGYVQVLPTPAFCVLVASAALGVSAH : 288
HNX        : AAVVGLMLGVLLANSFHISLWKTAEVALAVFTVLALALMAFVEVVVSGYVQVLPTPAFCVLVASAALGVSAH : 288
JS-2012    : AAVVGLMLGVLLANSFHISLWKTAEVALAVFTVLALALMVFVEVVVSGYVQVLPTPAFCVLVASAALGVSAH : 288
TJ         : AAVVGLMLGVLLANSFHISLWKTAEVALAVFTVLALALMAFVEVVVSGYVQVLPTPAFCVLVASAALGVSAH : 288
Ea         : AAVVGLMLGVLLANSFHISLWKTAEVALAVFTVLALALMAFVEVVVSGYVQVLPAPAFCVLVASAALGVSAH : 288
Fa         : AAVVGLMLGVLLANSFHISLWKTAEVALAVFTVLALALMAFVEVVVSGYVQVLPAPAFCVLVASAALGVSAH : 288
SC         : AAVVGLMLGVLLANSFHISLWKTAEAALAVFTLLALALMVFVEVVVSGYVQVLPTPAFCVLVASAAFGVSAH : 288
Bartha-K61 : AAVVGLMLGVLLANSFHISLWKTAEAALAVFTLLALALMVFVEVVVSGYVQVLPTPAFCVLVASAAFGVSAH : 288
             AAVVGLMLGVLLANSFHISLWKTAEvALAVFT6LALALMaFVEVVVSGYVQVLPtPAFCVLVASAAlGVSAH
```

```
                  *       300        *       320        *       340        *       360
HeN21       : RYFAKFSEALGETHGVVIGTRAVLAVLSLIALAMIVVRLVRACIAHRARGSRFYANVDKARTTARRYLQKRL : 360
HuB20       : RYFAKFSEALGETHGVVIGTRAVLAVLSLIALAMIVVRLVRACIAHRARGSRFYANVDKARTTARRYLQKRL : 360
hSD-1       : RYFAKFSEALGETHGVVIGTRAVLAVLSLIALAMIVVRLVRACIAHRARGSRFYANVDKARTTARRYLQKRL : 360
HeN1        : RYFAKFSEALGETHGVVIGTRAVLAVLSLIALAMIVVRLVRACIAHRARGSRFYANVDKARTTARRYLQKRL : 360
HLJ8        : RYFAKFSEALGETHGVVIGTRAVLAVLSLIALAMIVVRLVRACIAHRARGSRFYANVDKARTTARRYLQKRL : 360
HN1201      : RYFAKFSEALGETHGVVIGTRAVLAVLSLIALAMIVVRLVRACIAHRARGSRFYANVDKARTTARRYLQKRL : 360
HNB         : RYFAKFSEALGETHGVVIGTRAVLAVLSLIALAMIVVRLVRACIAHRARGSRFYANVDKARTTARRYLQKRL : 360
HNX         : RYFAKFSEALGETHGVVIGTRAVLAVLSLIALAMIVVRLVRACIAHRARGSRFYANVDKARTTARRYLQKRL : 360
JS-2012     : RYFAKFSEALGETHGVVIGTRAVLAVLSLIALAMIVVRLVRACIAHRARGSRFYANVDKARTTARRYLQKRL : 360
TJ          : RYFAKFSEALGETHGVVIGTRAVLAVLSLIALAMIVVRLVRACIAHRARGSRFYANVDKARTTARRYLQKRL : 360
Ea          : RYFAKFSEALGETHGVVIGTRAVLAVLSLIALAMIVVRLVRACIAHRARGSRFYANVDKARTTARRYLQKRL : 360
Fa          : RYFAKFSEALGETHGVVIGTRAVLAVLSLIALAMIVVRLVRACIAHRARGSRFYANVDKARTTARRYLQKRL : 360
SC          : RYFAKFSEALGETHGVVIGTRAVLAVLSLIALAMIVVRLVRACIAHRARGSRFYANVDKARTTARRYLQKRL : 360
Bartha-K61  : RYFAKFSEALGETHGVVIGTRAVLAVLSLIALAMIVVRLVRACIAHRARGSRFYANVDKARTTARRYLQKRL : 360
              RYFAKFSEALGETHGVVIGTRAVLAVLSLIALAMIVVRLVRACIAHRARGSRFYANVDKARTTARRYLQKRL

                      *       380         *
HeN21       : HGRGNEEYLLAPGMTEDEFDDGDEVVYENLGFE* : 393
HuB20       : HGRGNEEYLLAPGMTEDEFDDGDEVVYENLGFE* : 393
hSD-1       : HGRGNEEYLLAPGMTEDEFDDGDEVVYENLGFE- : 393
HeN1        : HGRGNEEYLLAPGMTEDEFDDGDEVVYENLGFE- : 393
HLJ8        : HGRGNEEYLLAPGMTEDEFDDGDEVVYENLGFE- : 393
HN1201      : HGRGNEEYLLAPGMTEDEFDDGDEVVYENLGFE- : 393
HNB         : HGRGNEEYLLAPGMTEDEFDDGDEVVYENLGFE- : 393
HNX         : HGRGNEEYLLAPGMTEDEFDDGDEVVYENLGFE- : 393
JS-2012     : HGRGNEEYLLAPGMTEDEFDDGDEVVYENLGFE- : 393
TJ          : HGRGNEEYLLAPGMTEDEFDDGDEVVYENLGFE- : 393
Ea          : HGRGNEEYLLAPGMTEDEFDDGDEVVYENLGFE- : 393
Fa          : HGRGNEEYLLAPGMTEDEFDDGDEVVYENLGFE- : 393
SC          : HGRGNEEYLLAPGSGDDEFDDGDEVVYENLGFE- : 393
Bartha-K61  : HGRGNDEYLLAPGSGDDEFDDGDEVVYENLGFE* : 393
              HGRGNeEYLLAPGmteDEFDDGDEVVYENLGFE
```

gN

```
                            *        20         *        40         *        60         *
HeN21      : MVSSSARLSLTLVAALCALVTPALSSIVFSEGSLPLLREESRTSFWSATCAARGVPVDQPTAAAVTFYICLL :  72
HuB20      : MVSSSARLSLTLVAALCALVTPALSSIVFSEGSLPLLREESRSSFWSAACAARGVPVDQPTAAAVTFYICLL :  72
hSD-1      : MVSSSARLSLTLVAALCALVTPALSSIVFSEGSLPLLREESRTSFWSATCAARGVPVDQPTAAAVTFYICLL :  72
HeN1       : MVSSSARLSLTLVAALCALVTPALSSIVFSEGSLPLLREESRTSFWSATCAARGVPVDQPTAAAVTFYICLL :  72
HLJ8       : MVSSSARLSLTLVAALCALVTPALSSIVFSEGSLPLLREESRTSFWSATCAARGVPVDQPTAAAVTFYICLL :  72
HN1201     : MVSSSARLSLTLVAALCALVTPALSSIVFSEGSLPLLREESRTSFWSATCAARGVPVDQPTAAAVTFYICLL :  72
HNB        : MVSSSARLSLTLVAALCALVTPALSSIVFSEGSLPLLREESRTSFWSATCAARGVPVDQPTAAAVTFYICLL :  72
HNX        : MVSSSARLSLTLVAALCALVTPALSSIVFSEGSLPLLREESRTSFWSATCAARGVPVDQPTAAAVTFYICLL :  72
JS-2012    : MVSSSARLSLTLVAALCALVTPALSSIVFSEGSLPLLREESRTSFWSATCAARGVPVDQPTAAAVTFYICLL :  72
TJ         : MVSSSARLSLTLVAALCALVTPALSSIVFSEGSLPLLREESRTSFWSATCAARGVPVDQPTAAAVTFYICLL :  72
Ea         : MVSSSARLSLTLVAALCALVTPALSSIVFSEGSLPLLREESRTSFWSAACAARGVPVDQPTAAAVTFYICLL :  72
Fa         : MVSSSARLSLTLVAALCALVTPALSSIVFSEGSLPLLREESRTSFWSAACAARGVPVDQPTAAAVTFYICLL :  72
SC         : MVSSSARLSLTLVAALCALVTPALSSIVFSEGSLPLLREESRTSFWSAACAARGVPVDQPTAAAVTFYICLL :  72
Bartha-K61 : MVSS-AGPSLTLVAALCALVAPALSSIVSTEGPLPLLREESRINFWNAACAARGVPVDQPTAAAVTFYICLL :  71
             MVSSsArlSLTLVAALCALVtPALSSIVf3EGsLPLLREESR sFWsA CAARGVPVDQPTAAAVTFYICLL

                    80         *        100
HeN21      : AVLVVALGYATRTCARMLHTSPAGRRV* :  99
HuB20      : AVLVVALGYATRTCARMLHTSPAGRRV* :  99
hSD-1      : AVLVVALGYATRTCARMLHTSPAGRRV- :  99
HeN1       : AVLVVALGYATRTCARMLHTSPAGRRV- :  99
HLJ8       : AVLVVALGYATRTCARMLHTSPAGRRV- :  99
HN1201     : AVLVVALGYATRTCARMLHTSPAGRRV- :  99
HNB        : AVLVVALGYATRTCARMLHTSPAGRRV- :  99
HNX        : AVLVVALGYATRTCARMLHTSPAGRRV- :  99
JS-2012    : AVLVVALGYATRTCTRMLHTSPAGRRV- :  99
TJ         : AVLVVALGYATRTCARMLHTSPAGRRV- :  99
Ea         : AVLVVALGYATRTCARMLHTSPAGRRV- :  99
Fa         : AVLVVALGYATRTCARMLHTSPAGRRV- :  99
SC         : AVLVVALGYATRTCARMLHTSPAGRRV- :  99
Bartha-K61 : AVLVVALGYATRTCTRMLHASPAGRRV* :  98
             AVLVVALGYATRTCaRMLHtSPAGRRV
```
