# Supplementary material for: Characterization of Pseudorabies Virus Associated with Severe Respiratory and Neuronal Signs in Old Pigs
Source: Transbound Emerg Dis. 2023 Feb 28;2023:8855739. doi: 10.1155/2023/8855739 (PMC12017139; doi:10.1155/2023/8855739)
Supplement: Supplementary Materials — Figure S1: Cytotoxic effects caused by different PRV strains on different types of cells. Figure S2: Damages of main organs of fattening pigs caused by different PRV strains. Figure S3: Amino acid sequences alignments of main PRV glycoproteins (gB, gC, gD, gE, gG, gH, gL, gM, gN, and gK) between different PRV strains. Table S1: Reference PRV genome sequences used in this study. Table S2: Stable titers of PRV strains HeN21, HuB20, HBJZ-44-2021, and JSZL-2018 on PK-15 cells. Table S3: Comparisons of viral loads in different organs of pigs between different PRV-challenging groups. Table S4: Pathological injury scores of main organs of mice caused by different PRV strains. Table S5: Pathological injury scores of main organs of fattening pigs caused by different PRV strains. [file 8855739.f1.zip › Table S1.docx]

**Table S1.** Reference PRV genome sequences used in this study.

| **NO.** | **PRV strains** | **GenBank accession numbers** | **Year of isolation** | **Country of isolation** |
| --- | --- | --- | --- | --- |
| 1 | HN1201 | KP722022.1 | 2012 | China |
| 2 | HNB | KM189914.3 | 2012 | China |
| 3 | HNX | KM189912.1 | 2012 | China |
| 4 | HeN1 | KP098534.1 | 2012 | China |
| 5 | HLJ8 | KT824771.1 | 2014 | China |
| 6 | JS-2012 | KP257591.1 | 2012 | China |
| 7 | ZJ01 | KM061380.1 | 2012 | China |
| 8 | TJ | KJ789182.1 | 2012 | China |
| 9 | Fa | KM189913.1 | 1990 | China |
| 10 | Ea | KX423960.1 | 1990 | China |
| 11 | SC | KT809429.1 | 1990 | China |
| 12 | Kolchis | KT983811.1 | 2010 | Greece |
| 13 | Hercules | KT983810.1 | 2010 | Greece |
| 14 | PRV-MdBio | LT934125.1 | 2015 | Serbia |
| 15 | ADV32751 | KU198433.1 | 2014 | Italy |
| 16 | MY-1 | AP018925 | 2019 | Japan |
| 17 | Qihe547 | KU056477.1 | 2015 | China |
| 18 | DUL34Pass | JQ809330.1 | 2012 | Germany |
| 19 | RC1 | LC342744.1 | 2017 | Japan |
| 20 | NIA3 | KU900059.1 | 1970s | Northern Ireland |
| 21 | Kaplan | KJ717942.1 | 1970s | United States |
| 22 | Becker | JF797219.1 | 1960s | United States |
| 23 | Bartha-K61 | JF797217.1 | 1950s | Hungry |
| 24 | AnH1/CHN2015 | MK618718 | 2015 | China |
| 25 | HuN1/CHN2015 | MK682672 | 2015 | China |
| 26 | HeN1/CHN2016 | MK642577 | 2016 | China |
| 27 | GD1/CHN2016 | MK642566 | 2016 | China |
| 28 | HuB1/CHN2017 | MK682670 | 2017 | China |
