# Supplementary material for: Characterization of Pseudorabies Virus Associated with Severe Respiratory and Neuronal Signs in Old Pigs
Source: Transbound Emerg Dis. 2023 Feb 28;2023:8855739. doi: 10.1155/2023/8855739 (PMC12017139; doi:10.1155/2023/8855739)
Supplement: Supplementary Materials — Figure S1: Cytotoxic effects caused by different PRV strains on different types of cells. Figure S2: Damages of main organs of fattening pigs caused by different PRV strains. Figure S3: Amino acid sequences alignments of main PRV glycoproteins (gB, gC, gD, gE, gG, gH, gL, gM, gN, and gK) between different PRV strains. Table S1: Reference PRV genome sequences used in this study. Table S2: Stable titers of PRV strains HeN21, HuB20, HBJZ-44-2021, and JSZL-2018 on PK-15 cells. Table S3: Comparisons of viral loads in different organs of pigs between different PRV-challenging groups. Table S4: Pathological injury scores of main organs of mice caused by different PRV strains. Table S5: Pathological injury scores of main organs of fattening pigs caused by different PRV strains. [file 8855739.f1.zip › Table S2.docx]

**Table S2.** Stable titers of PRV strains HeN21, HuB20, HBJZ-44-2021, and JSZL-2018 on PK-15 cells

| PRV strains | Year of isolation | Country of isolation | Stable titers (TCID_50/_0.1mL) |
| --- | --- | --- | --- |
| JSZL-2018 | 2018 | China | 10^7.46^ |
| HBJZ-44-2021 | 2021 | China | 10^7.50^ |
| HuB20 | 2020 | China | 10^7.42^ |
| HeN21 | 2021 | China | 10^7.41^ |
