# Supplementary material for: Characterization of Pseudorabies Virus Associated with Severe Respiratory and Neuronal Signs in Old Pigs
Source: Transbound Emerg Dis. 2023 Feb 28;2023:8855739. doi: 10.1155/2023/8855739 (PMC12017139; doi:10.1155/2023/8855739)
Supplement: Supplementary Materials — Figure S1: Cytotoxic effects caused by different PRV strains on different types of cells. Figure S2: Damages of main organs of fattening pigs caused by different PRV strains. Figure S3: Amino acid sequences alignments of main PRV glycoproteins (gB, gC, gD, gE, gG, gH, gL, gM, gN, and gK) between different PRV strains. Table S1: Reference PRV genome sequences used in this study. Table S2: Stable titers of PRV strains HeN21, HuB20, HBJZ-44-2021, and JSZL-2018 on PK-15 cells. Table S3: Comparisons of viral loads in different organs of pigs between different PRV-challenging groups. Table S4: Pathological injury scores of main organs of mice caused by different PRV strains. Table S5: Pathological injury scores of main organs of fattening pigs caused by different PRV strains. [file 8855739.f1.zip › Table S3 (1).docx]

**Table S3.** Comparisons of viral loads in different organs of pigs between different PRV-challenging groups

| Groups | Organs | | | | | |
| --- | --- | --- | --- | --- | --- | --- |
|  | Heart | Liver | Spleen | Lung | Kidney | Brain |
| HeN21 vs. HuB20 | NS | NS | NS | NS | NS | NS |
| HeN21 vs. HBJZ-44-2021 | NS | NS | NS | NS | NS | NS |
| HeN21 vs. JSZL-2018 | NS | *P*= 0.032 | NS | *P*= 0.044 | NS | *P*= 0.006 |
| HeN21 vs. HuB1/CHN2017 | NS | *P*= 0.015 | NS | *P*= 0.003 | *P*= 0.033 | *P*= 0.027 |
| HeN21 vs. SMX-2012 | *P*< 0.001 | *P*= 0.008 | *P=* 0.038 | *P*< 0.001 | *P=* 0.020 | *P*< 0.001 |
| HeN21 vs. Bartha-K61 | *P*< 0.001 | *P*< 0.001 | NS | *P*< 0.001 | *P*< 0.001 | *P*< 0.001 |
| HeN21 vs. Ea | *P*< 0.001 | *P*< 0.001 | NS | *P*< 0.001 | *P*< 0.001 | *P*< 0.001 |
| HuB20 vs. HBJZ-44-2021 | NS | NS | NS | NS | NS | NS |
| HuB20 vs. JSZL-2018 | NS | *P=* 0.002 | *P=* 0.033 | *P=* 0.025 | NS | *P*< 0.001 |
| HuB20 vs. HuB1/CHN2017 | NS | *P*< 0.001 | *P*< 0.001 | *P*< 0.001 | *P*= 0.027 | *P*< 0.001 |
| HuB20 vs. SMX-2012 | *P*< 0.001 | *P*< 0.001 | *P*< 0.001 | *P*< 0.001 | *P*= 0.023 | *P*< 0.001 |
| HuB20 vs. Bartha-K61 | *P*< 0.001 | *P*< 0.001 | *P*< 0.001 | *P*< 0.001 | *P*< 0.001 | *P*< 0.001 |
| HuB20 vs. Ea | *P*< 0.001 | *P*< 0.001 | *P*< 0.001 | *P*< 0.001 | *P*< 0.001 | *P*< 0.001 |

NS: no significance
