# Supplementary material for: Characterization of Pseudorabies Virus Associated with Severe Respiratory and Neuronal Signs in Old Pigs
Source: Transbound Emerg Dis. 2023 Feb 28;2023:8855739. doi: 10.1155/2023/8855739 (PMC12017139; doi:10.1155/2023/8855739)
Supplement: Supplementary Materials — Figure S1: Cytotoxic effects caused by different PRV strains on different types of cells. Figure S2: Damages of main organs of fattening pigs caused by different PRV strains. Figure S3: Amino acid sequences alignments of main PRV glycoproteins (gB, gC, gD, gE, gG, gH, gL, gM, gN, and gK) between different PRV strains. Table S1: Reference PRV genome sequences used in this study. Table S2: Stable titers of PRV strains HeN21, HuB20, HBJZ-44-2021, and JSZL-2018 on PK-15 cells. Table S3: Comparisons of viral loads in different organs of pigs between different PRV-challenging groups. Table S4: Pathological injury scores of main organs of mice caused by different PRV strains. Table S5: Pathological injury scores of main organs of fattening pigs caused by different PRV strains. [file 8855739.f1.zip › Table S4 (1).docx]

**Table S4.** Pathological injury scores of main organs of mice caused by different PRV strains.

| PRV strains of inoculation | | Ea | Bartha-K61 | SMX-2012 | HuB1/CHN2017 | HuB20 | HeN21 | JSZL-2018 | HBJZ-44-2021 |
| --- | --- | --- | --- | --- | --- | --- | --- | --- | --- |
| Brain sections | Inflammatory cell infiltration | 0 | 0 | 0 | 0 | 0 | 0 | 1 | 0 |
|  | Neuronal degeneration，necrosis | 0 | 0 | 0 | 1 | 1 | 1 | 0 | 1 |
|  | Glial cell lesion (increased, nodular) | 0 | 0 | 0 | 1 | 1 | 1 | 1 | 1 |
|  | Neuronal loss | 0 | 0 | 0 | 0 | 1 | 1 | 0 | 1 |
|  | Hemorrhage | 0 | 0 | 0 | 0 | 0 | 1 | 1 | 0 |
|  | Hyperplasia | 0 | 0 | 1 | 1 | 1 | 1 | 1 | 0 |
|  | Capillary proliferation | 1 | 1 | 1 | 1 | 1 | 1 | 1 | 1 |
|  | Total | 1 | 1 | 2 | 4 | 5 | 6 | 5 | 4 |
| Lung sections | Alveolar epithelial cells proliferated and exfoliated | 0 | 0 | 0 | 0 | 0 | 0 | 0 | 0 |
|  | Alveolar wall capillary hemorrhage, congestion | 1 | 1 | 1 | 1 | 0 | 1 | 1 | 1 |
|  | Cell infiltration in pulmonary alveoli interspace | 0 | 0 | 0 | 0 | 1 | 1 | 1 | 0 |
|  | Alveolar walls thickened | 0 | 0 | 1 | 1 | 1 | 1 | 0 | 1 |
|  | Total | 1 | 1 | 2 | 2 | 2 | 3 | 2 | 2 |
