# Supplementary figures and images for: Characterization of Pseudorabies Virus Associated with Severe Respiratory and Neuronal Signs in Old Pigs
Source: Transbound Emerg Dis. 2023 Feb 28;2023:8855739. doi: 10.1155/2023/8855739 (PMC12017139; doi:10.1155/2023/8855739)

**A**

**PK-15**

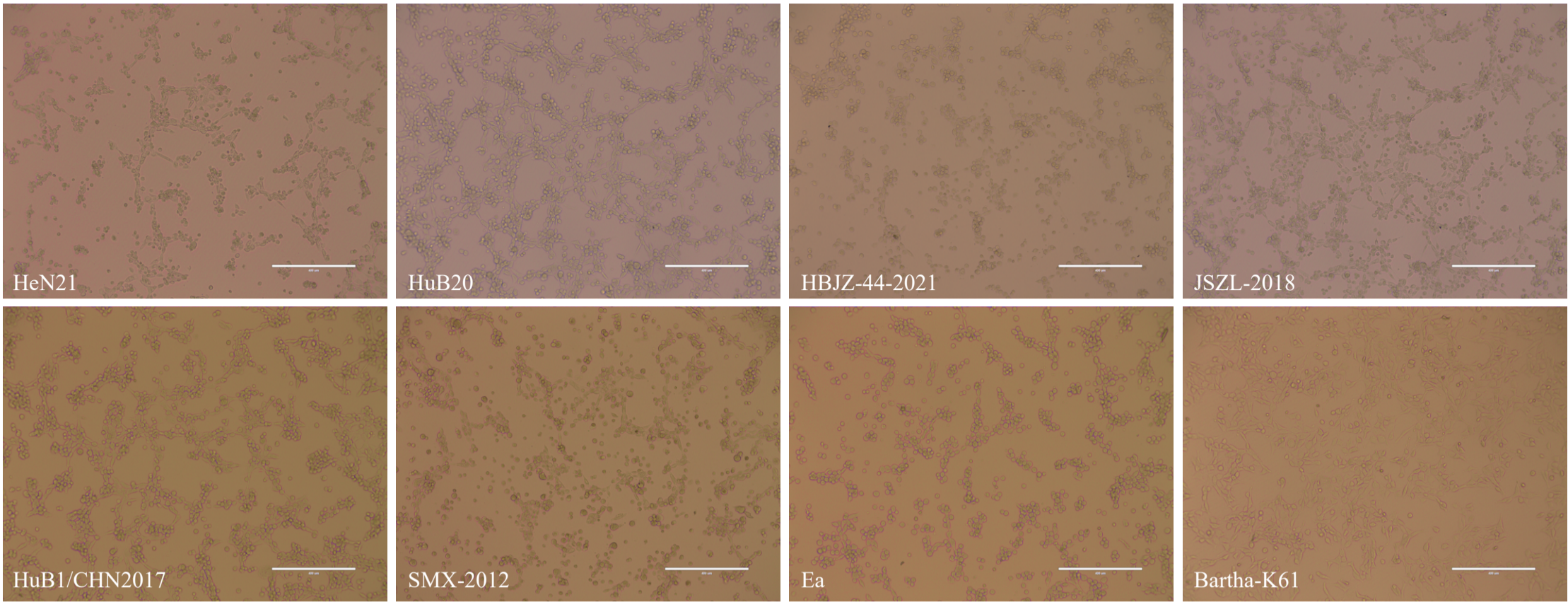

**B**

**Vero**

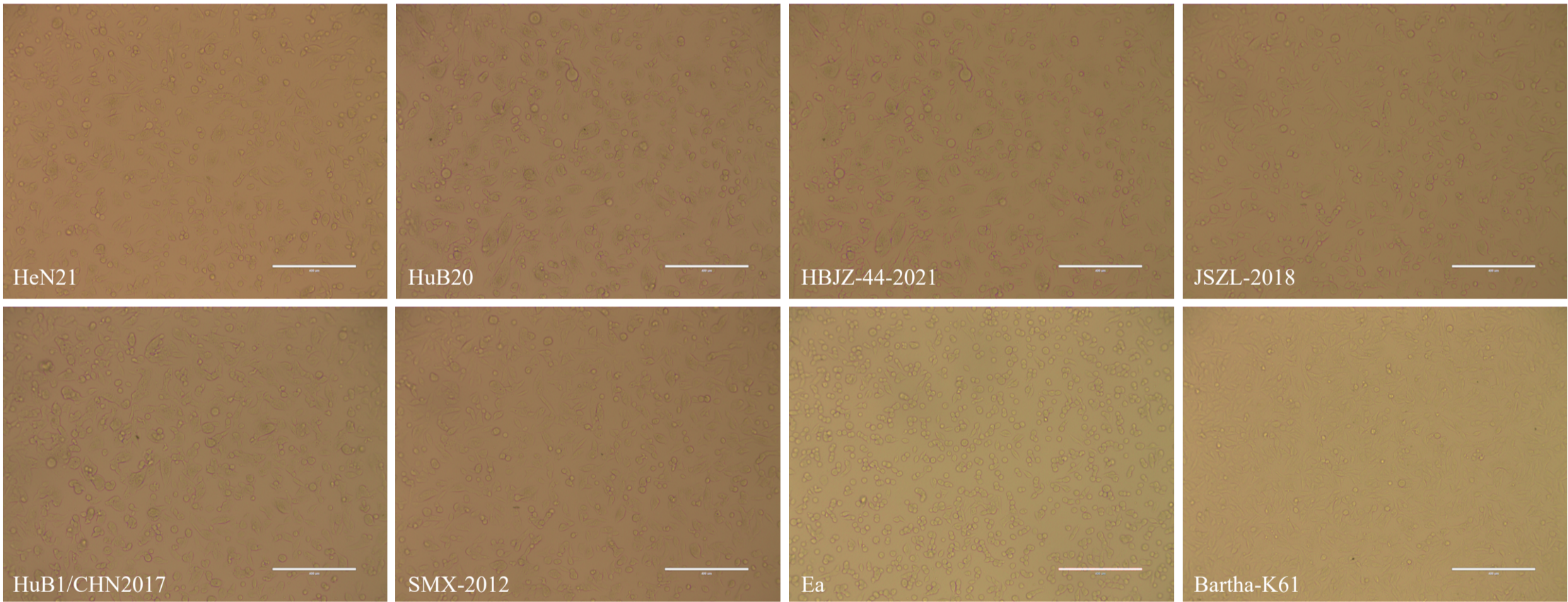

**C**

**SK-N-SH**

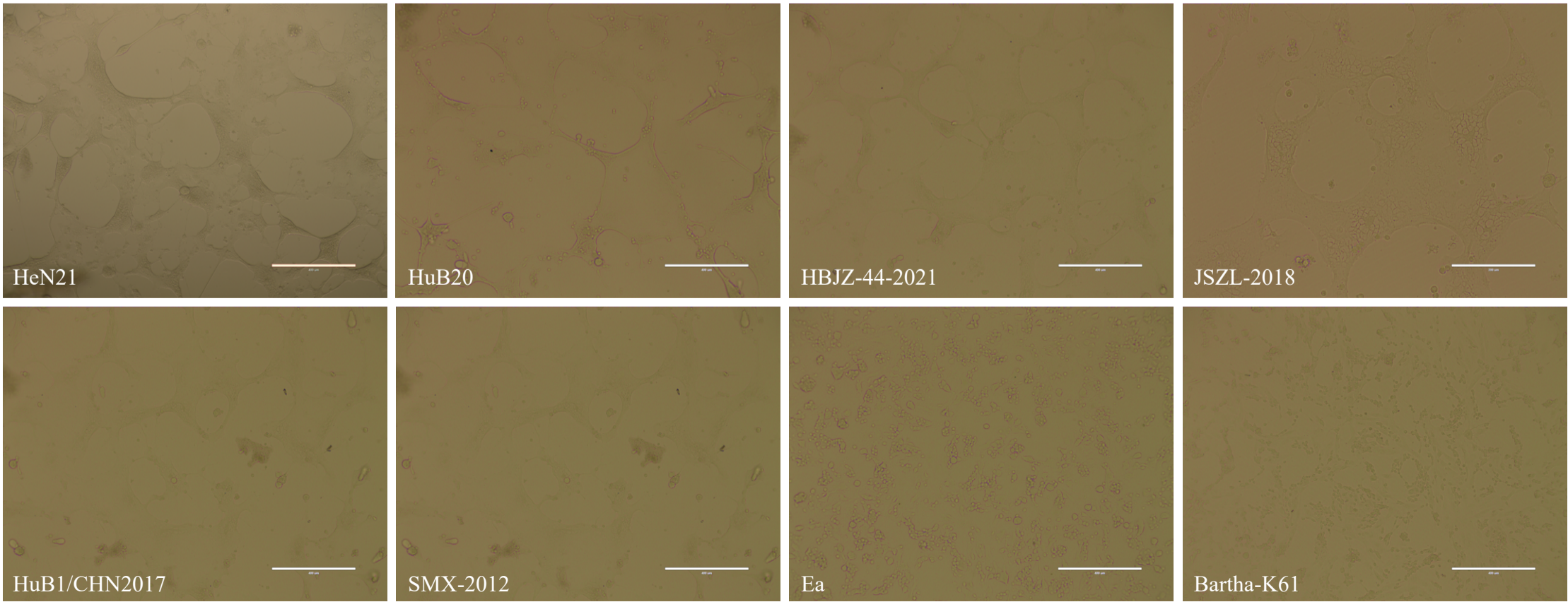

Supplement: Supplementary Materials — Figure S1: Cytotoxic effects caused by different PRV strains on different types of cells. Figure S2: Damages of main organs of fattening pigs caused by different PRV strains. Figure S3: Amino acid sequences alignments of main PRV glycoproteins (gB, gC, gD, gE, gG, gH, gL, gM, gN, and gK) between different PRV strains. Table S1: Reference PRV genome sequences used in this study. Table S2: Stable titers of PRV strains HeN21, HuB20, HBJZ-44-2021, and JSZL-2018 on PK-15 cells. Table S3: Comparisons of viral loads in different organs of pigs between different PRV-challenging groups. Table S4: Pathological injury scores of main organs of mice caused by different PRV strains. Table S5: Pathological injury scores of main organs of fattening pigs caused by different PRV strains. [file 8855739.f1.zip › Figure S1.pdf]

**HeN21**

**HuB20**

**SMX-2012**

**Mock**

**Lung**

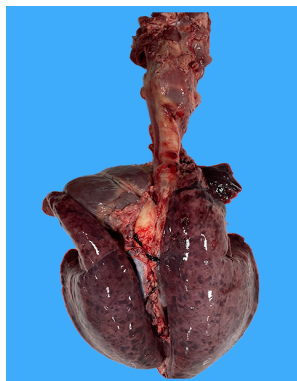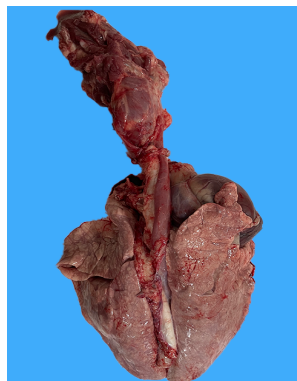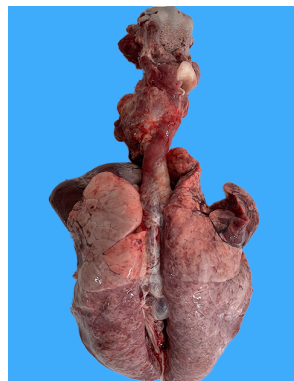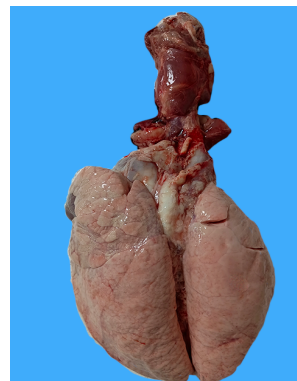

**Brain**

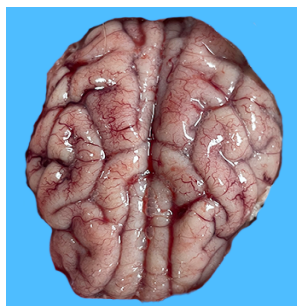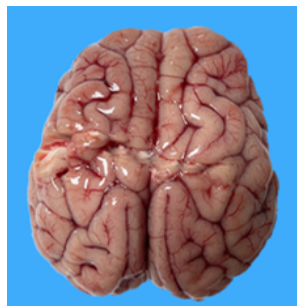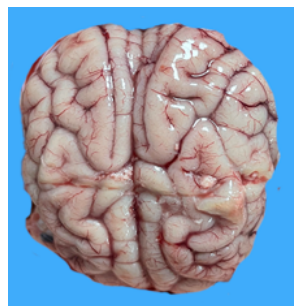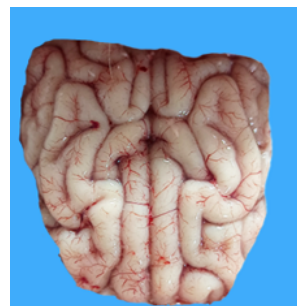

**Liver**

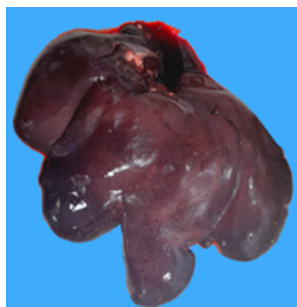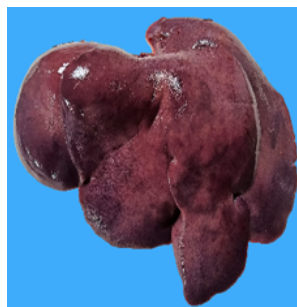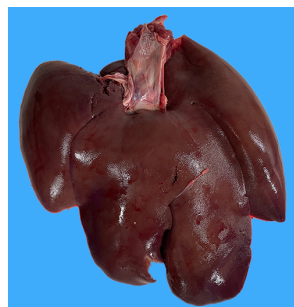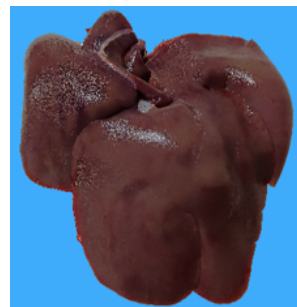

**Tonsil**

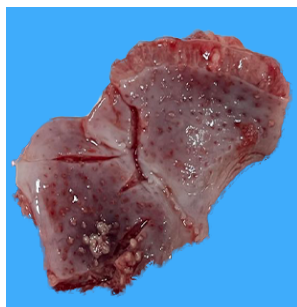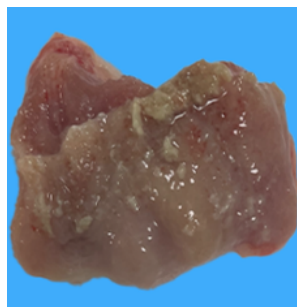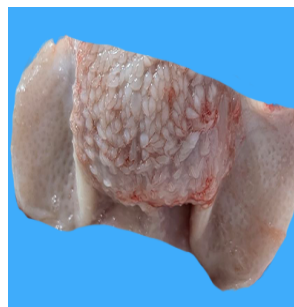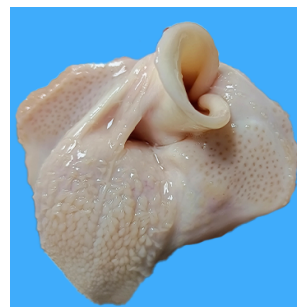

Supplement: Supplementary Materials — Figure S1: Cytotoxic effects caused by different PRV strains on different types of cells. Figure S2: Damages of main organs of fattening pigs caused by different PRV strains. Figure S3: Amino acid sequences alignments of main PRV glycoproteins (gB, gC, gD, gE, gG, gH, gL, gM, gN, and gK) between different PRV strains. Table S1: Reference PRV genome sequences used in this study. Table S2: Stable titers of PRV strains HeN21, HuB20, HBJZ-44-2021, and JSZL-2018 on PK-15 cells. Table S3: Comparisons of viral loads in different organs of pigs between different PRV-challenging groups. Table S4: Pathological injury scores of main organs of mice caused by different PRV strains. Table S5: Pathological injury scores of main organs of fattening pigs caused by different PRV strains. [file 8855739.f1.zip › Figure S2.pdf]
